# Unique Rhythmic Signatures in Arrhythmic Birdsong

**DOI:** 10.1111/nyas.70452

**Published:** 2026-11-13

**Authors:** Anna N. Osiecka, Javier Oñate‐Casado, Tereza Petrusková, Adrián Barrero, Václav Beran, Tereza Kubíková, Cristian Pérez‐Granados, Michal Porteš, Juan Traba, Adam Petrusek, Lara S. Burchardt

**Affiliations:** ^1^ Institute for Theoretical Biology Humboldt University of Berlin Berlin Germany; ^2^ Animal Communication and Urban Ecology Group Max Planck Institute for Biological Intelligence Seewiesen Germany; ^3^ Faculty of Science, Department of Ecology Charles University Prague Czechia; ^4^ Terrestrial Ecology Group, Department of Ecology Universidad Autónoma de Madrid Madrid Spain; ^5^ Centro de Investigación en Biodiversidad y Cambio Global Universidad Autónoma de Madrid Madrid Spain; ^6^ Municipal Museum of Ústí nad Labem Ústí nad Labem Czechia; ^7^ ALKA Wildlife o.p.s. Lidéřovice Czechia; ^8^ Biodiversity Management and Conservation Programme Forest Science and Technology Center of Catalonia, CTFC Lleida Spain; ^9^ Regional Office České Středohoří Protected Landscape Area Administration Nature Conservation Agency of the Czech Republic Litoměřice Czechia

**Keywords:** acoustic rhythms, *Anthus campestris*, geographic variation, integer ratios, tawny pipit, vocal identity

## Abstract

Animal rhythms are gaining increasing attention in the studies of behavior and musicology. Recently, it has been shown that rhythm itself can be used as an information coding channel. Now we ask: Does this hold true for arrhythmic sequences? To answer that, we analyzed songs of the tawny pipit (*Anthus campestris*), a migratory songbird known for its simple, arrhythmic songs. From focal recordings of 341 males across 6 European populations, we extracted an extensive set of rhythmic and temporal indices. We show that despite their apparent randomness, tawny pipit songs carry individual signatures—modern rhythmic indices such as beat precision and integer ratios proved among the most individually distinct parameters. Strikingly, we also uncover population‐specific temporal patterns across the species' European range that have not been revealed by conventional spectral analyses. This study is the first to demonstrate geographic scale differences in birdsong rhythm, and to show that rhythmic analysis can provide useful descriptions of arrhythmic sequences.

## Graphical abstract

Songs of tawny pipits (*Anthus campestris*) appear clearly arrhythmic at the species level or within single songs. However, individual rhythms are highly repeatable and population patterns emerge. Modern rhythmic indices are good descriptors of seemingly arrhythmic sequences.

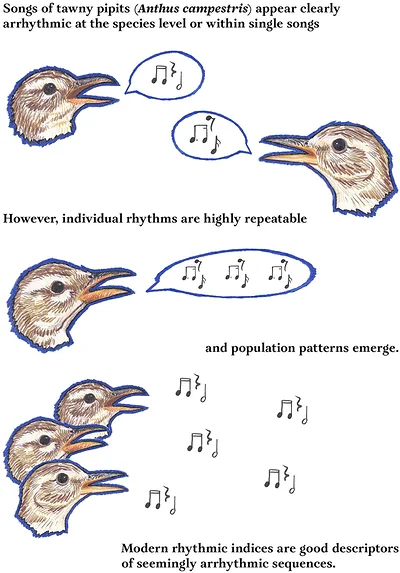

## 1 Introduction

Animal sounds, and birdsong in particular, have always accompanied and fascinated humans. Within the Western musical tradition alone, written examples of avian‐inspired music date back at least to the 13th century (such as the Wessex *Sumer is icumen in* rota evoking cuckoo calls, with a preserved manuscript from 1261 to 1264), and is still with us in the TikTok era and Sy Wylie's Bird Beats. In fact, some of the early attempts to describe avian sounds rely on onomatopoeia and mimicking their temporal patterns, including mnemonic phrases to recognize species (e.g., *cilp‐calp* for the common chiffchaff, *Phylloscopus collybita*, in Polish and Czech, or *a little bit of bread and no cheese, please* for the yellowhammer, *Emberiza citrinella*, in English). Simple music notation attempts also tried to capture the calls' or songs' overall structure—these range from Athanasius Kircher's 1650 *Musurgia Universalis* musical drawings of hens to Friederich Goethe's *Möwenmusik* (seagull music), showcasing the seabird calls of the German Baltic coast [1].

These long‐standing attempts to capture avian temporal structure underscore avian sounds not only as spectral objects but also as patterned sequences in time. Nonetheless, with the development of modern signal analysis methods, quantitative analyses in bioacoustics have historically privileged spectral or simple temporal descriptors, leaving rhythmic organization comparatively underexplored. Rhythm is now coming back into focus in the studies of animal behavior thanks to the development of new, robust rhythm analysis methods (such as rhythmic ratios or beat precision [2, 3, 4]). Modern rhythmic indices provide sequence‐level descriptors that quantify relative timing between elements, going beyond measures based solely on mean durations, tempos, or inter‐onset intervals (IOIs).

Recently, we have shown that modern rhythmic indices [4] (as opposed to simple descriptions of the temporal structure) can not only describe a sequence but also prove reliable and important sources of information for isochronous (equally distributed in time [5]) and *rallentando* (slowing down [6]) vocal sequences. Can the same methods be used for signals that appear irregular or unpredictable? This distinction is crucial because many animal displays lack overt periodicity, yet exhibit consistent internal temporal relationships that may encode relevant information (such as caller's identity in the seemingly arrhythmic vocal displays of the tambourine dove, *Turtur tympanistria* [7]). A question thus arises: can modern rhythmic indices provide useful information for arrhythmic sequences? In other words, if we approach rhythm as a description of patterned temporal relationships of all elements in a sequence to each other [4], can these relationships be used to describe constructs without predictable patterns?

Testing this requires species whose songs are variable, and rhythmically unpatterned. Tawny pipits (*Anthus campestris*, Passeriformes: Motacillidae) provide an ideal system: males of this migratory songbird produce short (up to 0.5 s) territorial songs that vary substantially among individuals [8, 9, 10] yet in most cases lack obvious repeated temporal patterns (Figure 1) [10]. Additionally, with prominent element overlaps (a “two‐voice” effect [11]), tawny pipit song presents an interesting question as to what constitutes a unit of the temporal structure.

**FIGURE 1:**
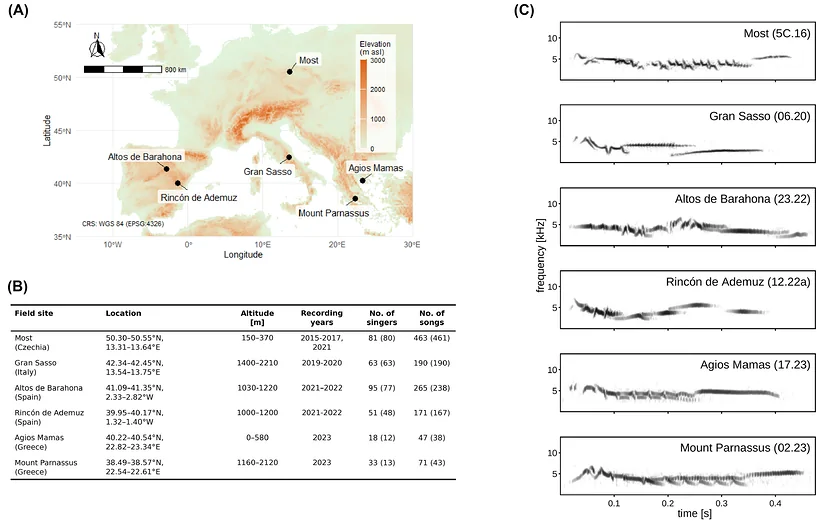
(A) Study locations covering the species' breeding grounds in Southern and Central Europe. Raster data obtained via *geodata* package [14]. (B) Field site details and numbers of individual males and total songs recorded per site (in parentheses, males with at least three recorded songs). (C) Spectrograms of sample songs from each population (plotted in Avisoft [15] with the following settings: Hamming Window, FFT 256, Frame size 100%, *y*‐scale enlargement 2, temporal overlap 87.5%). Individual identifiers of males are shown in parentheses.

This study explores the rhythmic structure of tawny pipits' songs, lacking obvious rhythmic organization, as an information channel for singer identity and population of origin, by revisiting a vast recording library collected across their European breeding range (Figure 1) [12].

## 2 Methods

### 2.1 Ethics Approval

This project was based on already available data (most of which was previously published [9, 12], including a representative sample of acoustic data available at [13]), and as such no specific permits were necessary. The original recordings were collected over the breeding seasons 2015–2022 using noninvasive distance sampling methods (see methodological details in Ref. [12]).

### 2.2 Fieldwork and Recordings

Recordings were collected in six European breeding sites of the species (Figure 1). All these locations were characterized by similar open habitats preferred by the species, with low or limited vegetation and scattered rock formations, bushes, or trees used as singing posts by the territorial males [12]. In most cases, each male was only recorded once, and recordings at the same localities in different years covered different males. The subset collected in Most (Czech population) included numerous males recorded repeatedly within and between seasons—this is because the original study focused on the applicability of individual acoustic monitoring [9].

Recordings were made using Sennheiser ME‐67 shotgun directional microphones and Marantz PMD 661 recorders (16 bit, 44.1 kHz sampling rate) in a focal follow regime with vocal notes to identify the recorded singer (i.e., the recordist would make a clear comment after each time the observed animal sang).

Even though male and female tawny pipits are apparently identical in plumage [10] and spectral structure of their song [9], the sex of the singing individual can be reliably deduced considering the behavioral context during the breeding season [9]. Only males defend their territories by singing. Female songs, performed in different contexts [9, 10, 16] and recorded in very limited numbers in the Czech population [9], were excluded from further analyses.

### 2.3 Data Analysis

#### 2.3.1 Data Annotation and Rhythmic Indices

All recordings were annotated by J.O.‐C. in Luscinia (v. 2.24.09.12.02) [17] and most of the data previously published [9, 12]. Luscinia is a bioacoustic software that allows detailed annotation of vocalizations by manually tracing spectral contours on spectrograms with a brush tool (see Figure S1 for examples of annotated songs). This approach is particularly useful for sounds with elements overlapping on the time axis, and field recordings with background noise, or two‐voice vocalizations with overlapping elements, as it allows the user to trace and reliably measure each element separately and ensures precise time measurements, even in songs with substantial overlap between elements or irregular spectral patterns. Thanks to this, we were able to identify song elements of these seemingly chaotic sounds.

The same spectrogram settings were applied to all recordings (5 ms frame length, 1 ms time‐step, Gaussian window function, 220 spectrograph points, and 80% overlap). When needed, we removed the noise and improved the signal by altering the dereverberation (100%), dereverberation range (50 ms), dynamic range (40 dB), and high pass threshold (1000 Hz) settings. We then characterized the parameters for every single element within a song, considering only recordings of sufficient quality in which song elements could be clearly distinguished from background noise. All elements were considered (e.g., in the case where long tonal sounds overlapped with a series of short pulses, each element was marked separately; see Figure 2B).

**FIGURE 2:**
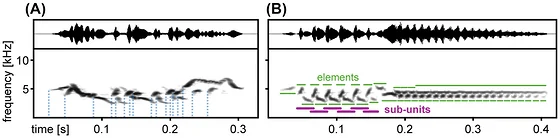
Sample spectrograms and waveforms of two contrasting tawny pipit songs, without (A) and with (B) apparent organization into sub‐units. Both songs were recorded in the Czech population in the same year. Element onset times (A, blue dotted lines) refer to the start times of each element. Elements (B, green) are distinct spectral shapes, in some cases apparently organized into subunits (B, violet)—elements organized in a repeated pattern, absent from most songs (see definition in Table 1). Note that the amplitude peaks do not reliably reflect element onset times. Subunits are not quantified in any way. Spectrograms were plotted in Avisoft [15] for illustration purposes only, with the following settings: Hamming Window, FFT 256, Frame size 100%, *y*‐scale enlargement 2, temporal overlap 87.5%.

The start and end times of these elements were then exported from Luscinia and used to calculate a set of temporal and rhythmic indices (Table 1) using a custom made script (based on [4, 5, 18]) in R v. 4.4.2 [19]. Rhythmic indices were calculated for all pairs of element onset times, independently of temporal overlaps. Element definition based on separation by silence is standard and follows best practices recommendations [20]. Similarly, while approaches based on, for example, energy peaks exist, onset‐based integer ratios are a commonly used measure, also for sequences with fine temporal resolution, which allows excellent comparative possibilities across species (e.g., [3, 21, 22, 23, 24]). Duration‐based integer ratios are first used in this study.

**TABLE 1:** Definitions of the concepts behind rhythmic parameters calculated for each song. For brevity, more commonly used concepts, for example, number of elements, as well as the specific measures for each parameter (minimum, maximum, mean, coefficient of variation), are not explicitly defined.

Abbreviation	Definition
Song	A single vocal territorial display produced by a tawny pipit, bounded by silence before and after the vocalization. Note that this refers to the concept of song as used in this study—a definition that covers all song concepts is not possible [26, 27].
Element	A continuous spectral trace on a spectrogram, separated from other elements by a visible gap in frequency or by a distinct change in spectral structure. Elements may overlap in time if they occupy different frequency bands; each such trace is annotated separately.
Sub‐unit	A group of two or more consecutive elements that appear to form a coherent local pattern (e.g., a repeated motif) within a song, identified post hoc during analysis rather than defined a priori. Used here as a descriptive term for local organization that does not constitute a full song pattern.
Silence	The time interval between the end of one element and the start of the next element within a song. When elements overlap, silence is calculated as a negative value.
Duration	The temporal extent of a single element, measured from its start time to its end time (in ms).
IOI	Inter‐onset‐interval, that is, the time between the start of one element, and the start of the following element. All elements are used, independently whether they overlap or not.
IOI beat	Beat in Hz calculated based on the IOI [23].
Beat precision	Indicates how well individual elements in a sequence fit a theoretical beat. Calculated as the ratio between the actual deviation from a theoretical perfectly isochronous beat to the next theoretical beat and the maximum deviation for the calculated best‐fitting beat. First defined as universal goodness of fit (ugof [28]) $bp=\frac{\left|\Delta \right|}{\Delta_{\max}}.\;\left(1\right)$
Rk based on IOI (local)	Integer ratios based on inter‐onset‐intervals between elements, calculated as a ratio between the IOI's duration and the sum of this and the following IOI's duration [2]. Note that the mean value of the same calculation is sometimes described as “mean acceleration rate” [29]. $r_{k}=\frac{{\mathrm{ioi}}_{-1}}{{\mathrm{ioi}}_{-1}+{\mathrm{ioi}}_{1}}.\;\left(2\right)$
Rk based on duration (local)	Integer ratios based on element duration, calculated as a ratio between the element's duration and the sum of this and the following element's duration [2]. Note that the mean value of the same calculation is sometimes described as “mean acceleration rate” [29].
Rk based on IOI (global)	Integer ratios [2] based on inter‐onset‐intervals between elements, calculated not only for adjacent intervals in the sequences but also for all interval pairs of a sequence [4] $r_{k}^{\left(n,m\right)}=\frac{{\mathrm{ioi}}_{n}}{{\mathrm{ioi}}_{n}+{\mathrm{ioi}}_{m}},\;\text{for all}\;n,m\in \left\{1,\cdots ,N\right\},\;n\neq m.\;\left(3\right)$
Rk based on duration (global)	Integer ratios [2] based on element duration, calculated not only for adjacent intervals in the sequences but also for all interval pairs of a sequence [4]

We wish to acknowledge that, like any annotation method, manual annotation in Luscinia may introduce some measurement uncertainty. In particular, repeated songs from the same individual can appear nearly identical when inspected visually as spectrograms, while the temporal measurements obtained after annotation may still show some variation. This may arise from small differences in boundary placement, signal quality, spectrogram settings, and observer judgment. While the annotation of the entire dataset will naturally reflect annotator profile and choices, it also excludes issues of annotator disagreement [25]. See Supplementary Material S1 for an additional assessment of inter‐annotator variation.

### 2.4 Rhythmic Organization

We simulated 1000 random songs with 5–30 elements, randomly generating IOIs and element durations within the 5%–95% percentile range of the original observed IOIs. Integer rations (both local and global, based on IOIs and durations) were calculated. These were compared with the observed integer ratios to assess differences in distribution using (1) two‐sided Kolmogorov–Smirnov test, function *ks.test*, package *stats*), and (2) Q–Q plots (function *qqplot*, base R).

### 2.5 Classification to Individual and Population

To investigate the general patterns for the species, all songs (1207 songs from 341 individuals) were kept. For classification to individual and population, we retained only individuals with at least three recorded songs (i.e., kept all songs of all individuals for whom at least three good quality songs were recorded), yielding 1137 songs from 293 individuals.

We then confirmed that the data were appropriate for factor analysis by conducting the Kaiser–Meyer–Olkin test (*KMO* function of *EFAtools* package) [30]. With a KMO score of 0.72, the result was middling, and therefore the data was suitable to conduct a principal components analysis (PCA), which was done using the *prcomp* function of the *stats* package [19]. We only retained the principal components with eigenvalues > 1 for further analysis (Kaiser's criterion; seven principal components were retained).

To investigate the individuality of songs across populations, we performed a permuted discriminant function analysis (pDFA) [31], pooling all available songs from all individuals and from across the six populations (following the minimum song number criterion described above; 1137 songs in total). The pDFA with nested design was conducted using the *pDFA.nested* function of a script provided by Roger Mundry (based on the function *lda* of the *MASS* package [32]), using individuals' unique IDs as a test factor, and population as a restriction factor, and the retained principal component scores as input variables.

To investigate the geographical patterns in rhythmic structure, we performed a similar pDFA, with population code as the test factor and singers' ID as a control factor. A Supervised Uniform Manifold Approximation and Projection (S‐UMAP [33]) was created for visual investigation of this clustering using the *umap* function of *uwot* package [34], using all calculated indices (Table 1) as input data collapsed into three dimensions, 200 nearest neighbors (prioritize global over local structure), minimum distance 1 (for tight clusters), and Euclidean metrics.

### 2.6 Identity Coding

To report cross‐species comparable identity metrics [35], we calculated the Beecher's information statistic (Hs), which reports a level of individuality carried by an acoustic signal [36]. This was done by first collapsing the raw parameters by a PCA to obtain uncorrelated principal components, and then calculating the Hs based on all obtained principal components (functions *calcPCA* and *calcHs* of the *IDmeasurer* package [37])

To understand the relative importance of each of the rhythmic indices, we calculated the potential of individual coding (PIC [38]) of each parameter using the *calcPIC* function of the *IDmeasurer* package [37]. To verify that the outstanding PIC value for CV beat precision was not driven by a subset of highly variable individuals, we repeated the PIC calculation retaining only singers with CV beat precision below 0.7 (representative of the species, with most values in the range 0.65–0.70 and all above 0.64).

## 3 Results

No apparent rhythmic organization appears at a level of a single tawny pipit song (Figure 3). Similarly, no apparent rhythmic structure emerges in the species' song—that is, no repeated, predictable patterns emerge across the populations (Figure 1C). This was confirmed by plotting the distribution of integer ratios (rk) calculated for tawny pipit songs over randomly generated integer ratios (Figure 4). Integer ratios represent the relationship between each two elements (e.g., ratio of durations or IOIs), and are calculated for both adjacent and all element pairs in the sequence (see Table 2 for rhythm terms and definitions). While the Kolmogorov–Smirnov test indicated that each observed rk distribution statistically varied from random (*p* < 0.001), their alignment is very close (Figure 5).

**FIGURE 3:**
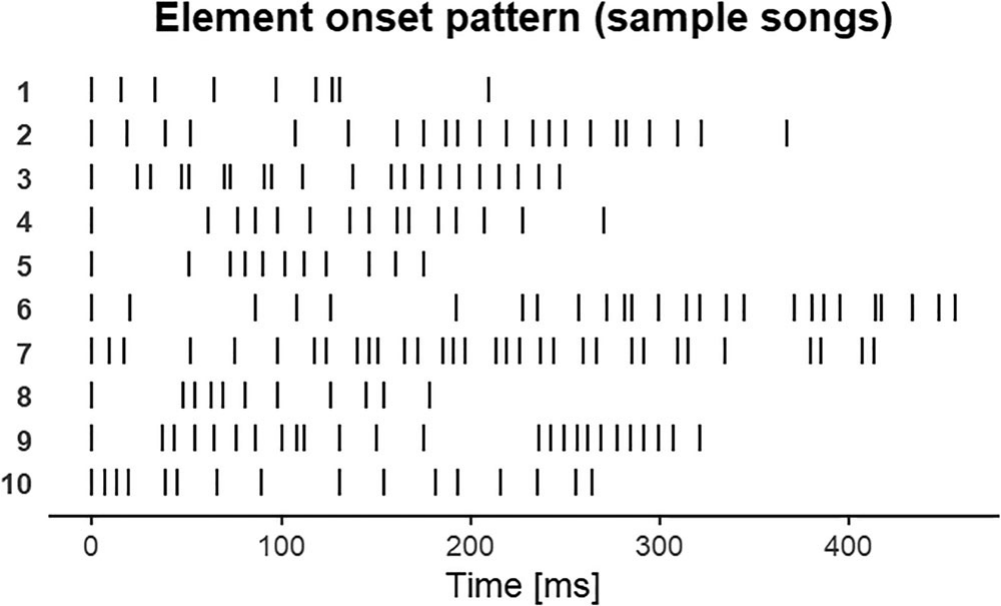
Element onset times in randomly selected sample songs of 10 random individuals from across all study populations indicate an apparent lack of species‐specific temporal structure to tawny pipit songs.

**TABLE 2:** Results of the permuted discriminant function analysis classifying each song to the singer across European populations.

	Individual	Population
Correctly classified [%]	75.3	54.6
Chance level for classified [%]	10.67	34.3
*p* value for classified	0.001***	0.001***
Correctly cross‐classified [%]	45.31	33.1
Chance level for cross‐classified [%]	0.8	17.8
Relative cross‐classification level	56.6	1.9
*p* value for cross‐classified	0.001***	0.001***

*Note*: The relative cross‐classification level indicates the ratio of correct cross‐ classif ication to its chance level. Both models are based on 293 individuals, 1137 songs, 6 populations.

*** = highly significant

**FIGURE 4:**
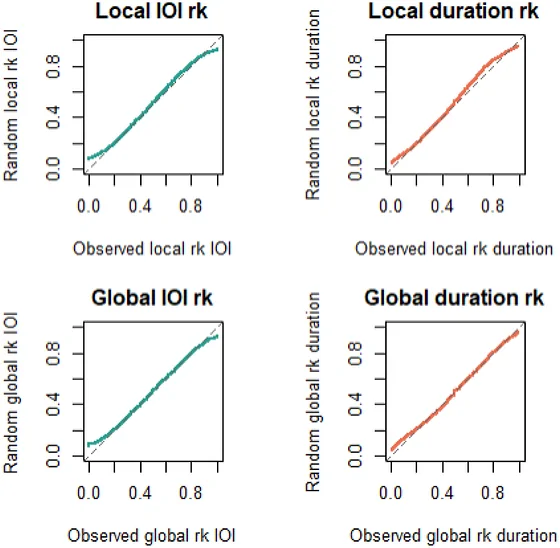
Q–Q plots comparing integer ratio distributions from the values observed and simulated on random data.

**FIGURE 5:**
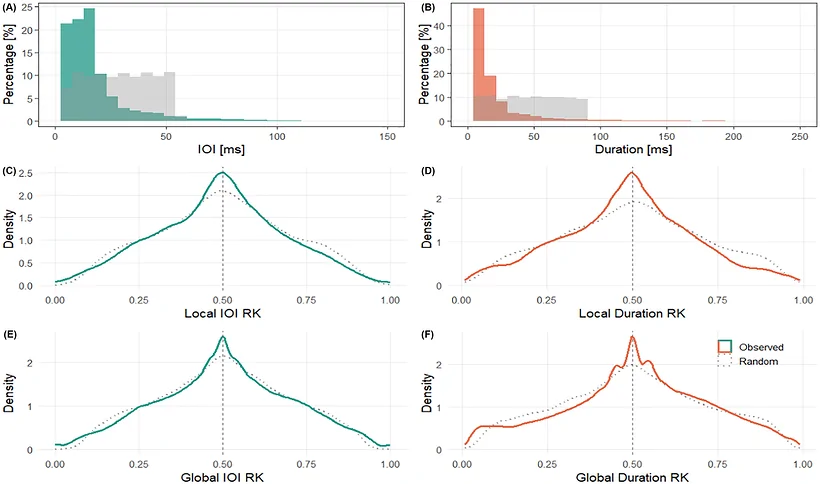
Distributions of inter‐onset‐intervals (IOIs) between elements (A), element durations (B), and rhythmic ratios plotted with random integer ratio (rk) values (created within the 5%–95% percentile range of the original IOIs, and durations; C–F). Clear peaks around a certain rk value represent the rhythmic organization of a sequence (or, like here, a lack of thereof). Solid lines represent observed, and dotted/gray randomly generated values, green—IOI, red—duration. Indices based on observed values largely follow the distribution of these based on randomly generated ones (C–F), indicating clearly random patterns with some sub organization into semi‐isochronous segments (0.5 density peaks within otherwise random distribution).

A closer investigation of the distributions suggests smaller sub‐units organized in local isochronous pattern (0.5 density peaks, Figure 5C–F). This aligns with what can be observed in some individual songs (see, e.g., Figure 6). In other words, while there is no global rhythmic pattern for the species nor clear repeated organization within a single song, integer ratios are not random and individual patterns clearly emerge.

In fact, pDFA, a robust cross‐classification method allowing to control for repeated measures of the same individuals and other factors potentially contributing to the songs' structure based on the rhythmic and temporal features [31], allowed us to classify songs to singers as well as to their populations of origin with strong statistical significance, although the pDFA classification performed better at an individual scale (Table 2).

S‐UMAP clustering indicated that while some populations are clearly differentiated, others partially overlap in the abstract acoustic space (Figure 7). Basic plots of raw feature distribution by population can be explored in the folder “plots” at https://osf.io/h2fpm/overview?view_only=60916d5355de494295a1e9612ca3d40c

**FIGURE 6:**
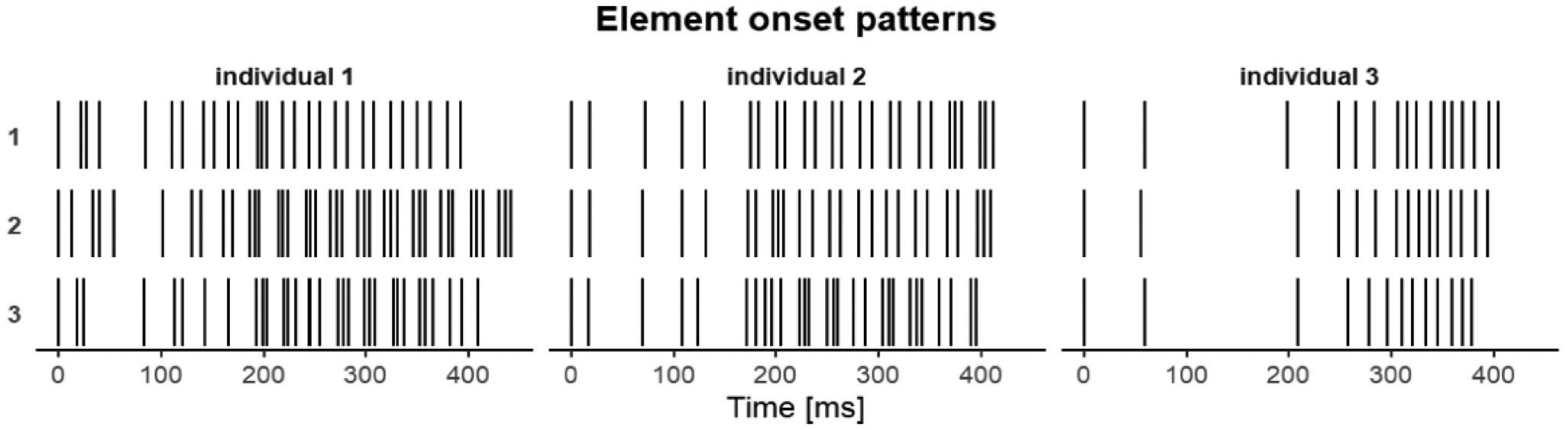
Element onset patterns for three randomly selected songs (rows) of three randomly selected individual singers (columns).

**FIGURE 7:**
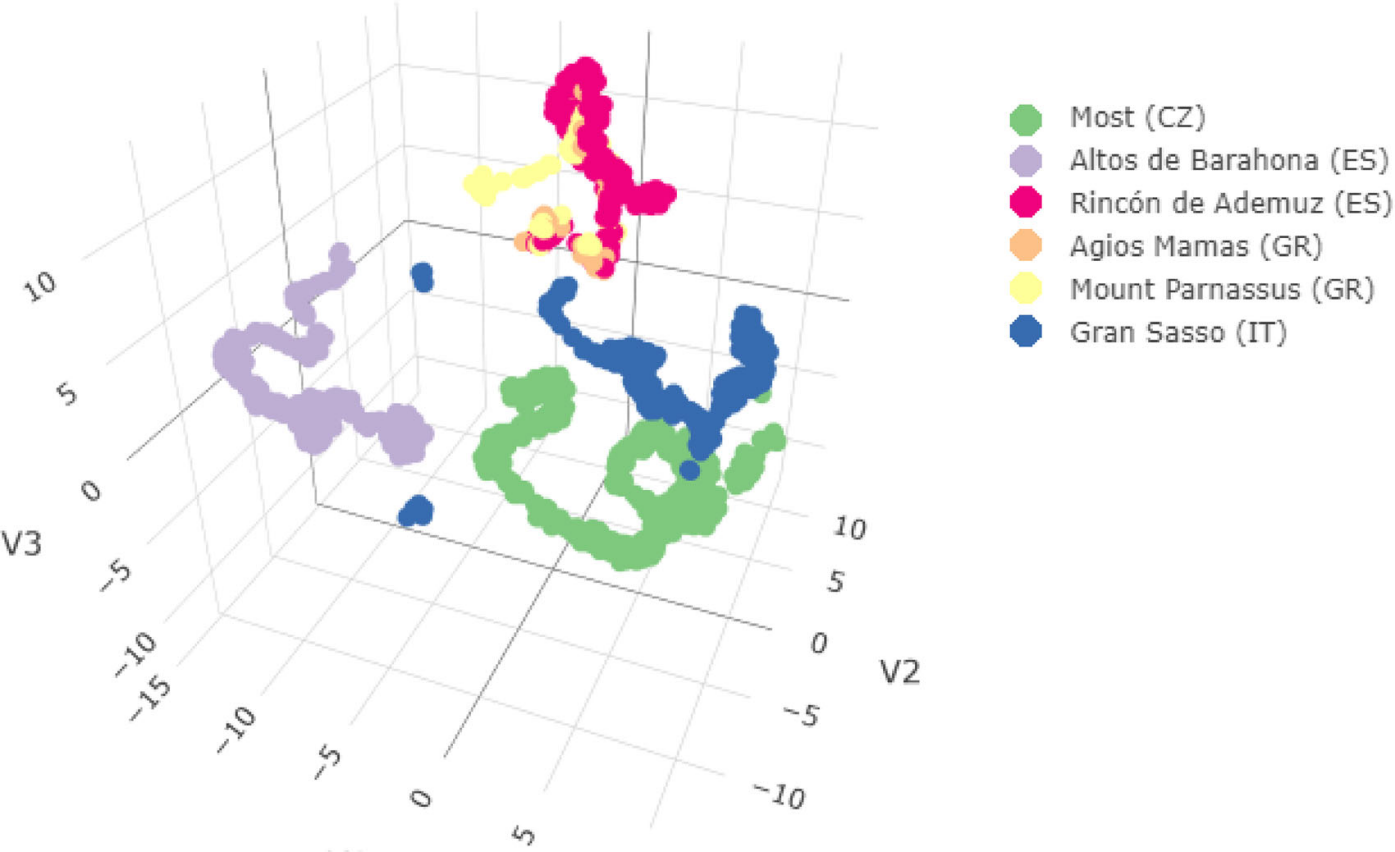
S‐UMAP represents how each song clusters in the abstract acoustic space (multidimensional sounds mapped based on their internal topology) to the singer's population of origin (in color). See the interactive clickable plot at https://osf.io/h2fpm/overview?view_only=60916d5355de494295a1e9612ca3d40c)

Based on the rhythmic indices, two identity metrics commonly used as best practice in individuality studies [35] were calculated, namely the Beecher's information statistic (Hs, [36])—an indicator of the overall individuality level in a chosen population, and the potential of identity coding (PIC) [38]—which measures the contribution of each parameter to the signal's individual traits. Hs reached 17.6. All temporal and rhythmic parameters indicated strong importance for identity coding (i.e., PIC over 1) [38], where the coefficient of variation of beat precision was by far the most important parameter, with the PIC value of 24.32 exceeding all others by an order of magnitude (Figure 8). An additional PIC calculation excluding the most variable individuals (CV beat precision > 0.7) confirmed this result (PIC = 26.8), ruling out an artifact of a few outlier birds.

**FIGURE 8:**
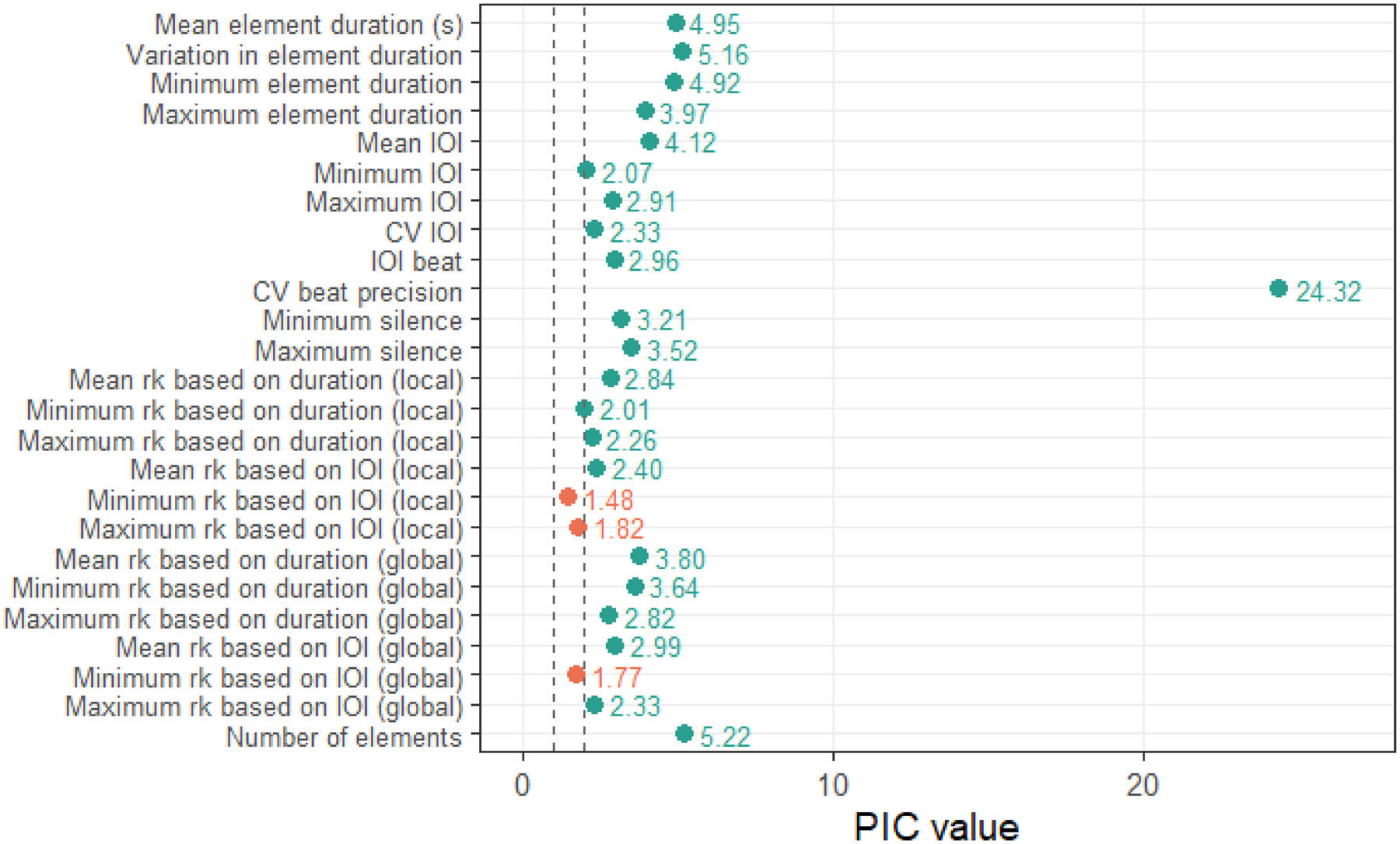
Potential of identity coding of the calculated temporal parameters. Dotted lines mark standard significance thresholds > 1 (red: important for identity coding) and > 2 (green: very important for identity coding). Note that each calculation is performed separately, and thus feature correlation is inconsequential here.

## 4 Discussion

This is the first time that rhythmic indices have been shown useful in describing arrhythmic structures, and a source of information within them. Here, the rhythmic structure of the tawny pipits' arrhythmic songs serve as channels carrying information on both the singer's identity and their population of origin.

### 4.1 The Question of Arrhythmicity

Throughout this text, we have decided to use the term “arrhythmic” when referring to the structure of the tawny pipits' songs. This follows the understanding of rhythm as “patterned relationships of elements in time” (after [4]) or “structure over time” (after [21, 39]). At the level of single songs or species‐wide pooling, these sequences lack apparent periodic structure, as their IOI distributions and integer ratio profiles approximate random distributions (Figure 5). Yet this characterization is analytically contingent. Similarities emerge clearly at the individual and population levels, indicating that “randomness” here denotes absence of particular expected regularities, rather than absence of structured temporal organization per se. We feel that “random” is a very strong and indeed unfitting word, and lean toward “arrhythmic” to reflect a lack of conformance to isochrony‐based rhythmic categories rather than lack of structure.

The term “arrhythmic” thus carries a paradox: what is classified as arrhythmic may instead reflect complex temporal organization not readily captured by isochrony‐biased approaches. This mirrors broader findings that temporal structures without isochronous pulse levels may appear chaotic when assessed through isochrony‐biased frameworks. Non‐isochronous meters such as the North Indian rupak tal, whose slow‐tempo cycles exceed perceptual limits for beat extraction and lack isochronous beat or subdivision levels, were historically misclassified or rendered invisible to Western metrical theory until culturally appropriate analytical frameworks were applied [40]. Similarly, the individually consistent but nonperiodic temporal relationships in tawny pipit songs may reflect organizational principles not captured by human measures designed for regularly patterned sequences, even though these measures proved good overall sequence descriptors.

What is clear is that the apparent arrhythmicity of tawny pipit songs cannot be equated with randomness, and that the choice of analytical framework fundamentally shapes what temporal structure becomes visible. Here, rhythmic patterns emerge through repetition of irregular structures, visible only in relationship to others (individuals, populations).

### 4.2 Rhythm as a Descriptor of Arrhythmic Sequences

Rhythm has been previously suggested [41], but to our knowledge, not used as an information coding channel in animal communication studies. Our own work used rhythmic descriptors as information sources in isochronous [5] and *rallentando* [6] vocal displays, where deviations from the perfectly isochronous beat could be expected to matter. Humans tend to find rhythm even when there is none [42]—which brings us to the assumption that the organization of apparently arrhythmic sequences is not meaningless. The fact that rhythmic methods can successfully describe arrhythmic sequences is promising for comparative, large‐scale studies. It allows us to examine the evolution of rhythm and vocal signals independently of their organization or predictability of their temporal patterning, and may ultimately contribute to our understanding of the evolution of language and music [41, 43, 44, 45].

Some previous work has indicated rhythm as a possible information channel while using other methodologies. Notably, corn crake (*Crex crex*) call sequences show a somewhat syntactic modification to meaning, dependent on what appears to be integer ratios (and was tested as unquantified “relative patterning” of the calls [46]). Previous studies have also found individual and/or sex‐specific patterns in simpler temporal descriptors in a wide range of taxa, such as the call or pulse rate (e.g., Cape gannet, *Morus capensis* [47], or elephant seals, *Mirounga leonina* [48]), exact note durations (e.g., tambourine dove [7], or spectral tarsiers, *Tarsius spectrumgurskyae* [49]), or exact [7] and mean syllable IOIs (e.g., indris, *Indri indri* [50], or the little auk, *Alle alle* [18]). Even very simple temporal parameters can matter, of course—for example, call duration is extremely important in emotional communication [51]. The difference between simple temporal descriptors (also used in the present study) and rhythmic indices that capture the relative patterning of elements should be made clear here.

Because rhythm is often treated as intuitively obvious, the term is frequently misused—underscoring the need to build on existing definitions rather than reinvent them. Best practices for robust methodologies and clear terminology have been outlined in several key texts (e.g., Refs. [3, 4, 52, 53]).

### 4.3 (Ar)rhythmic Individuality

We could correctly assign the songs to the individual singer at a level high above chance, with the relative cross‐classification level reaching 56.6%. While this is the first attempt focusing solely on temporal patterns, this individuality is not surprising: tawny pipit males use stereotyped songs [8], and assignment to song type is a reliable method of identification of individual singers in the field recordings [9]. While cross‐classified levels fall a bit lower, this is somewhat expected. Thinking in musical terms: if one collapses the entire staff of a musical piece into a single line, losing all the frequency information, it is not surprising that some pieces now look very similar.

What stands out is the very strong contribution of the coefficient of variation of beat precision to vocal identity, reaching an outstanding PIC over 33. It should be noted that PIC values are measures of variation, and as such do not have a maximum possible value. In fact, variation in rhythmic and temporal parameters seems to emerge as the most important indicator of individual features in some species (e.g., age in the Cape fur seal, *Arctocephalus pusillus* [54] or identity in the little auk (PIC for CV of syllable duration equal 17.2 [18]) and herein).

Here, we are looking at a “global individuality,” that is, the overall differences between birds at the European breeding grounds, but it should be noted that songbirds are known not only for the geographic variations of their songs but also within‐population diversity (e.g., [22, 55, 56, 57]). In other words, within a local population one may observe yet other patterns, possibly driven by the social structure, dispersal patterns or distance between neighboring singers (such as the apparently locally driven rhythmic diversification from the nearest singers in the yellowhammer [5]).

The functional significance of these individual rhythmic signatures cannot be explained by the present data. Tawny pipits return annually to breeding sites, where males must re‐establish territories and recognize neighbors. Individually specific temporal patterns likely contribute to the overall individuality of their songs [9], possibly reducing costly territorial conflicts [55] or playing a role in mate choice. What is particularly interesting is that the individual singers do not sing rhythmically identical songs but rather seem to maintain a rhythmic style with quite a substantial diversity (see, e.g., Figure 6). What is the source and meaning of this intra‐individual diversity, remains to be tested.

### 4.4 Geographical Variation and Segregation of Information

Previous work based on spectral shapes and measurements of tawny pipit songs suggested that the individuality in fact obscures geographic variation in the species [12]. Here, revisiting the same dataset from a purely rhythmic perspective, we show that geographical patterns do in fact exist in the species' song, even though they might not be recognizable in its spectral shape [12].

This suggests that larger‐scale song evolution in tawny pipits may have taken place primarily in the temporal dimension, while frequency related adjustments may function at a more local scale to diversify acoustic signals among individuals within populations. At what scales exactly this segregation might happen, however, remains unclear. In the common chiffchaff, simple temporal parameters such as the number of syllables and silence durations are some of the most prominent indicators of geographical song differences, and show a progressive eastward change across the Palearctic [58]. In chaffinches (*Fringilla coelebs*), repeated colonizations result in syntax, and thus expectedly rhythmic, simplification [59]. Examining more species over their geographic range would help us understand whether these large‐scale changes in the overall temporal patterning of songs and more local spectral adjustments might translate into a general pattern in birdsong evolution [60, 61].

Segregating specific pieces of information to specific sound dimensions or channels might allow the callers to encode such information more accurately [62, 63]. For example, the little auks use complex calls (*classic call* [64]) that carry strong identity signals in both spectral [18] and rhythmic [6] parameters, but contain information on partnership in the spectral and sex in the rhythmic dimension, respectively [6, 65]. For callers that need to signal their identity efficiently to acoustically mark their territories, like the tawny pipit, this might mean maximizing the level of variability (in this case, the number of recognizable individuals) within one channel or dimension.

Assignment of songs to their populations of origin, although less precise than in the case of individuals, was also significantly better than chance. This is likely a result of a rhythmic overlap between some populations, reflected in the S‐UMAP clustering (Figure 7), which revealed strong rhythmic diversification between three sites (Most, Gran Sasso and Altos de Barahona) with strong similarities between the other three (two Greek populations and the Spanish site of Rincón de Ademuz). While the similarity of geographically close Greek populations is not surprising, the overlap with the Spanish site may reflect the exclusion of spectral parameters (although note that no population differences were detected based on spectral contour alone [9]), differences in local population density, or sampling limitations. Are these similarities only apparent and artificial (e.g., an artifact of summary rhythmic descriptors)? Or might these three populations represent a more ancestral organization of their song and locally prefer rhythmic over frequency perfection during tutored learning [22]? Distinguishing these would require playback experiments or cross‐fostering studies beyond our present scope.

Tawny pipits are Palearctic migrants, wintering in the Sahel and moving to breed in their Northern ranges (for this study's population, Europe [66]). Eastern (Greek) and Western (Spanish) European populations are expected to migrate via Egypt and Gibraltar, respectively, and winter on the opposite sides of Sahel [67, 68], and are thus highly unlikely to meet and mix. One aspect that the Greek and Rincón de Ademuz populations do seem to have in common is an apparently lower local density (C.P.‐G., personal observations during fieldwork). While this might be expected to have some effect on local individuality [69], it remains unclear whether and how local density might influence geographic rhythmic patterns in song.

Oscine passerines are vocal learners, acquiring their songs through exposure to tutors during a sensitive period early in life. So far, the only description of rhythm ontogeny in a songbird (zebra finches (*Taeniopygia guttata*)) shows that individual tutees tend to prioritize either rhythmic or spectral precision [22]. Modifications to tutor song can be guided by mate‐choice pressure favoring more attractive songs [70], so that the overall song structure may become more attractive across generations. This aligns with the view of vocal learning not as a unitary trait but as a composition of partially independent modules (vocal versatility, coordination, and production variability [71]), where rhythmic entrainment and vocal imitation can be at least partially independent. This learning mechanism creates the necessary conditions for culturally transmitted variation, with individuals copying and potentially modifying patterns of their tutors in both rhythm and frequency, introducing idiosyncrasies that lead to individual differences but also accumulate across generations. Cumulative cultural evolution is known to guide long‐term changes in birdsong structure through transmission of note and syllable type as well as their sequential organization [72]. Similar mechanisms likely guide the evolution of birdsong rhythm, accumulating changes introduced by imperfect copying, adjustments for mate choice, and personal modifications.

Within populations, rhythmic diversification can, for example, increase overall vocal similarities between neighbors [5], improving individual recognition. Acoustic diversification between populations can serve as a pre‐mating barrier in early population divergence [73]. In time, rhythmic differentiation might appear as an early signal of speciation. This would be best tested in clearly separated populations, such as nonmigratory insular species.

### 4.5 Future Directions

While we were purely interested in the rhythmic aspect of tawny pipit songs in this study, we suggest that future research on this and other species should take the full picture (i.e., both spectral and a comprehensive set of rhythmic parameters) into account. Note that here, we have decided to use single values or summary statistics for all parameters for each song (so that, e.g., integer ratios are presented as minimum, maximum, and mean), following one of standard approaches in bioacoustics. Using the full set of integer ratios of all element pairs might provide a level of detail similar to musical notation and possibly prove useful in further research. The rhythmic signal structure of woodpeckers innate drumming behavior shows clear phylogenetic differences [74], indicating at gene‐driven evolution of its rhythm. Similarly, pulse rate and rhythmic stability of the vocally nonlearning tinkerbirds' (*Pogoniulus*) isochronous songs is genetically driven [75]. How might rhythms develop in vocal learners, such as pipits? A future study, pooling data from more congeneric species sampled across their distribution, and adding a genetic dimension (as to quantify population divergence and connectivity), should help us disentangle phylogenetic and cultural impacts on song evolution. Whether driven by genes or memes (small cultural units that spread by imitation) [76, 77], if evolution of rhythm occurs, similar patterns might emerge in various populations (or, in fact, be conserved across populations, similarly to human music [78, 79]).

Both individual‐ and population‐scale rhythmic patterns emerge clearly in the species' song, reinforcing rhythm as an important dimension of avian communication [5, 29, 80]. Whether and how birds themselves perceive and use this information, however, remains unclear [81, 82]. Dedicated playback experiments based on artificial songs with modified rhythmic parameters would be of help here. We suggest, however, to first address this issue in a species with more organized, isochronous songs, where such measures are easier to interpret and modify. Some bird species use and spontaneously entrain to rhythmic patterns (e.g., Refs. [22, 83, 84]), and songbird vocal athletics (in terms of frequency precision and tempo) are an important part of mating systems in this group [85, 86]. In the vocally nonlearning tinkerbird species, both higher pulse rate and rhythmic stability seem to preferred by *P. pusillus pusillus*, potentially limiting hybridization with the sister species *P. chrysoconus extoni* [75]. We suggest that parameters such as beat precision might also signal the singer's quality (similar to how professional musicians produce more exact beats than a layperson).

Although not considering the usefulness of rhythm characteristics to birds themselves, this work provides new and useful insights into their communication systems. Understanding that both identity and population information can be contained within the rhythmic structure can also contribute to improving passive acoustic monitoring of animal populations [9]. This is also a step forward in understanding the origins and function of rhythm, and the evolution of music and communication [41, 43, 44, 45].

## 5 Conclusions

We present the first evidence that modern rhythm analysis methods can reliably describe arrhythmic sequences. The case study of the European tawny pipit population shows that rhythmic differences in such arrhythmic songs can be used to identify both individual singers and their population of origin, the latter previously not identified acoustically with success.

## Author Contributions

**Adrián Barrero**: investigation. **Anna N. Osiecka**: conceptualization, methodology, investigation, data curation, writing – original draft, writing – review and editing, project administration, software, visualization, funding. **Adam Petrusek**: methodology, investigation, visualization, writing – review and editing. **Cristian Pérez‐Granados**: investigation, writing – review and editing. **Javier Oñate‐Casado**: methodology, investigation, data curation, writing – review and editing, funding. **Juan Traba**: investigation, funding, writing – review and editing. **Lara S. Burchardt**: methodology, software, writing – review and editing, supervision. **Michal Porteš**: investigation. **Tereza Kubíková**: investigation. **Tereza Petrusková**: methodology, investigation, writing – review and editing, funding. **Václav Beran**: investigation, funding.

## Funding

A.N.O. was supported by Humboldt Foundation Postdoctoral Fellowship. L.S.B. was supported by German Research Council grant DFG BU4375/1‐1, project number: 528064681. T.P. was supported by Czech Science Foundation, project number: 21‐04023K. J.O.‐C. was supported by Charles University Grant Agency, project number: 400422. V.B. was supported by Technology Agency of the Czech Republic, project number: TA04021269. J.T. was supported by LIFE Connect Ricoti: LIFE20‐NAT/ES/000133, Comunidad de Madrid, Excellence Network Remedinal TE: S2018/EMT‐4338.

## Conflicts of Interest

The authors declare no conflicts of interest.

## Supporting information


**Supplementary Material**: nyas70452‐sup‐0001‐SuppMat.pdf

**Figure S1**: nyas70452‐sup‐0001‐FigureS1.png

## Data Availability

All data and code produced for this work are openly accessible at https://osf.io/h2fpm/overview?view_only=60916d5355de494295a1e9612ca3d40c.
