# Supplementary material for: Unique Rhythmic Signatures in Arrhythmic Birdsong
Source: Ann N Y Acad Sci. 2026 Nov 13;1565(1):e70452. doi: 10.1111/nyas.70452 (PMC13762373; doi:10.1111/nyas.70452)

Supplementary Material 1.
Osiecka et al. Unique rhythmic patterns in arrhythmic birdsong

Inter-annotator agreement

While all annotations used in this study were prepared by a single annotator (JO-C), we present an additional inter-annotator agreement assessment to assess annotation robustness. This comparison uses a subset of data (54 songs sampled from across the 6 study populations: 3 songs per individual, 3 individuals per population) additionally annotated by an external annotator skilled in Luscinia, but without prior experience with tawny pipit song (MS).

Agreement was assessed by Spearman correlation (function *cor.test, stats* package). Overall, annotators showed good to excellent agreement (Supplementary Table 1)

Supplementary Table 1. Inter-annotator agreement

Parameter	Spearman rho	Agreement
Start time	0.999	excellent
Duration	0.726	good
Element number per song	0.773	very good
IOI	0.973	excellent

1. **Start time.** Annotators agreed nearly perfectly. Spearman rho falls within excellent annotator agreement.

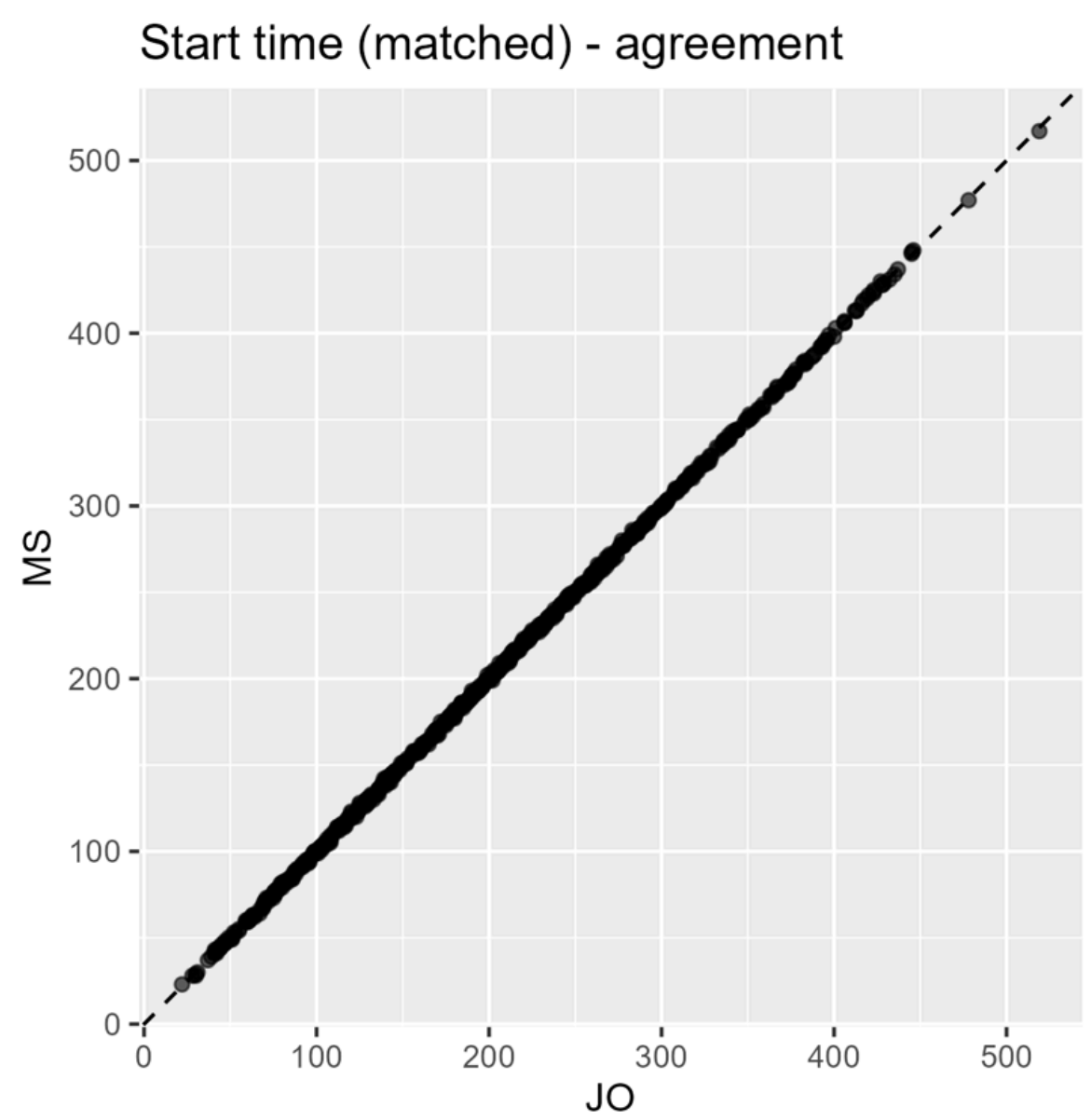

2. **Duration (length).** Annotators tended to distinguish elements differently, and both no. of elements and element durations (lenghts) showed the biggest disagreements. Spearman rho = 0.726 still falls within good annotator agreement.

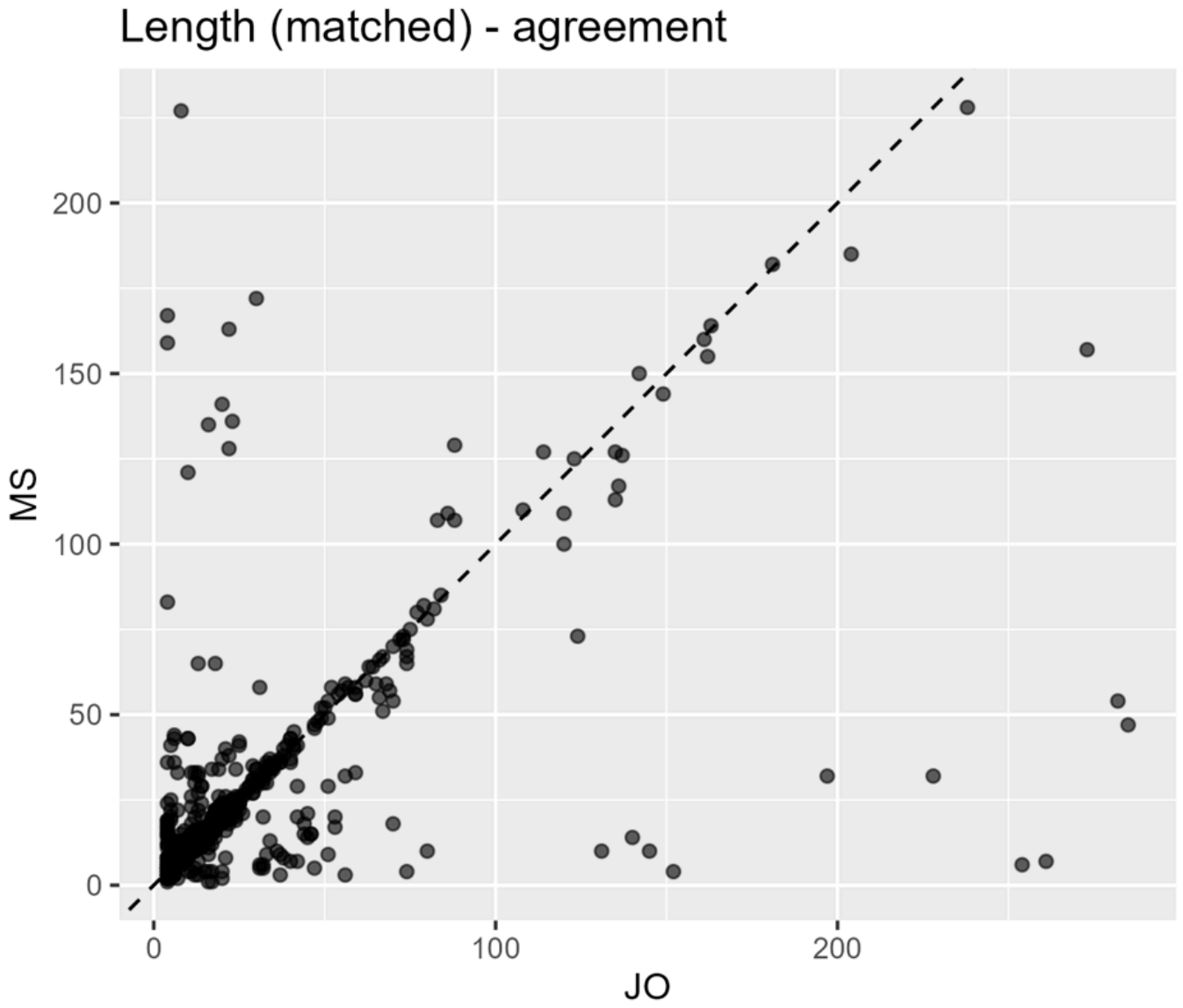

3. **Elements per song.** There is a small difference in how many elements are marked as separate by each annotator. Spearman rho = 0.773 falls within very good inter-annotator agreement.

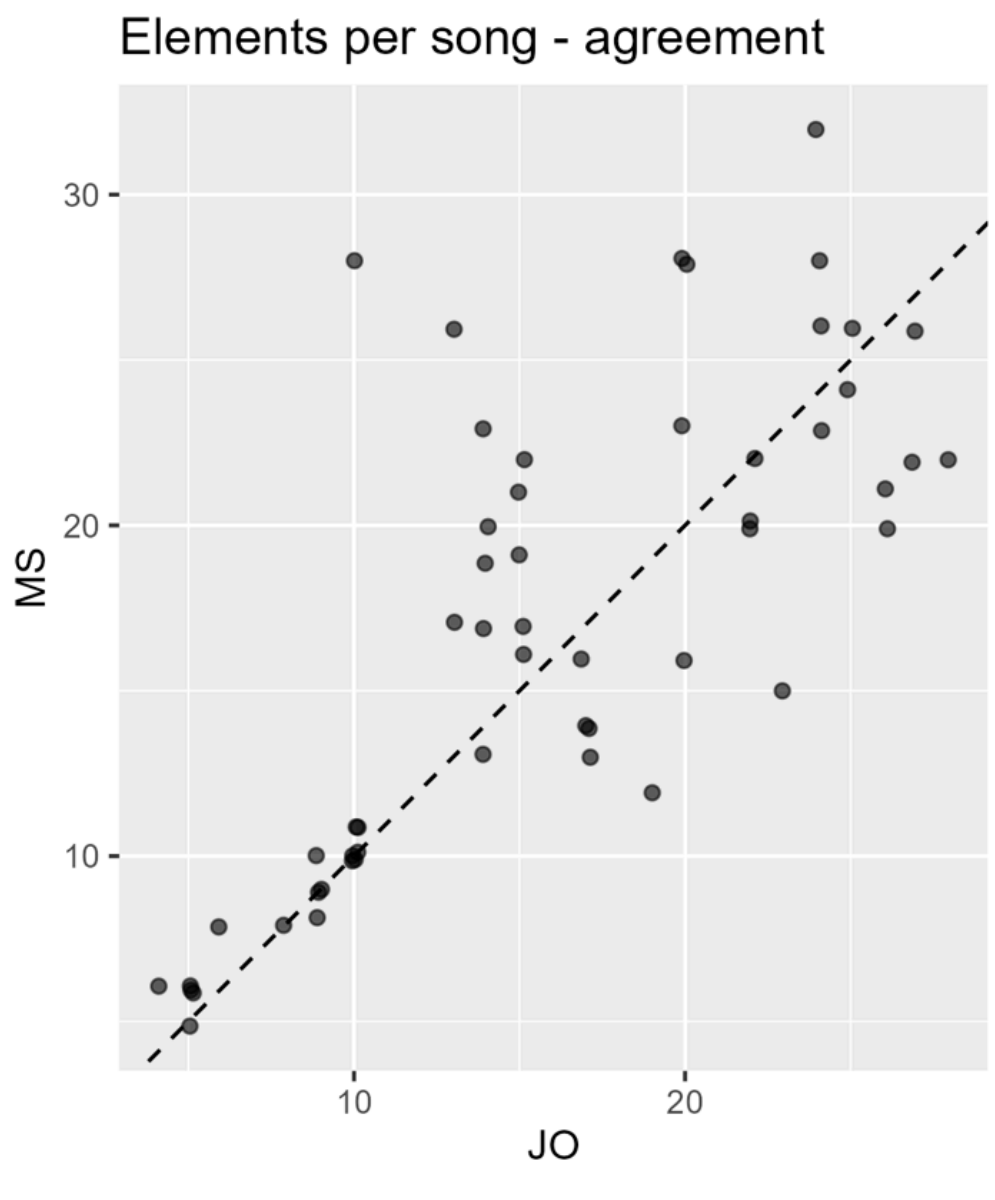

4. **Inter-onset-intervals (IOI).** As can be expected from start time agreement, IOIs showed excellent annotator agreement (Spearman rho = 0.973).

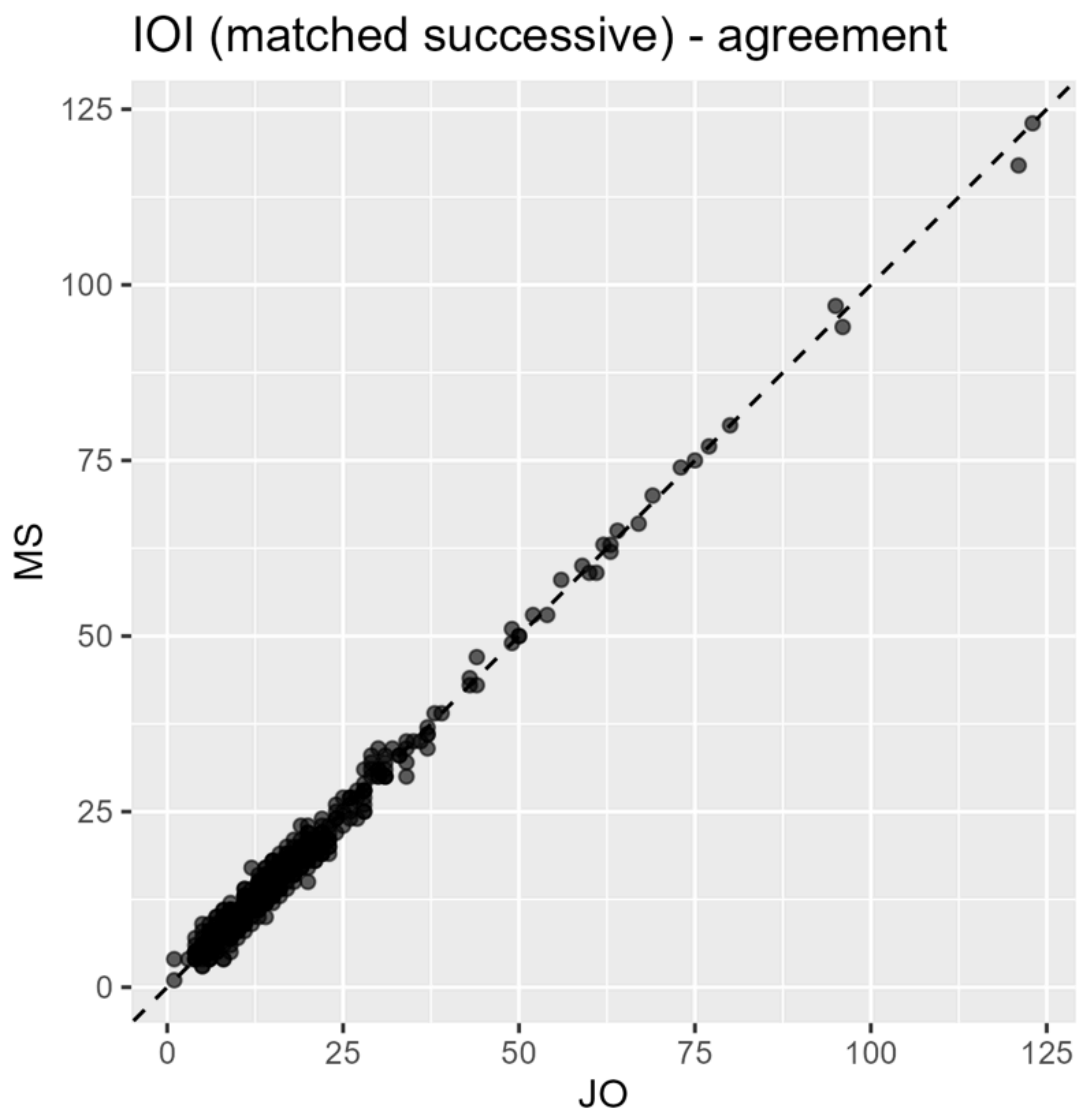

Spectral settings impact on visible song structure

All annotations used in this study relied on the same spectral settings. Below, to visualise how chosen spectral settings might affect annotation decisions, we present a subset of songs (the same as used for the inter-annotator agreement checks) plotted in 6 different settings: (1) study settings, (2) settings used for the clean illustrative figures in the paper, (3) lowered resolution: FFT, (4) lowered resolution: step, (5) lowered resolution: window, and (6) lower resolution: df.

Note that these spectra are an illustrative example, and do not represent annotation quality (see Supplementary Figure 1 for examples of Luscinia-generated, annotated spectra).

GS_76.20/76.20 ACGS_ 1632_ed_2.wav

sampling rate 44100 Hz, 520 ms

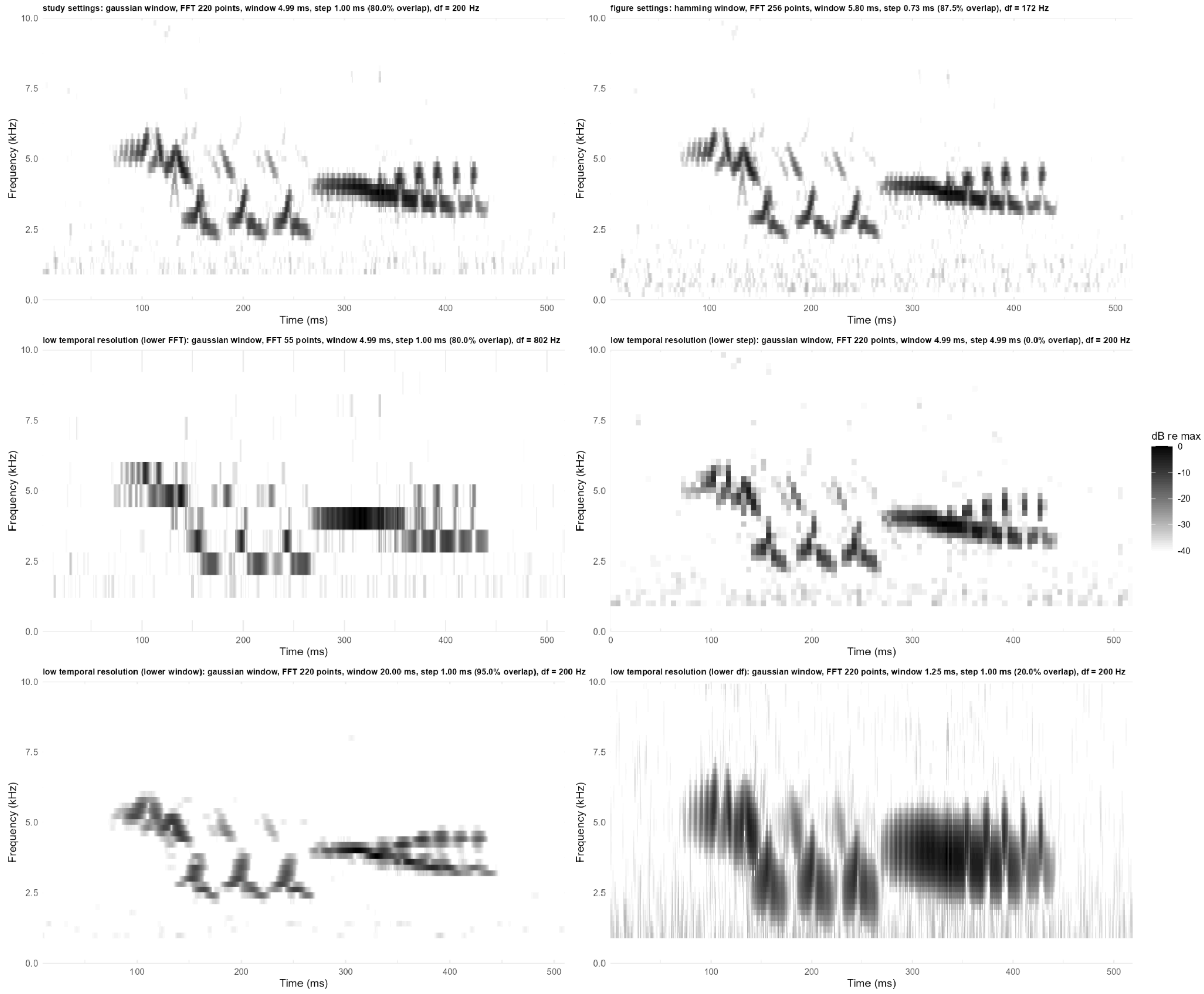

GS_76.20/76.20 ACGS_ 1632_ed_3.wav
sampling rate 44100 Hz, 501 ms

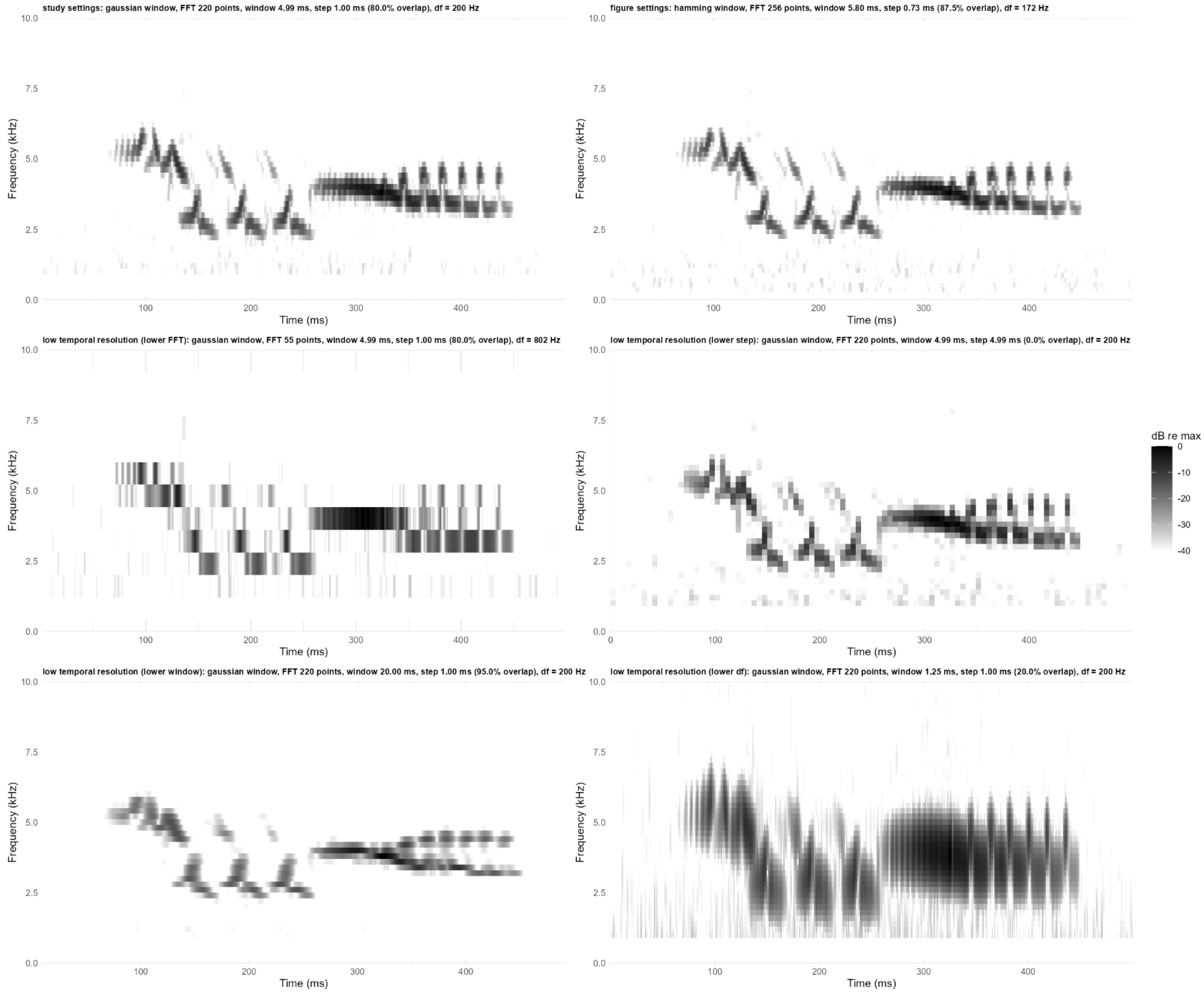

MP_02.23 ACMP/KUBI055 20230525.WAV_1.wav

sampling rate 44100 Hz, 732 ms

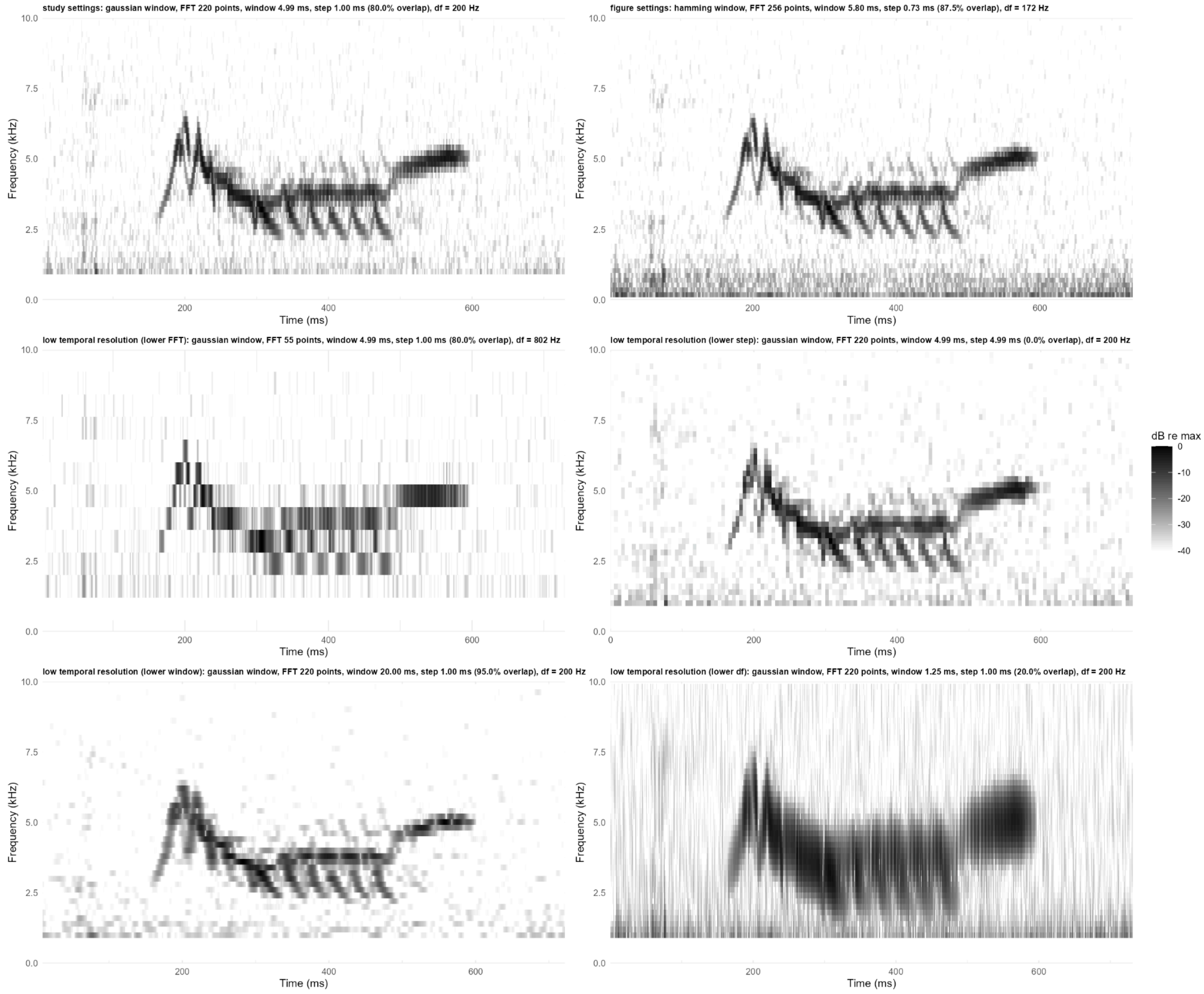

MP_02.23 ACMP/KUBI055 20230525.WAV_2.wav

sampling rate 44100 Hz, 638 ms

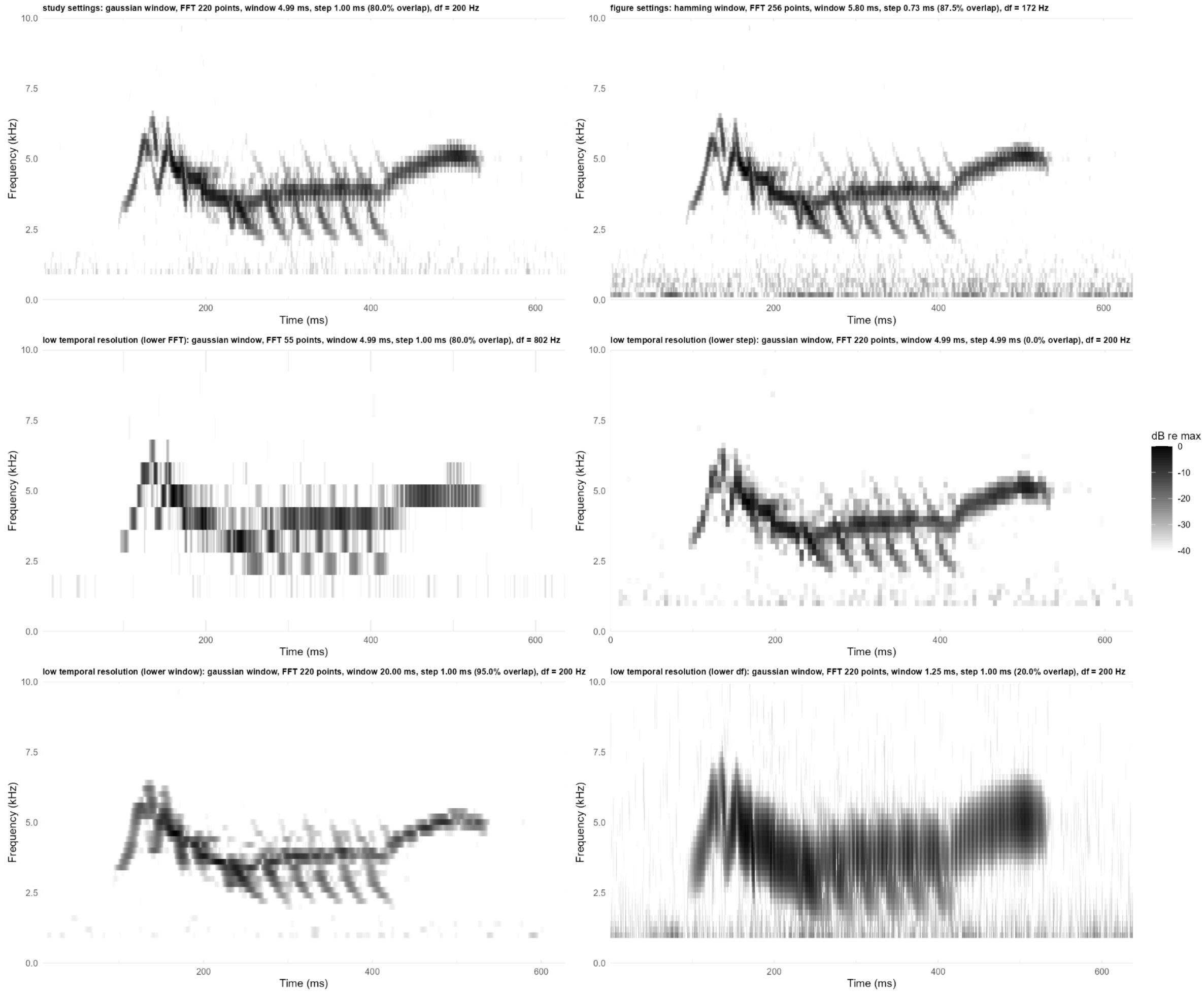

MP_02.23 ACMP/KUBI055 20230525.WAV_3.wav

sampling rate 44100 Hz, 727 ms

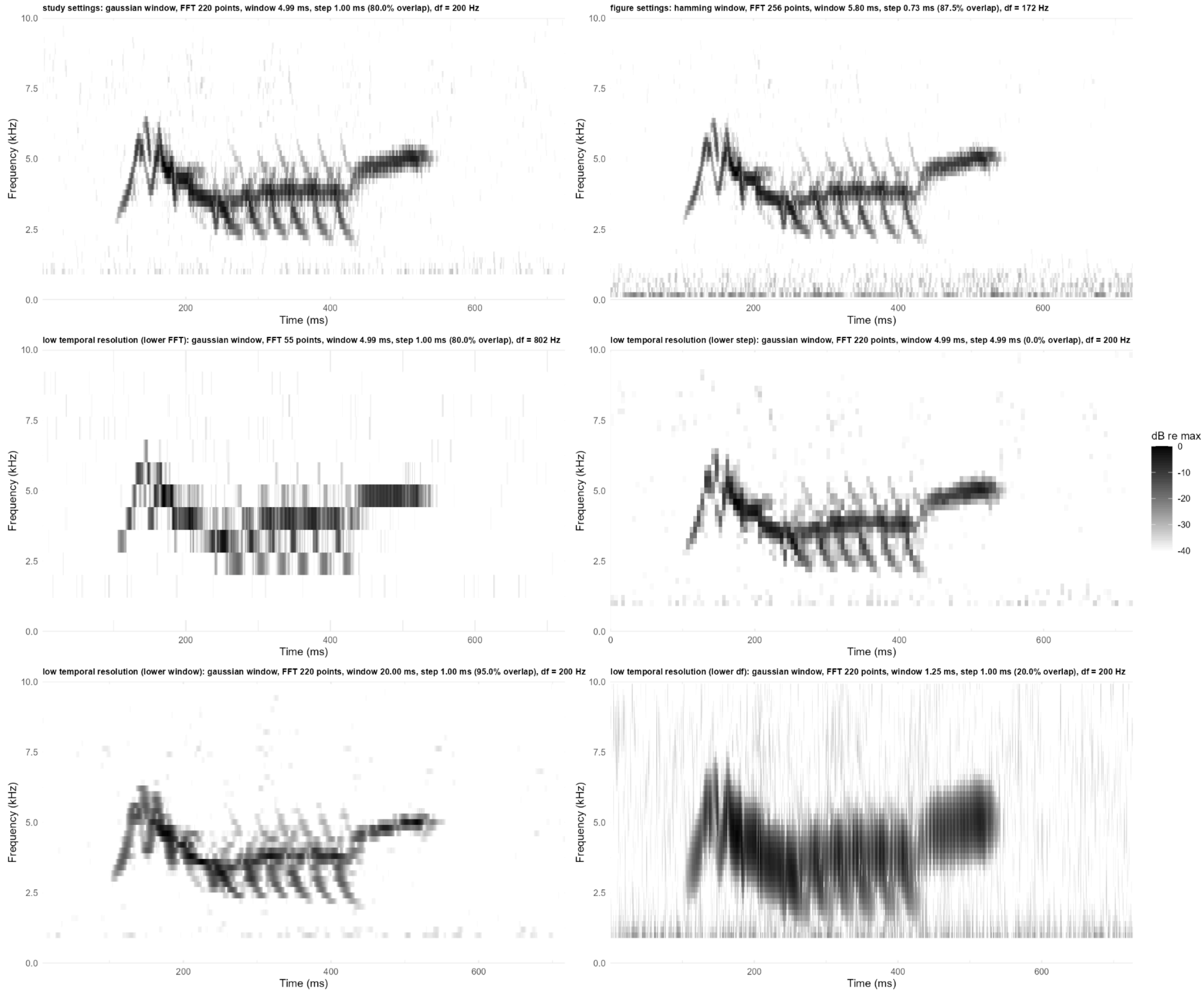

MP_04.23 ACMP/KUBI061 20230525.WAV_1.wav

sampling rate 44100 Hz, 528 ms

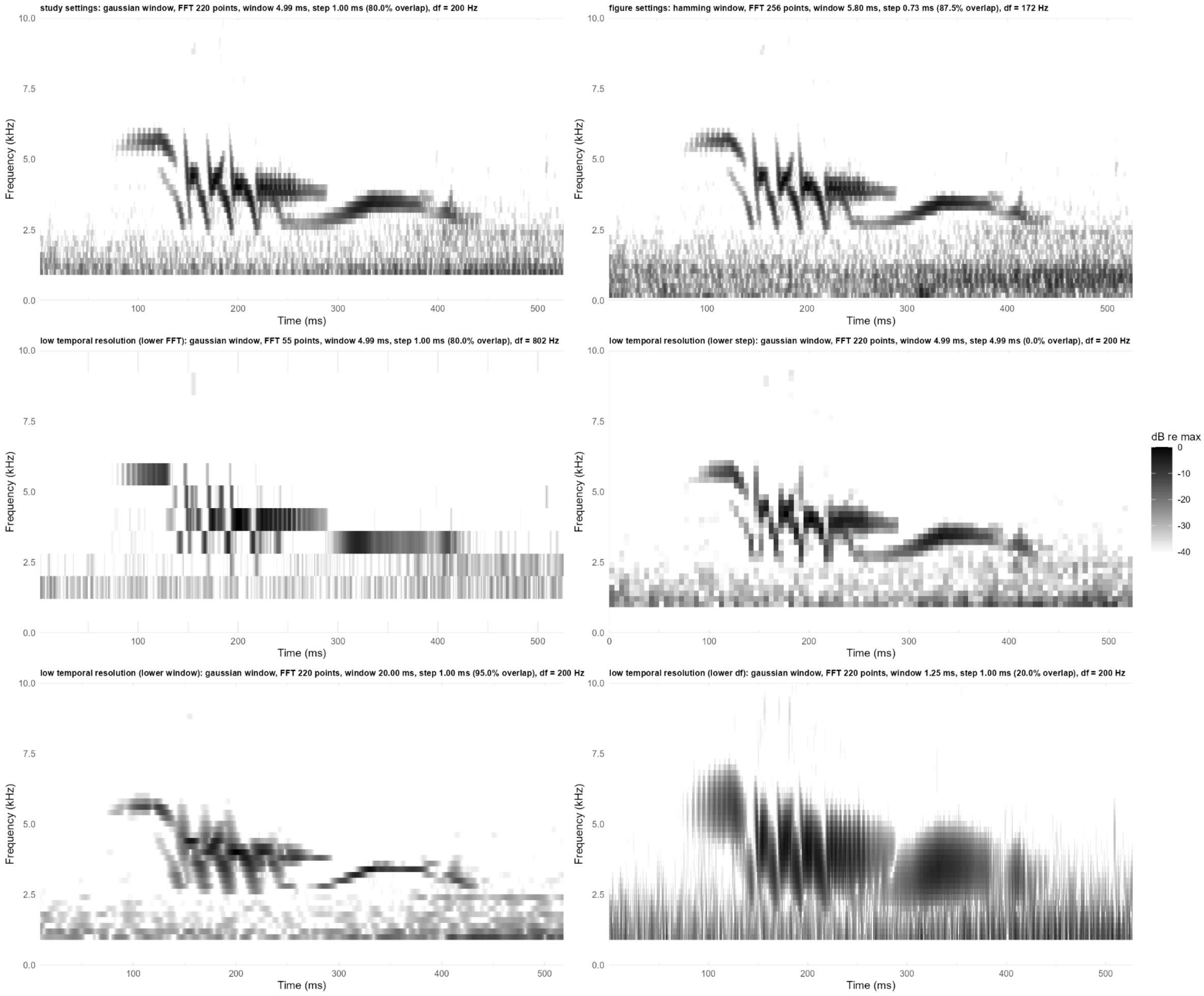

MP_04.23 ACMP/KUBI061 20230525.WAV_2.wav

sampling rate 44100 Hz, 517 ms

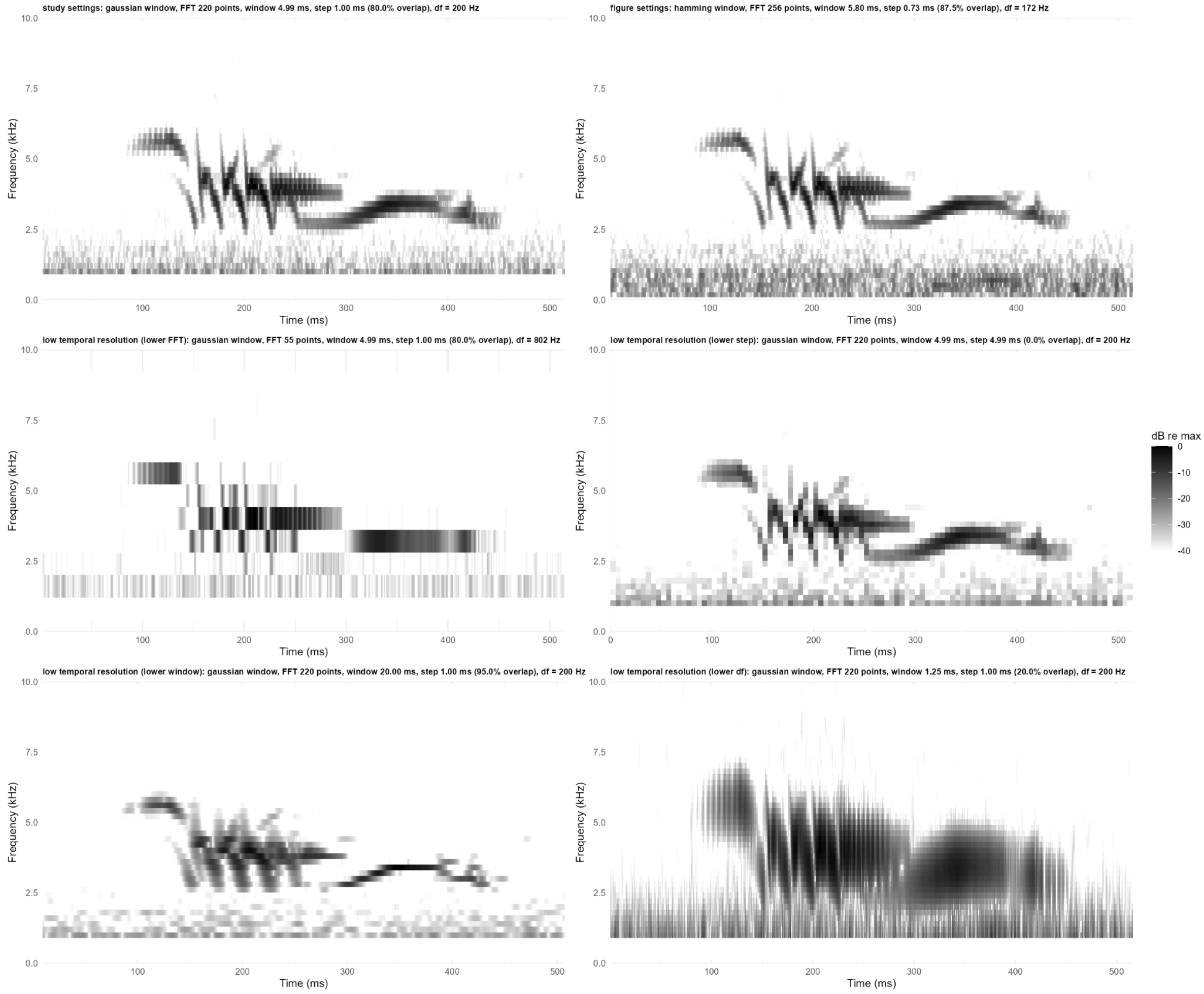

MP_04.23 ACMP/KUBI061 20230525.WAV_3.wav

sampling rate 44100 Hz, 510 ms

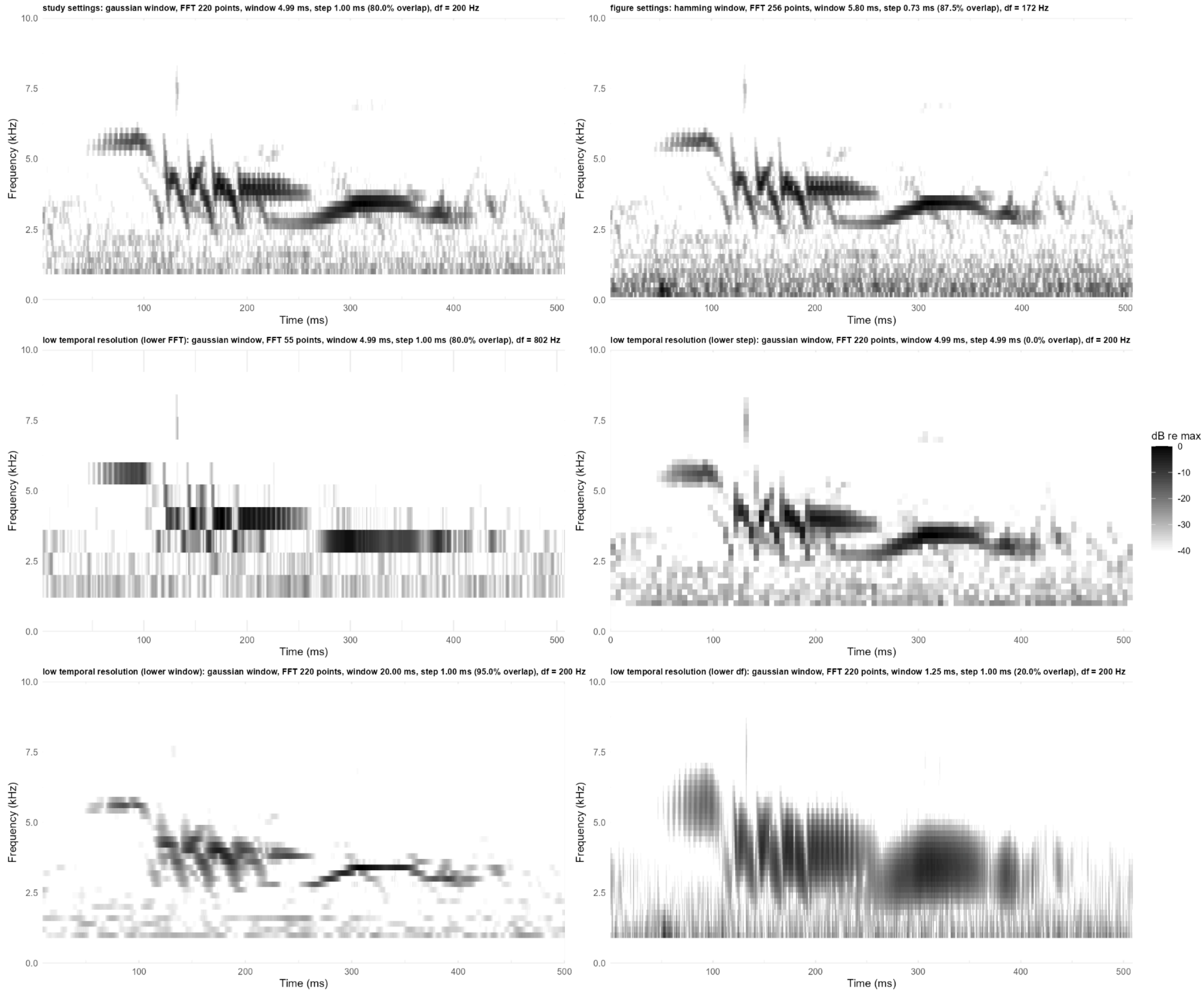

MP_18.23 ACMP/KUBI098 20230528.WAV_1.wav

sampling rate 44100 Hz, 474 ms

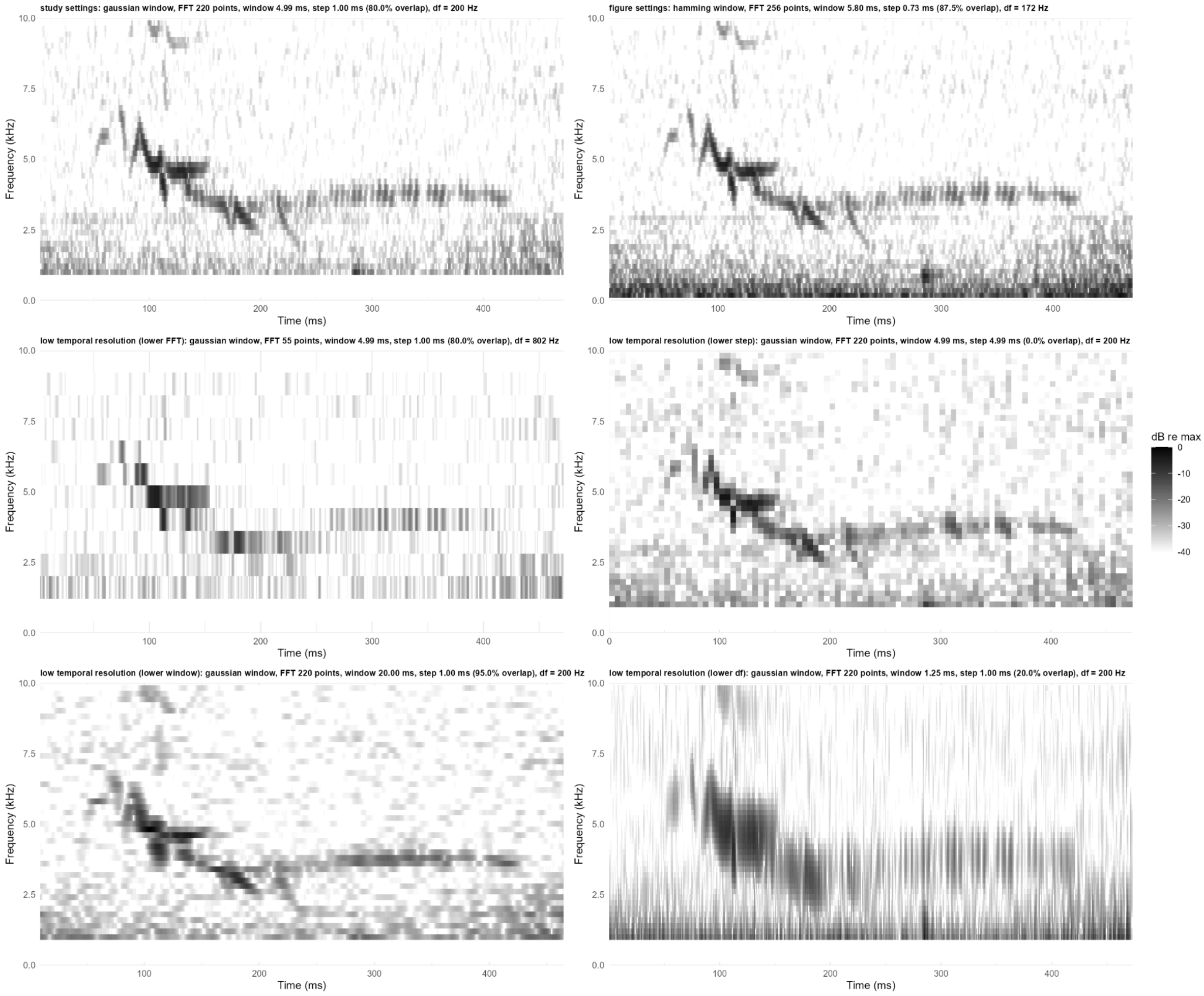

MP_18.23 ACMP/KUBI098 20230528.WAV_2.wav

sampling rate 44100 Hz, 442 ms

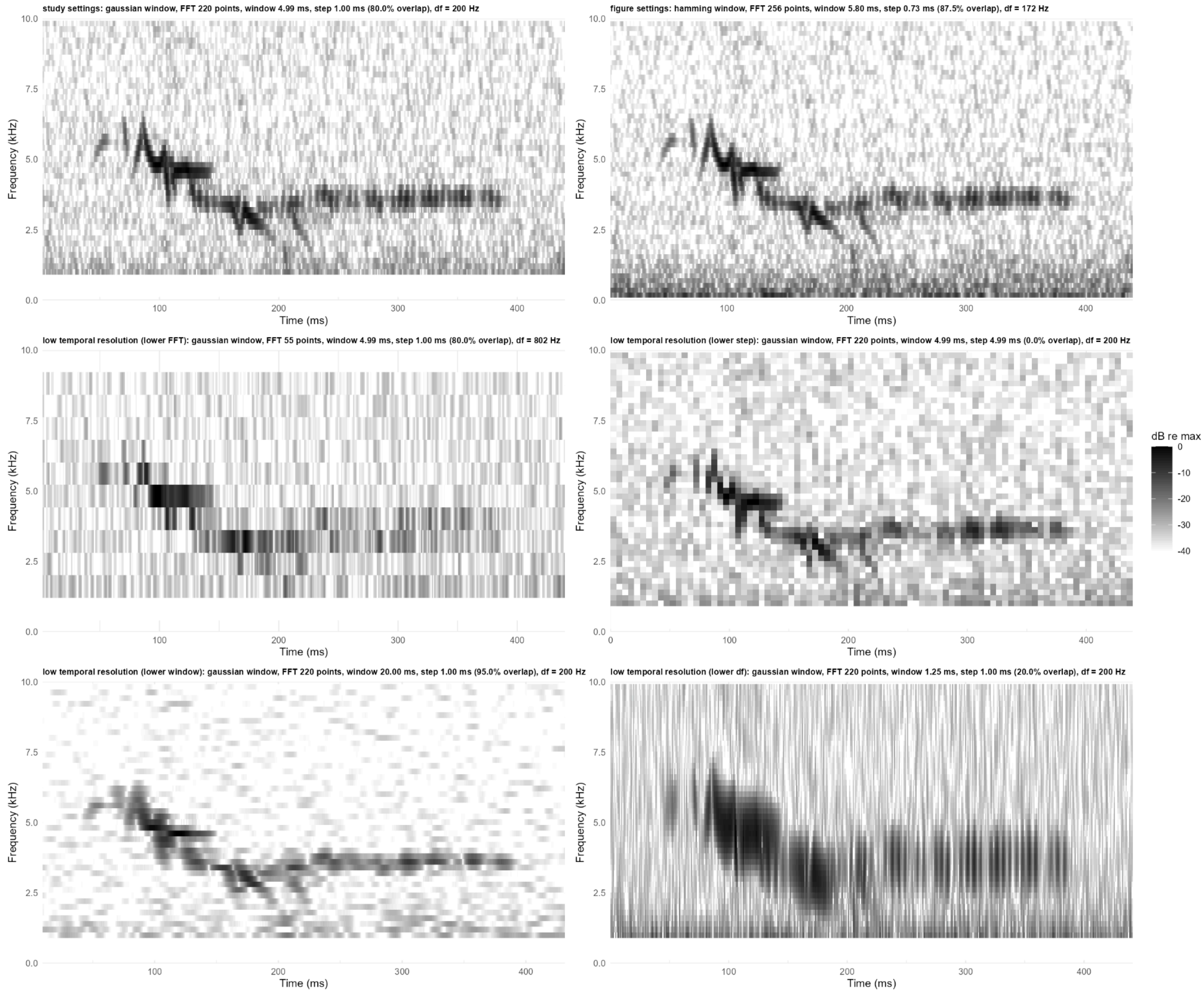

MP_18.23 ACMP/KUBI098 20230528.WAV_3.wav

sampling rate 44100 Hz, 392 ms

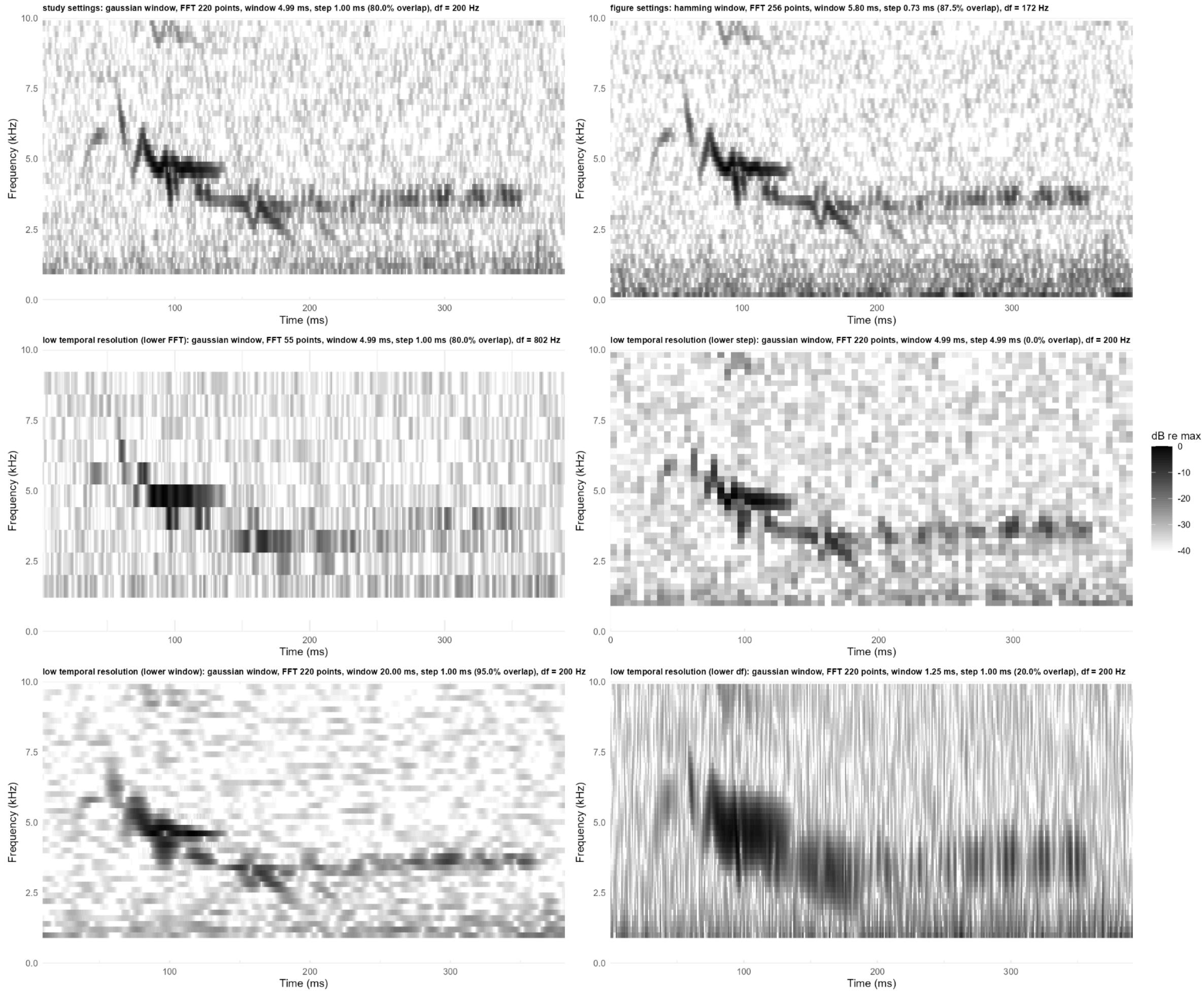

ADM_08.21 ACA_ringed/J465 20210524.WAV_1.wav

sampling rate 44100 Hz, 667 ms

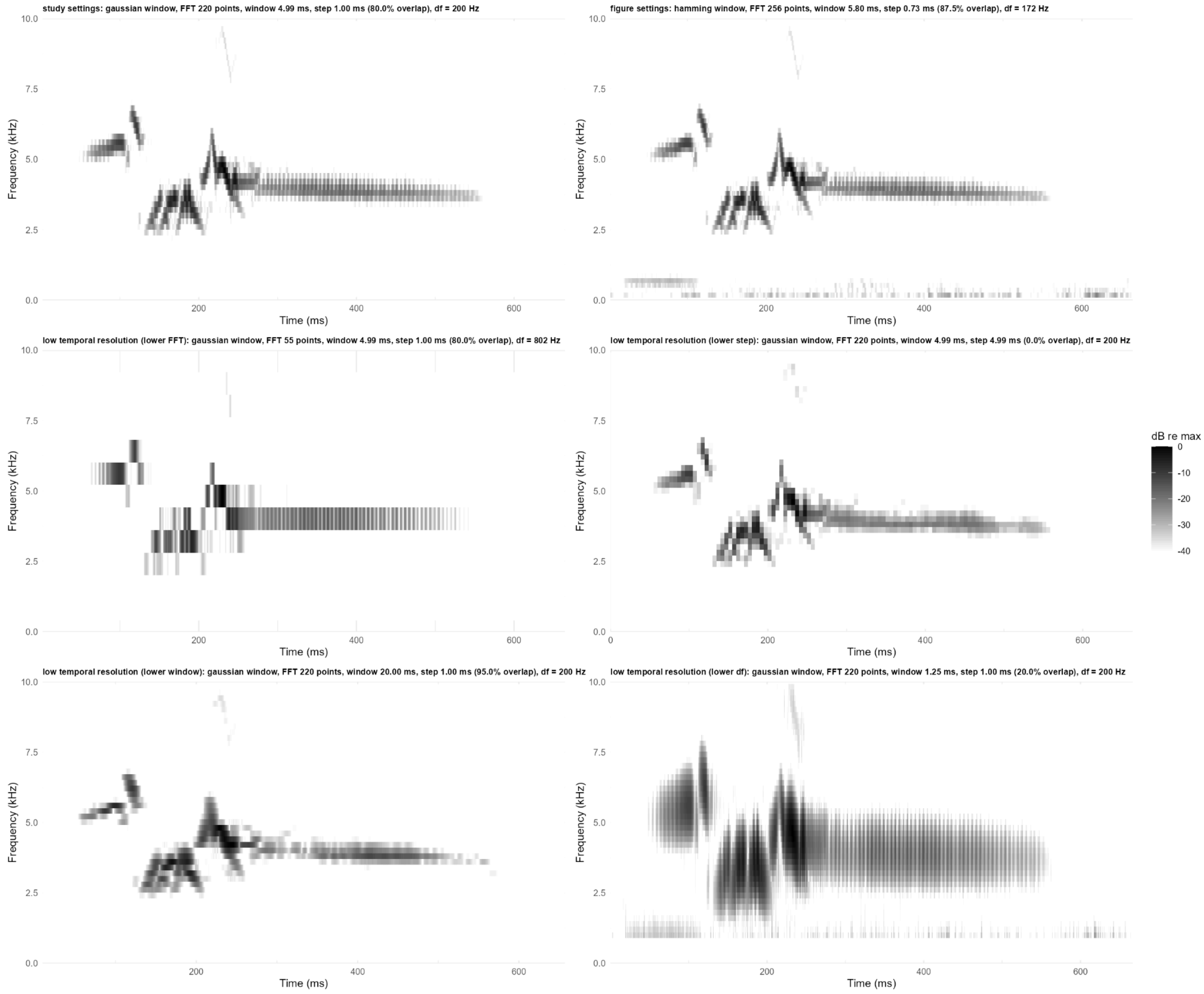

ADM_08.21 ACA_ringed/J465 20210524.WAV_2.wav
sampling rate 44100 Hz, 695 ms

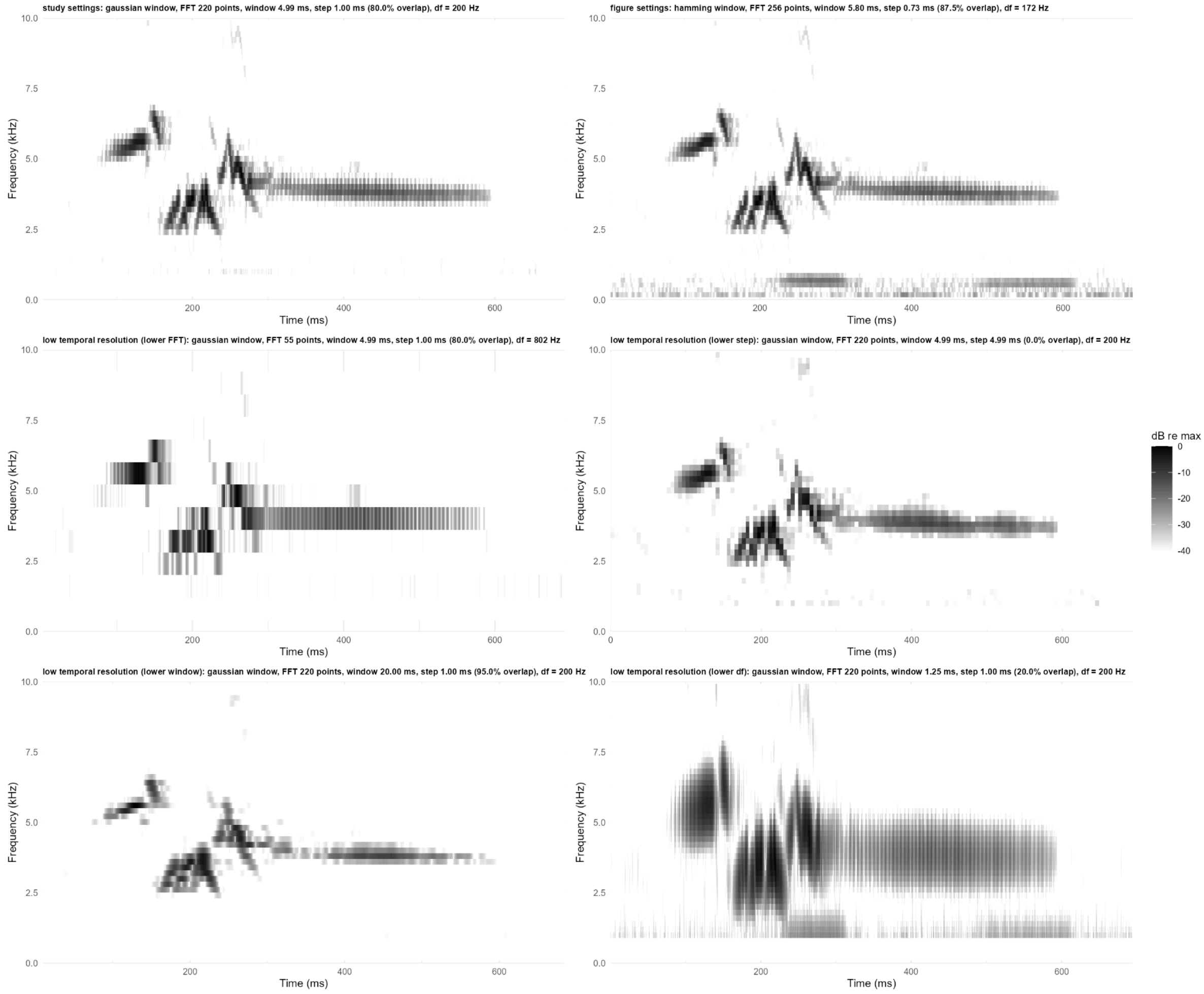

ADM_08.21 ACA_ringed/J465 20210524.WAV_3.wav

sampling rate 44100 Hz, 631 ms

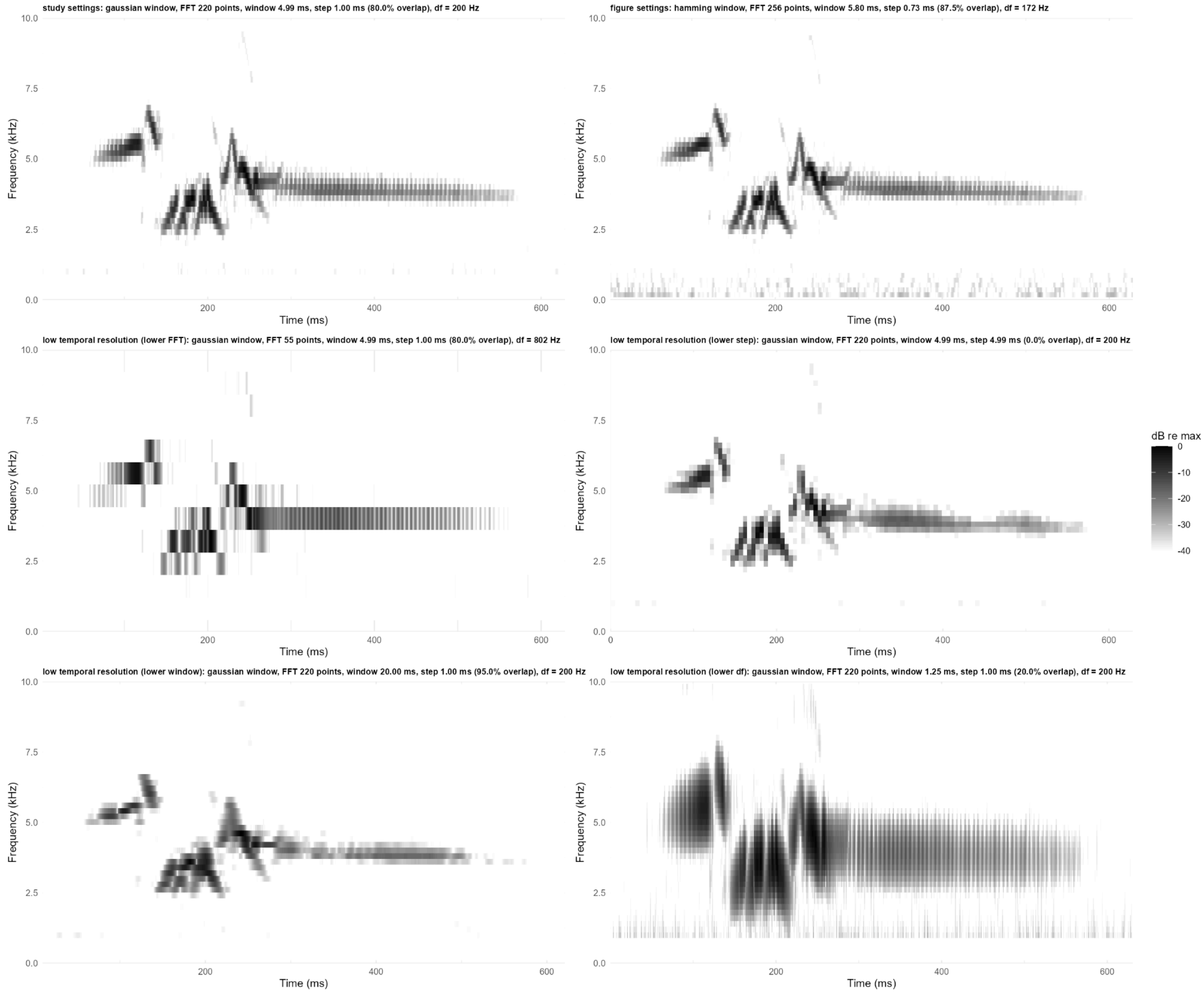

ADM_17.21 ACA_ringed/J503 20210526.WAV_1.wav

sampling rate 44100 Hz, 515 ms

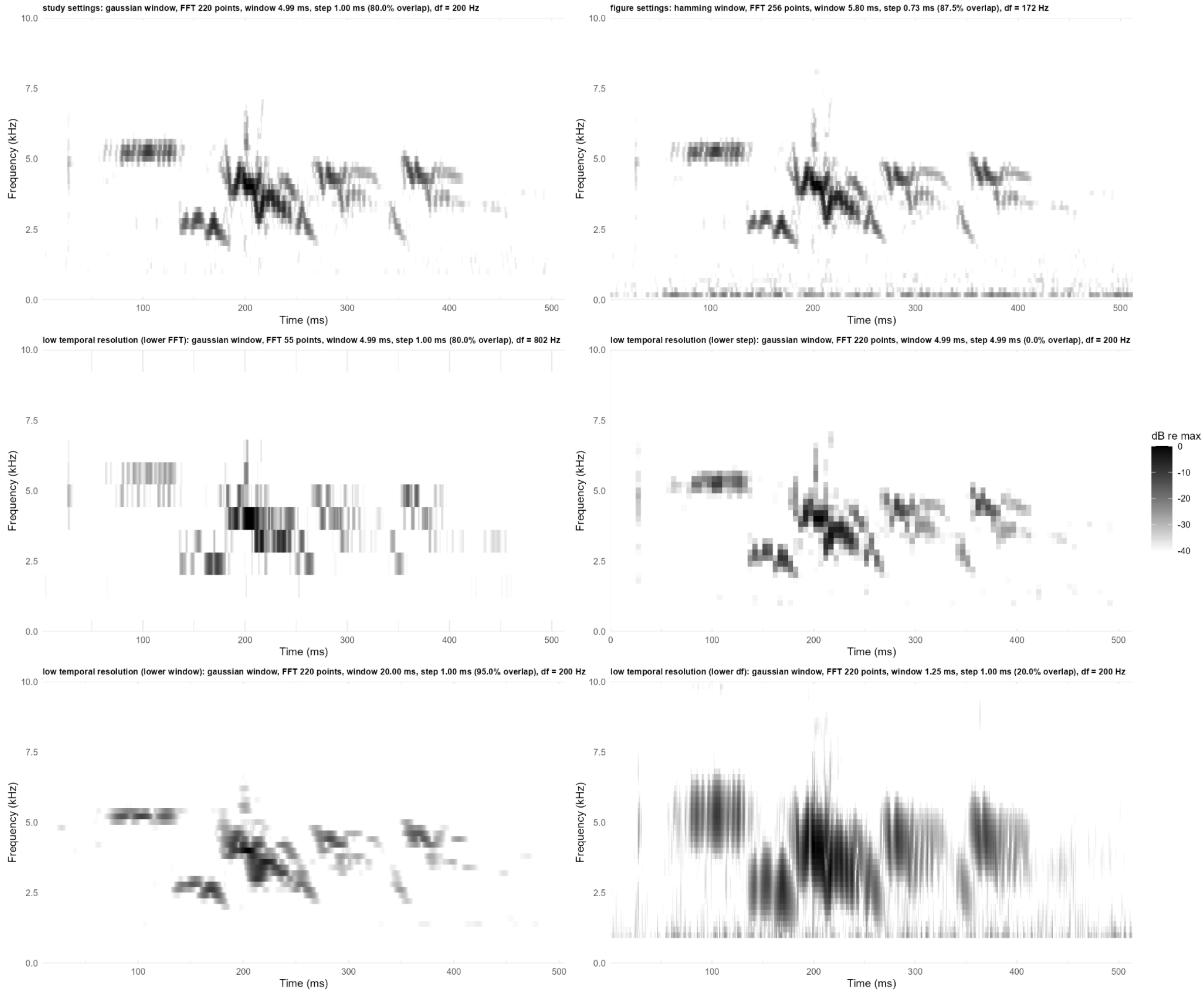

ADM_17.21 ACA_ringed/J503 20210526.WAV_2.wav

sampling rate 44100 Hz, 519 ms

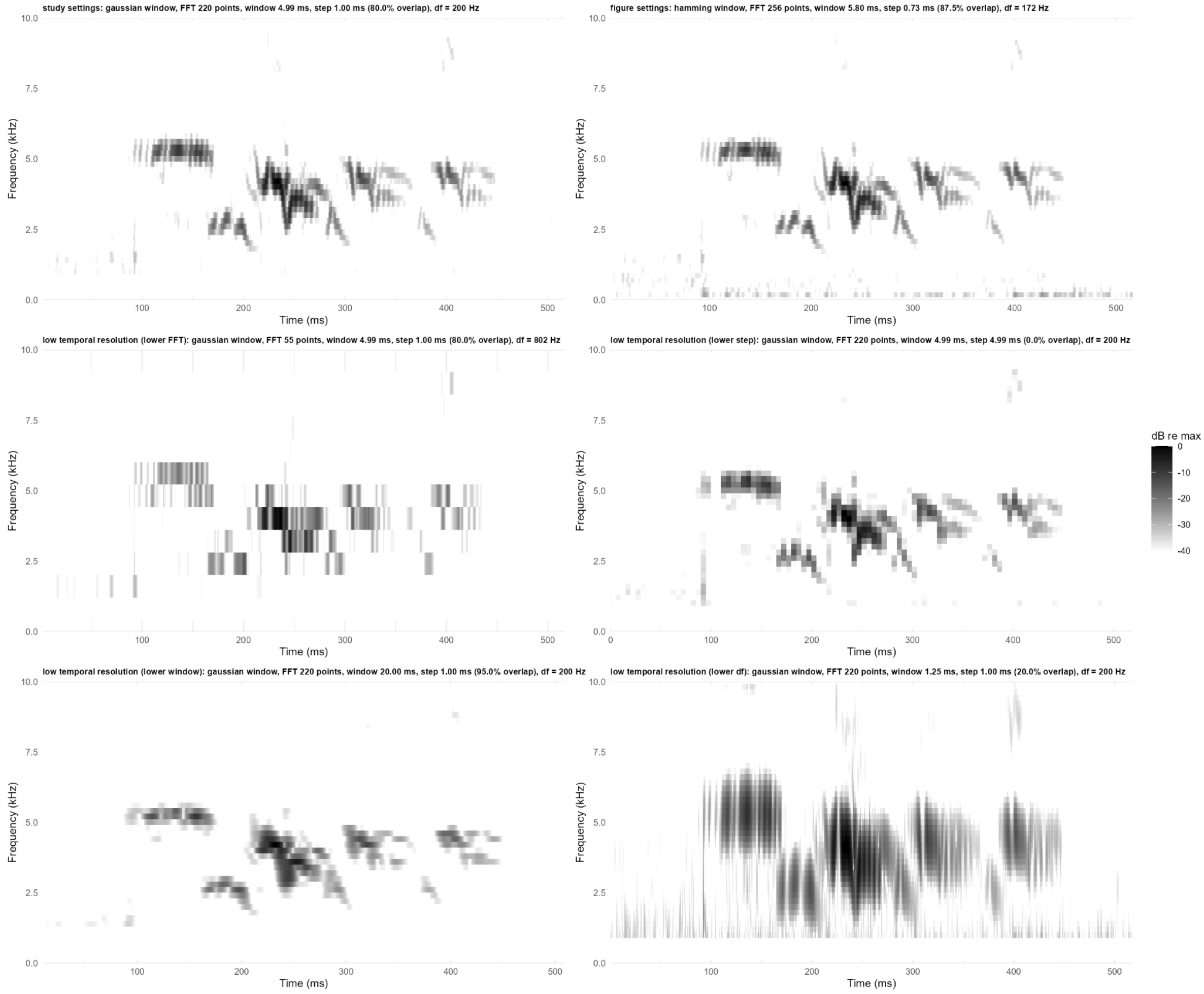

ADM_17.21 ACA_ringed/J503 20210526.WAV_3.wav

sampling rate 44100 Hz, 537 ms

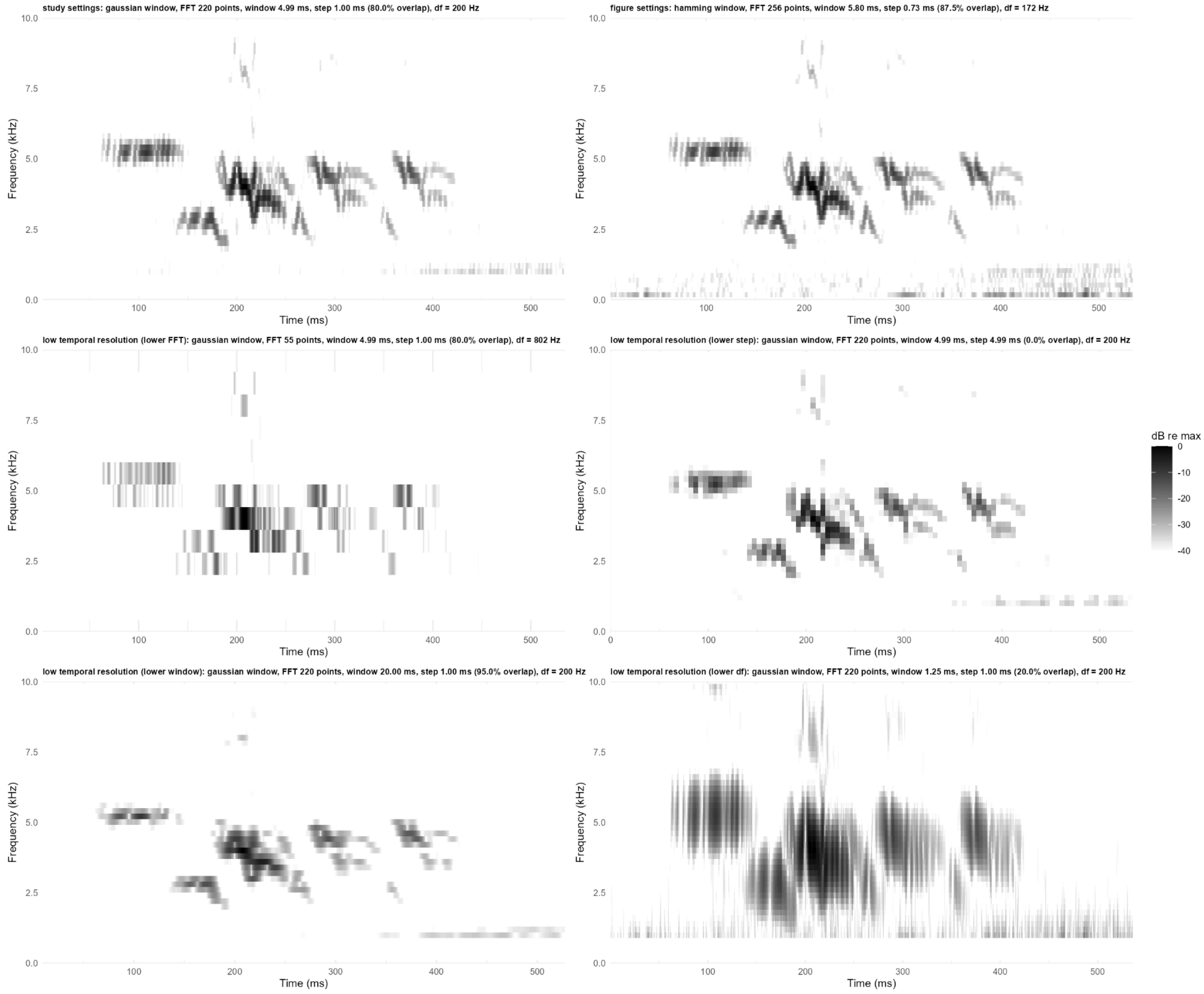

ADM_28.22 ACA/J583 20220522.WAV_1.wav

sampling rate 44100 Hz, 625 ms

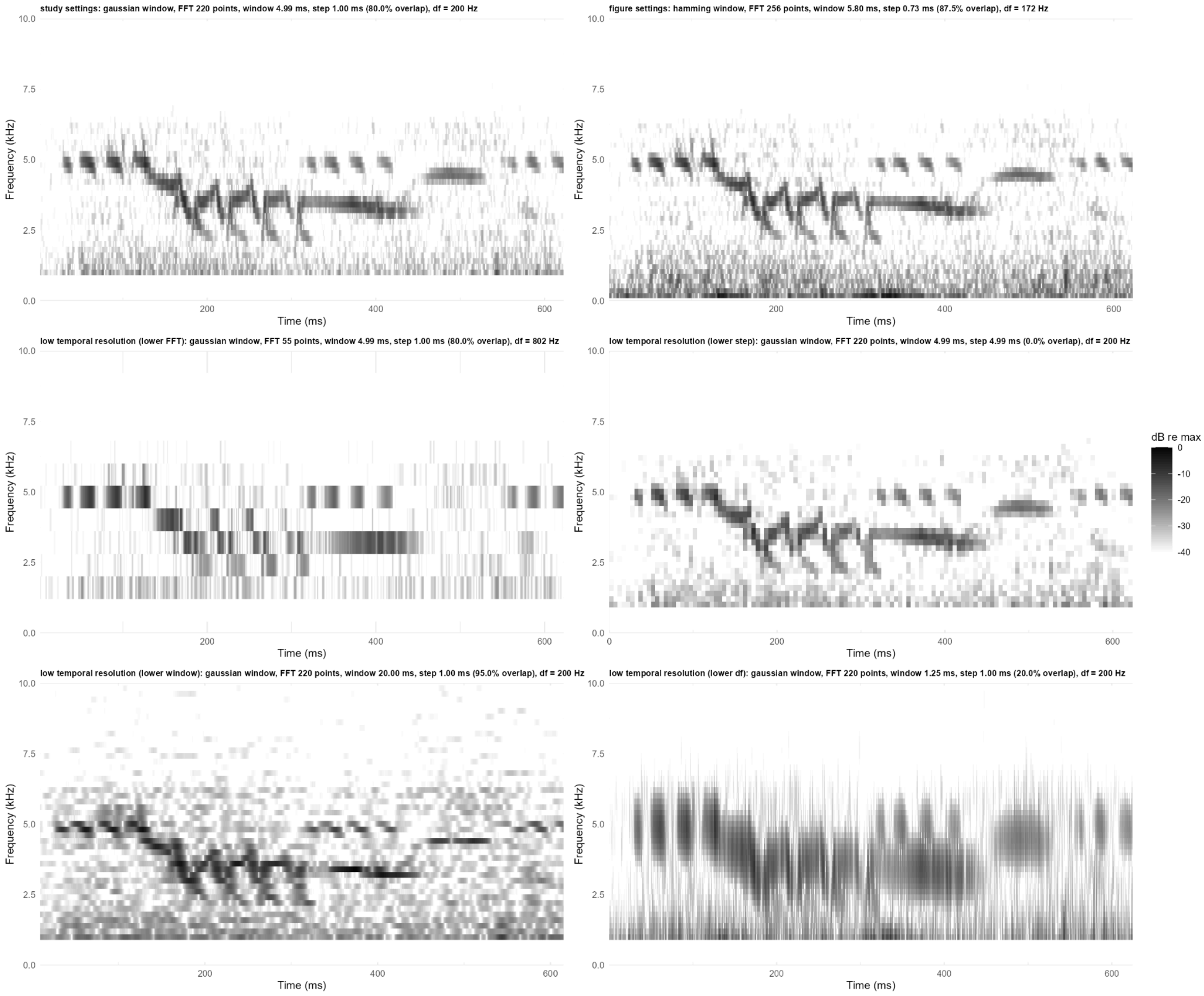

ADM_28.22 ACA/J583 20220522.WAV_2.wav
sampling rate 44100 Hz, 552 ms

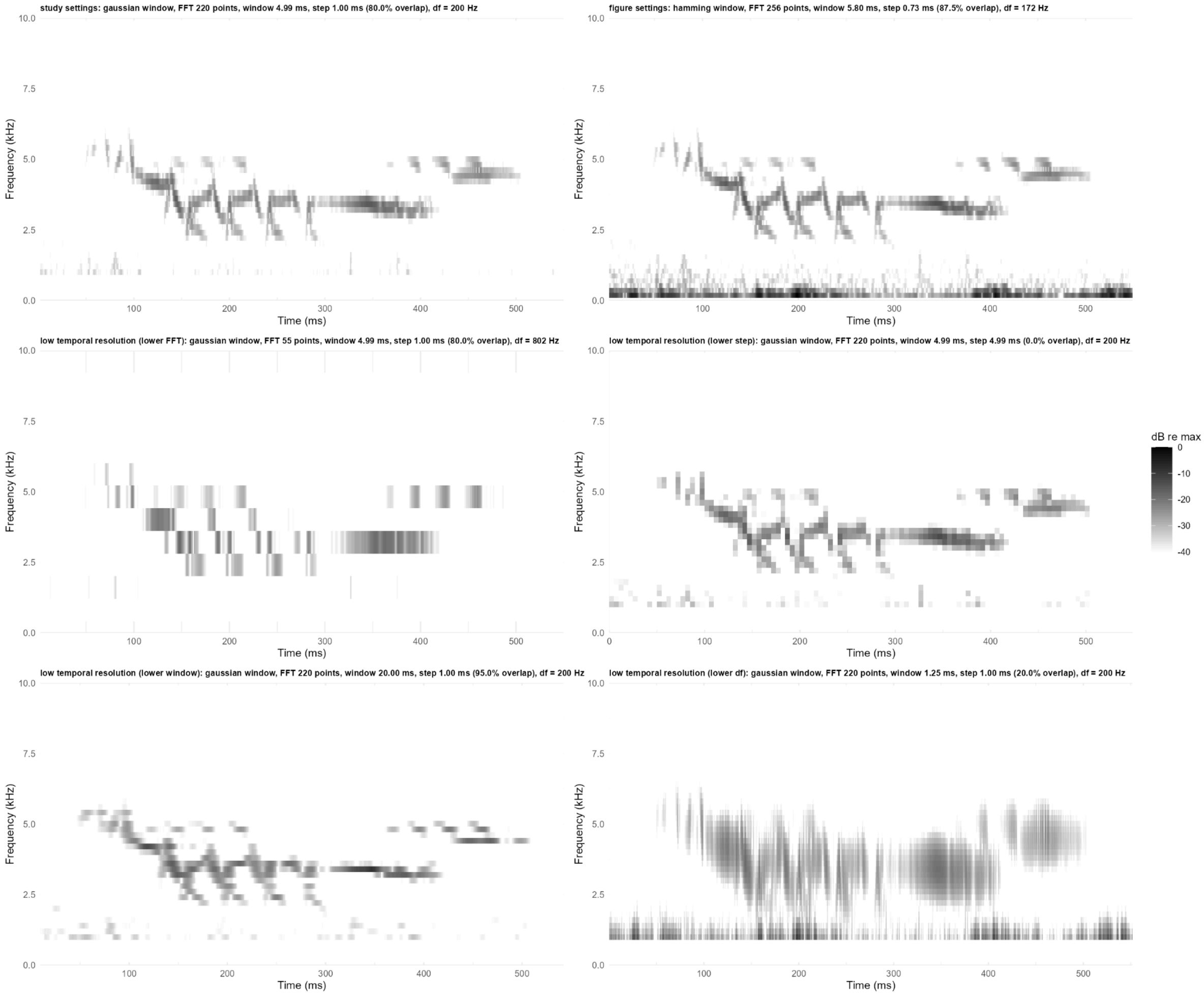

ADM_28.22 ACA/J583 20220522.WAV_3.wav

sampling rate 44100 Hz, 672 ms

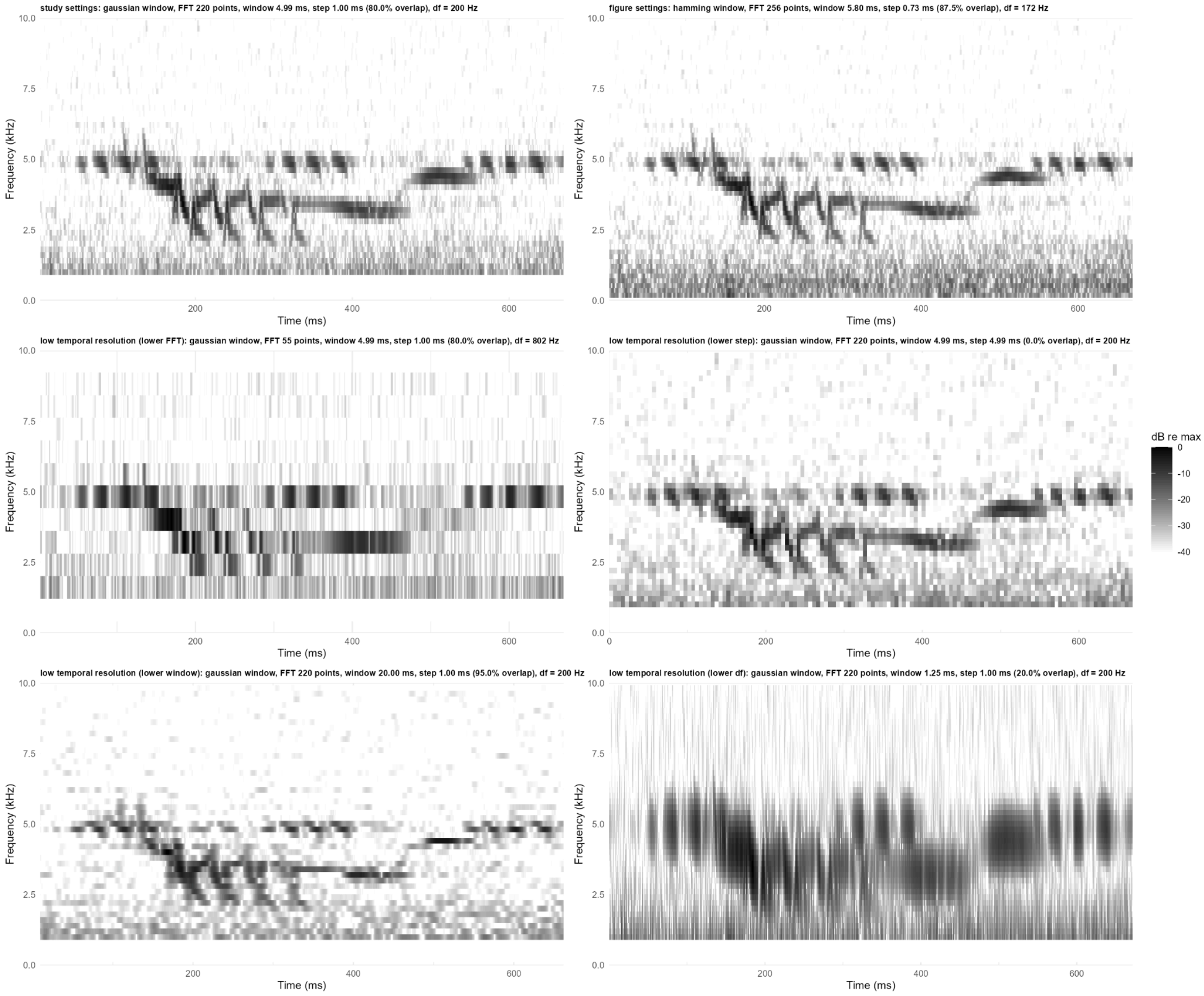

AM_03.23 ACAM/J899 20230519.WAV_1.wav

sampling rate 44100 Hz, 822 ms

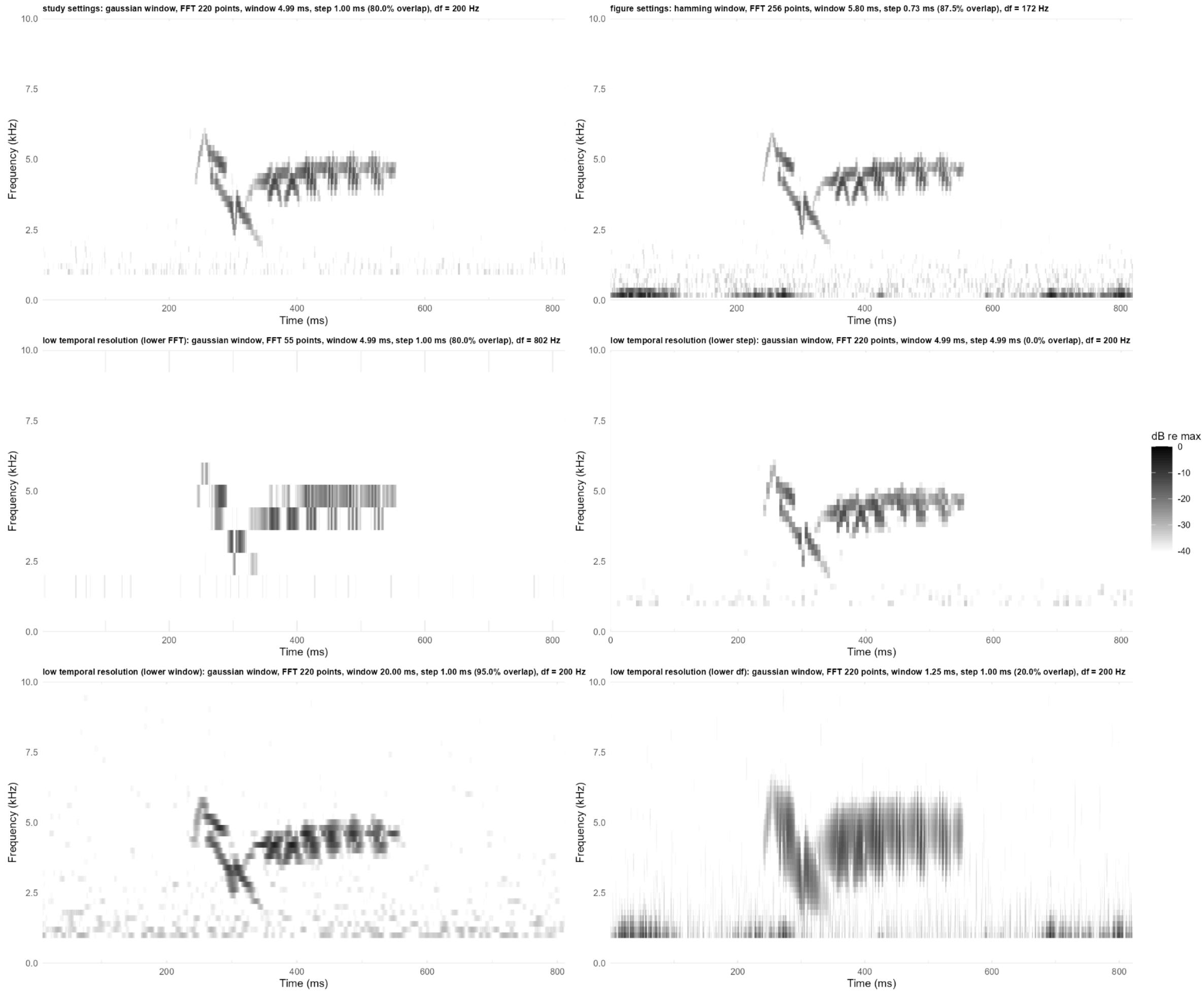

AM_03.23 ACAM/J899 20230519.WAV_2.wav

sampling rate 44100 Hz, 727 ms

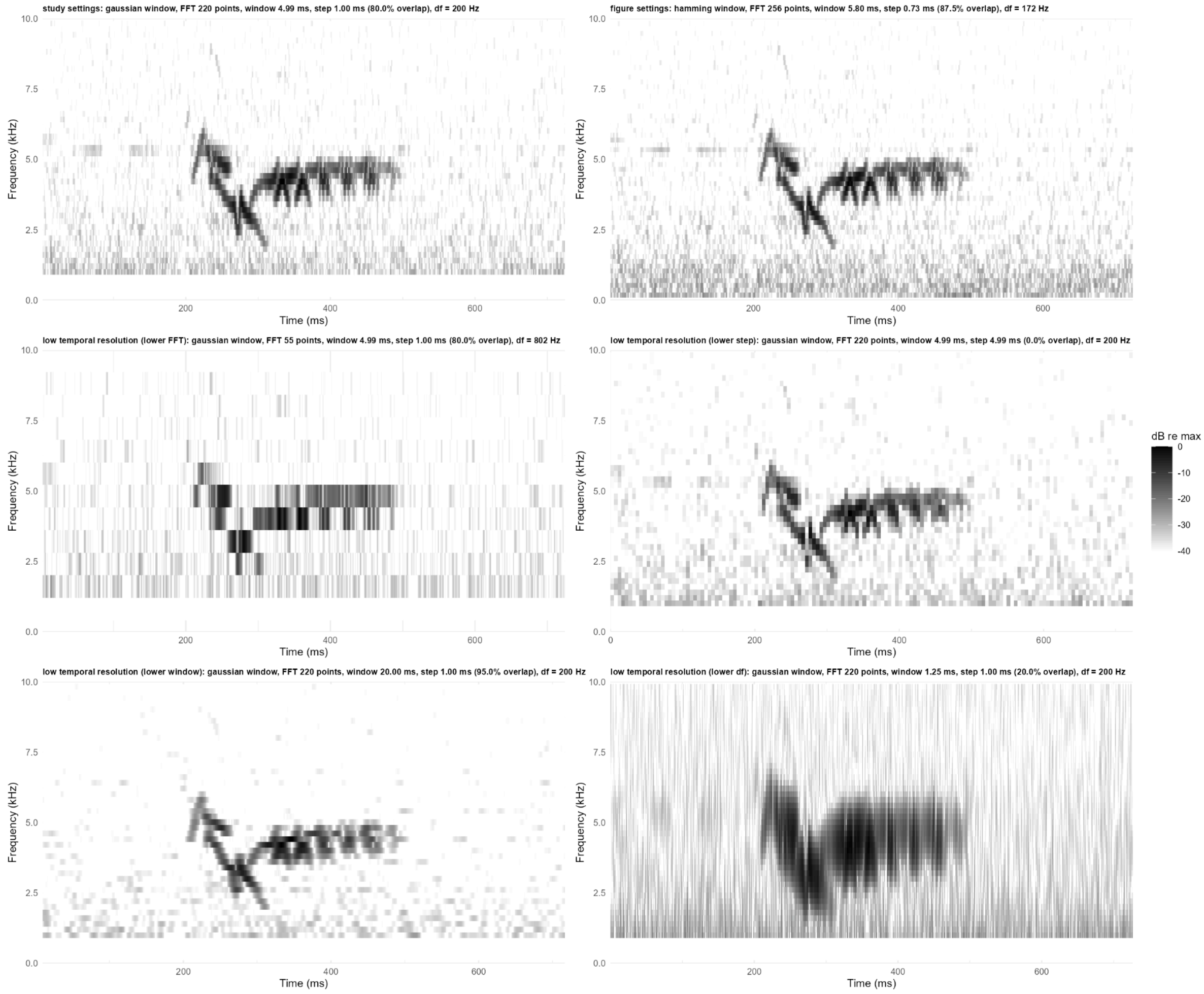

AM_03.23 ACAM/J899 20230519.WAV_3.wav

sampling rate 44100 Hz, 587 ms

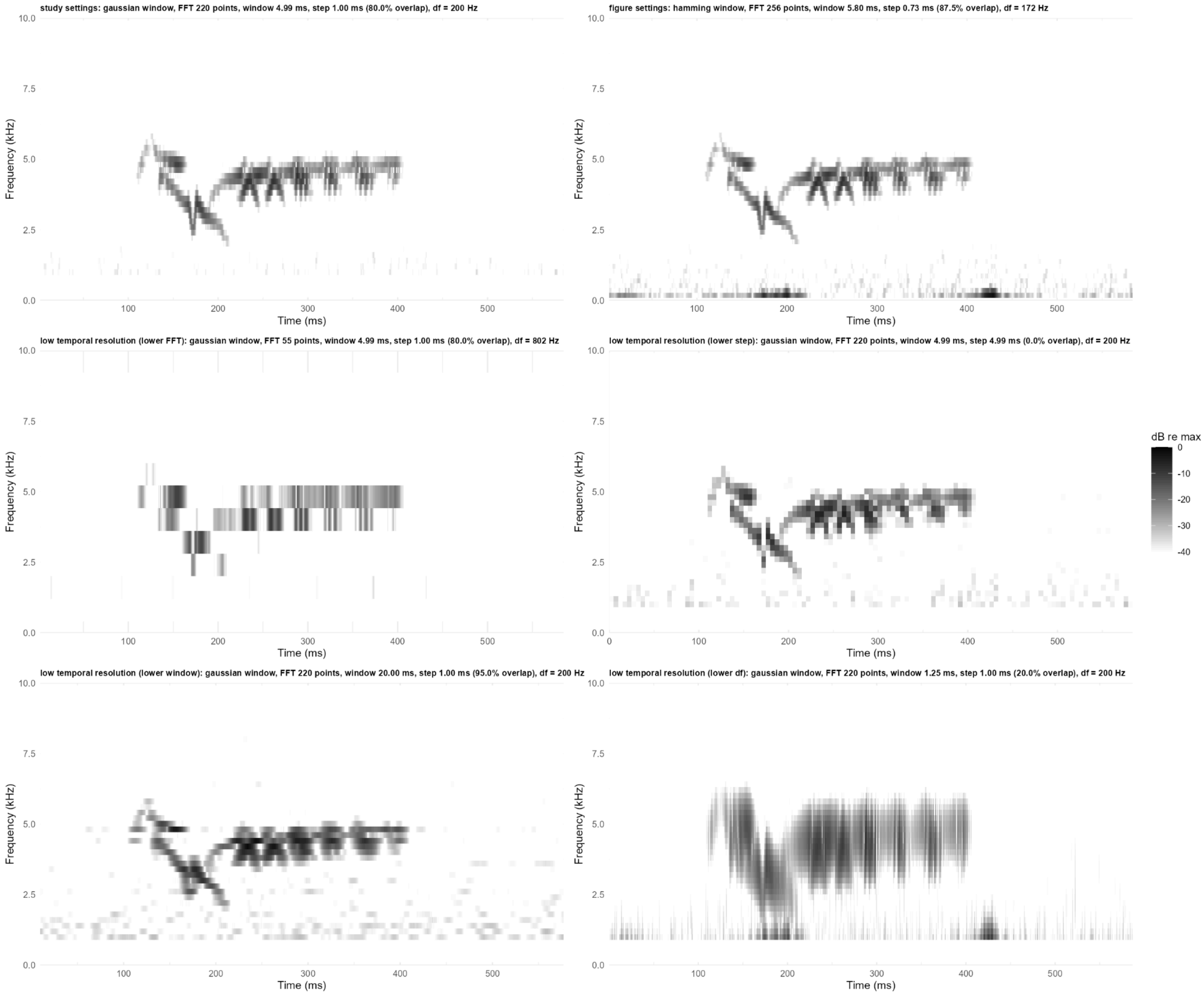

AM_04.23 ACAM/KUBI008 20230520.WAV_1.wav

sampling rate 44100 Hz, 590 ms

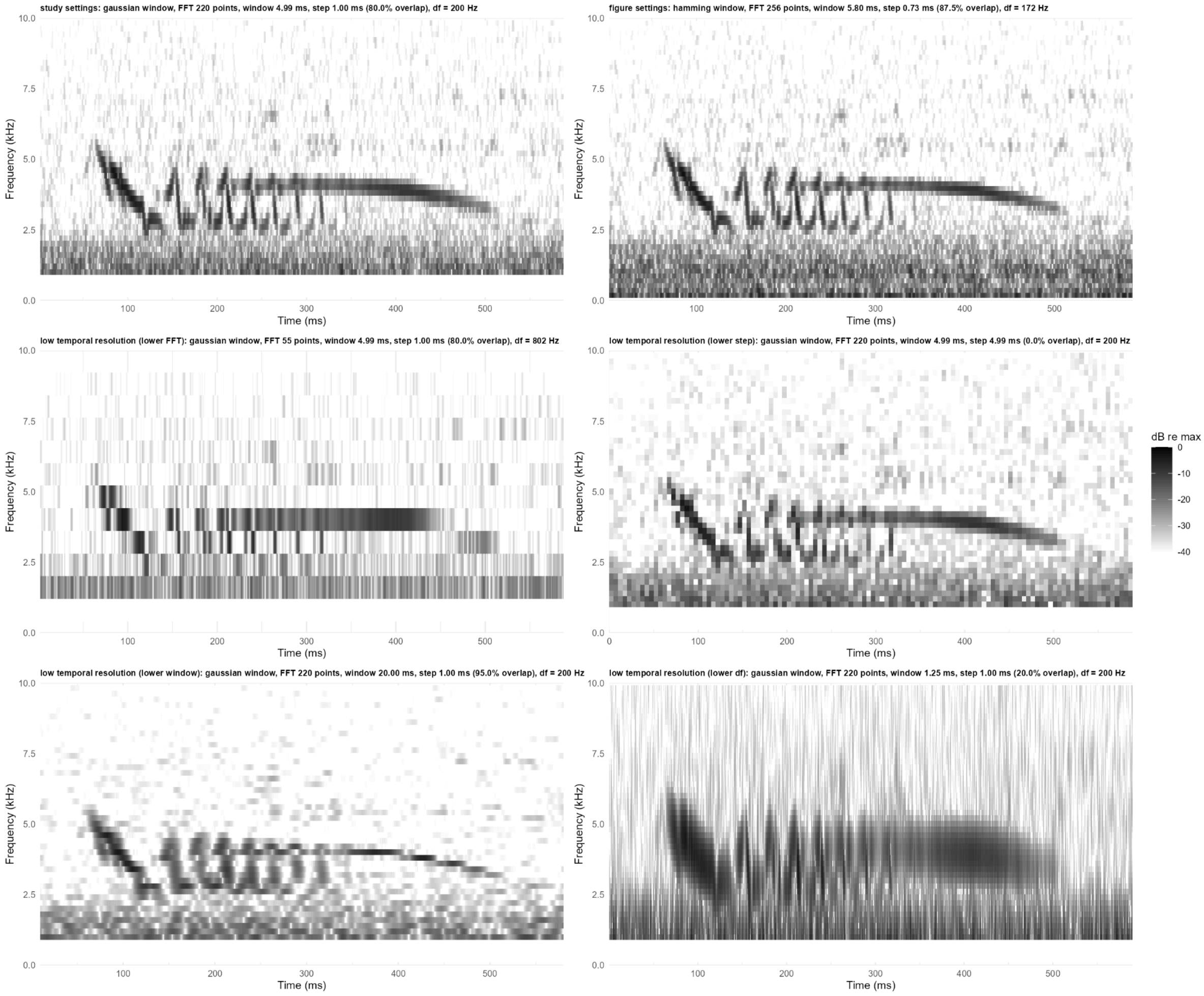

AM_04.23 ACAM/KUBI008 20230520.WAV_2.wav

sampling rate 44100 Hz, 532 ms

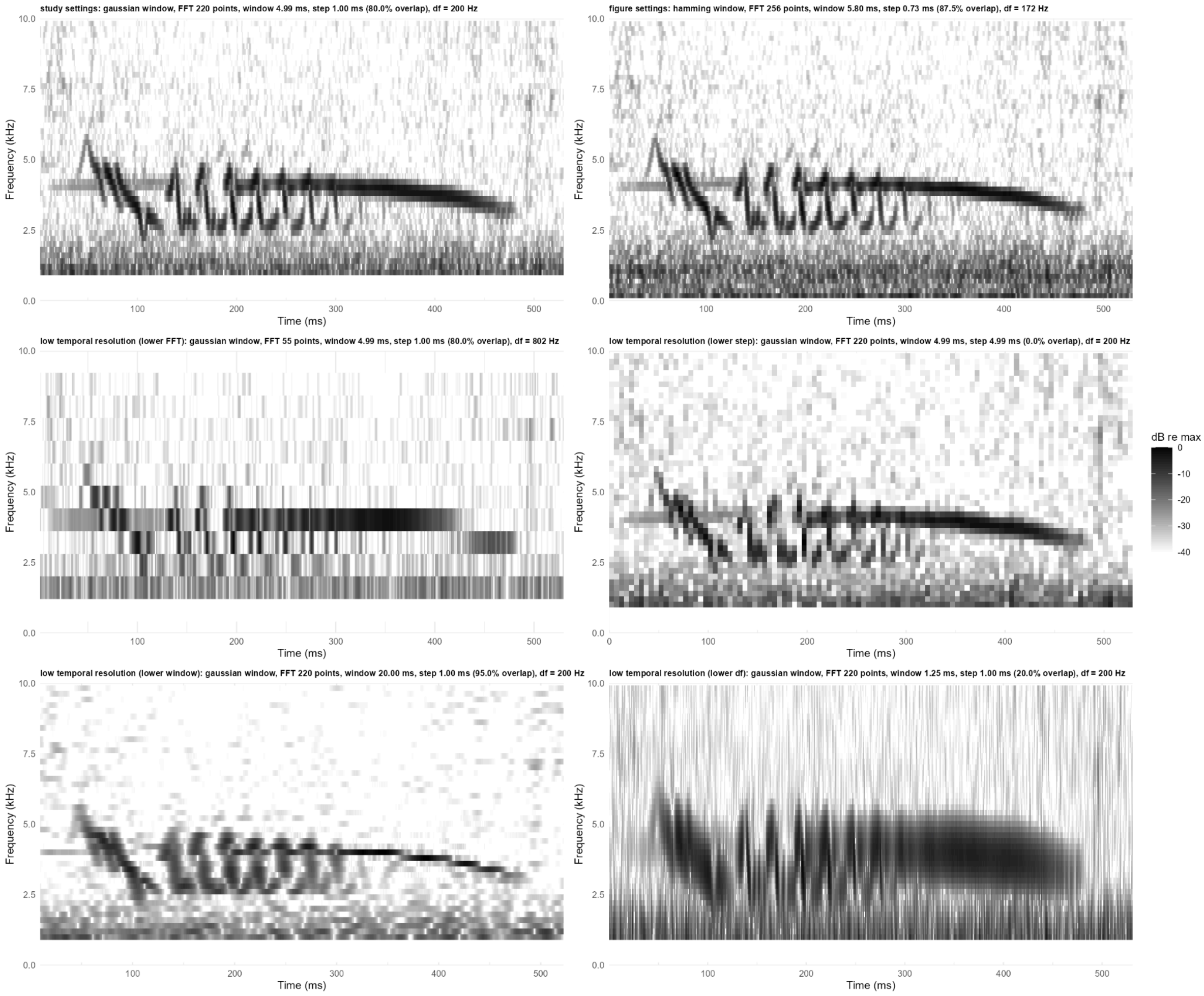

AM_04.23 ACAM/KUBI008 20230520.WAV_3.wav

sampling rate 44100 Hz, 542 ms

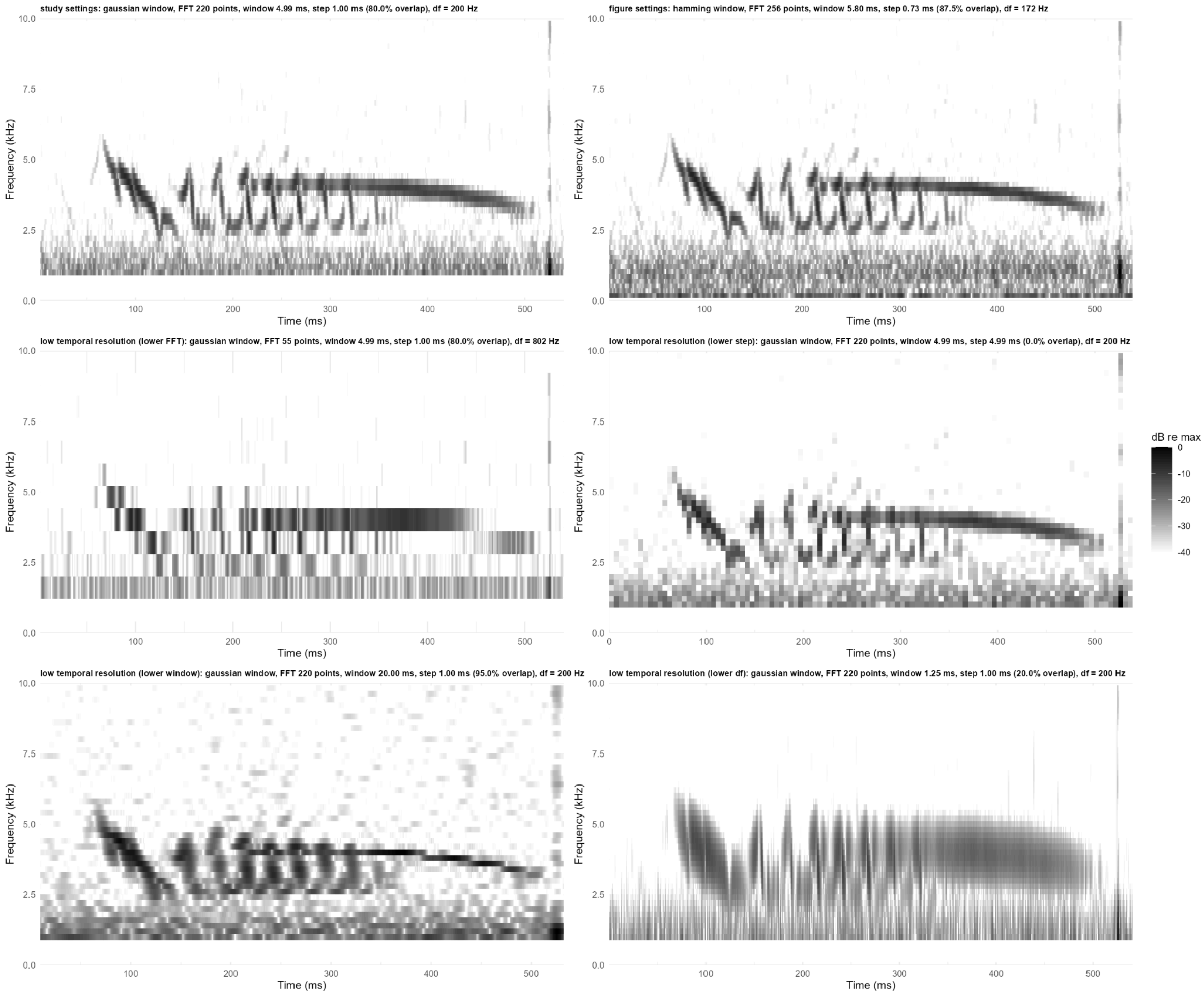

AM_17.23 ACAM/KUBI045 20230523.WAV_1.wav

sampling rate 44100 Hz, 563 ms

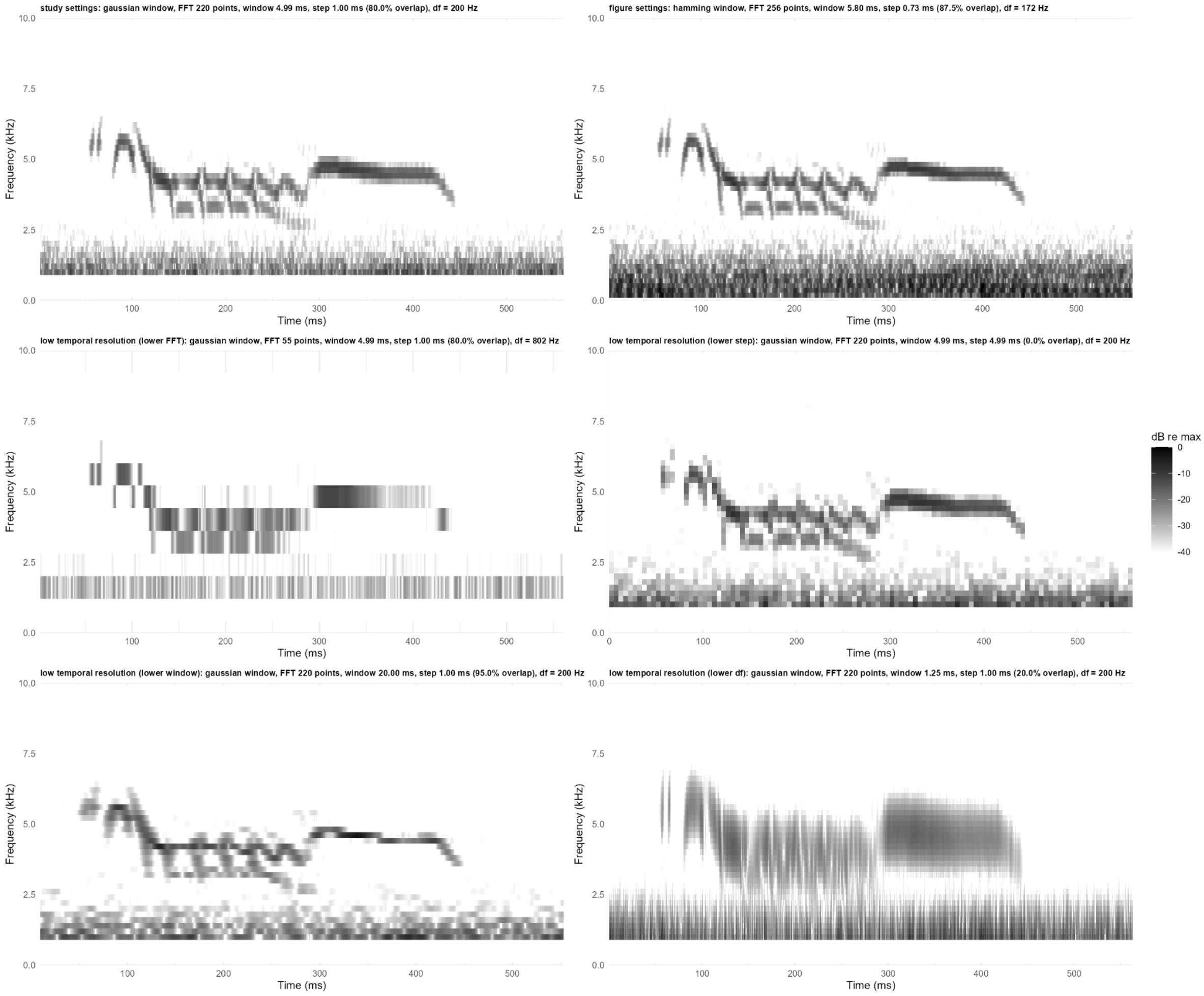

AM_17.23 ACAM/KUBI045 20230523.WAV_2.wav

sampling rate 44100 Hz, 595 ms

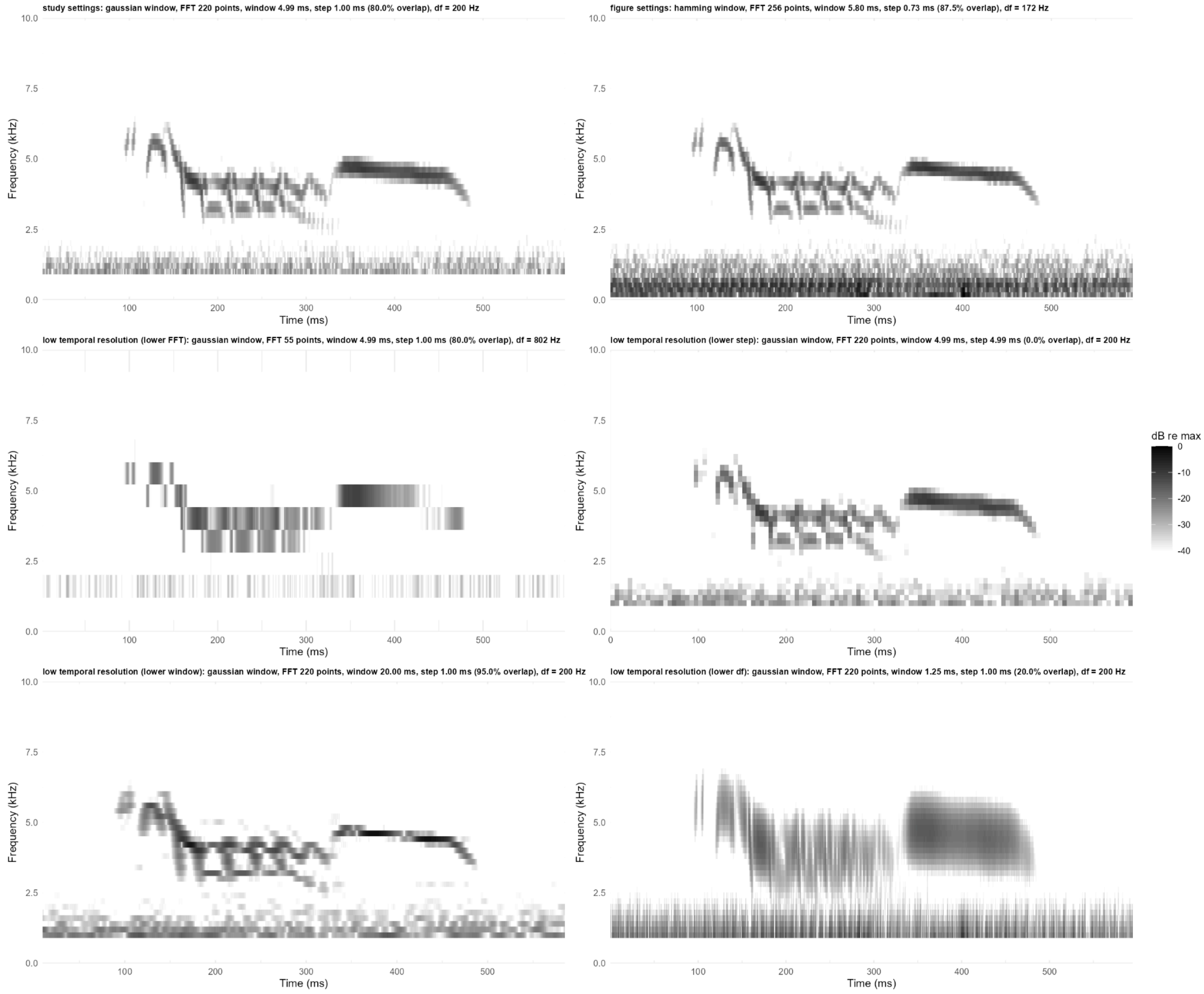

AM_17.23 ACAM/KUBI045 20230523.WAV_3.wav
sampling rate 44100 Hz, 557 ms

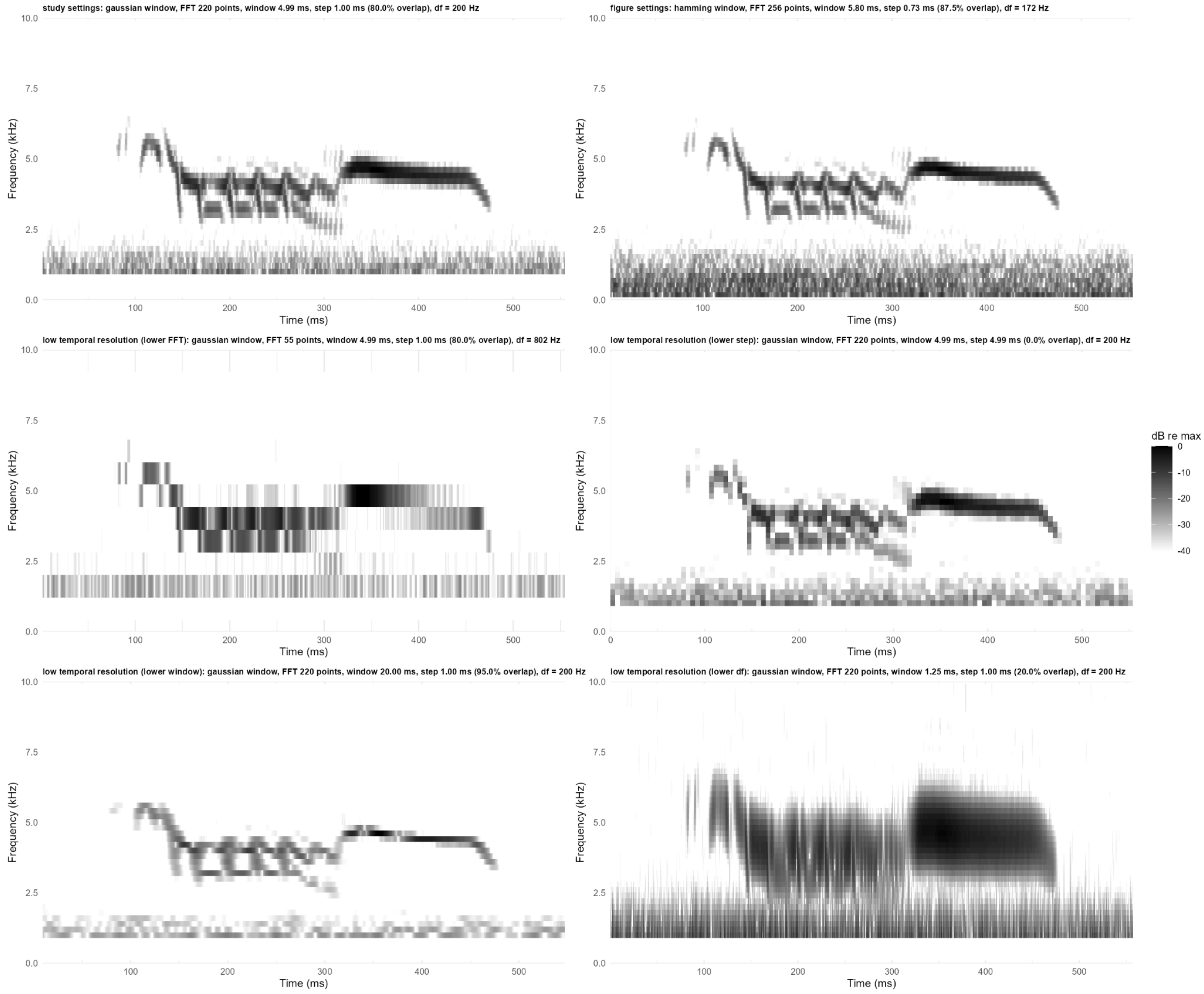

BRH_12.21 ACB/J277 20210511.WAV_1.wav

sampling rate 44100 Hz, 371 ms

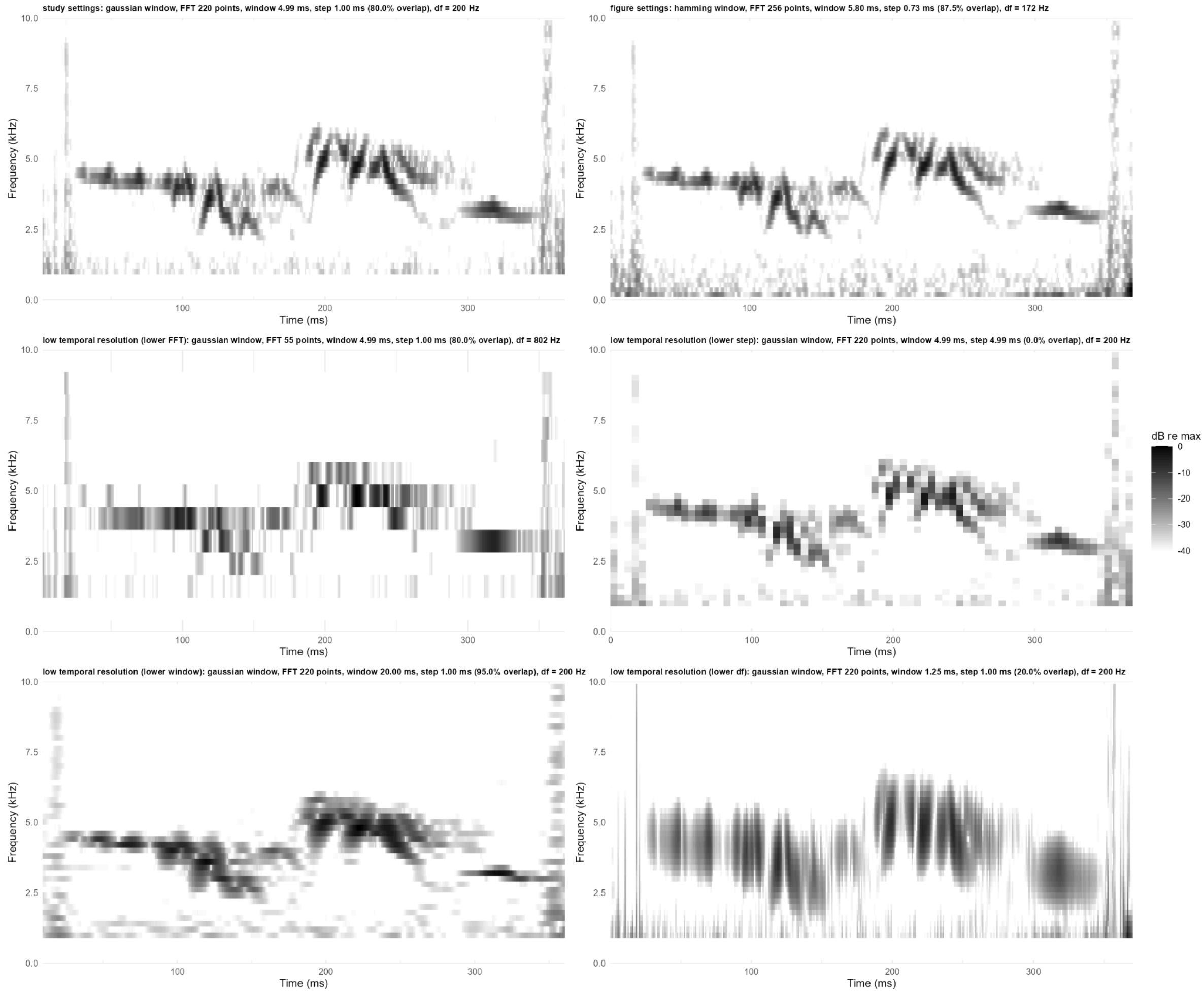

BRH_12.21 ACB/J277 20210511.WAV_2.wav

sampling rate 44100 Hz, 410 ms

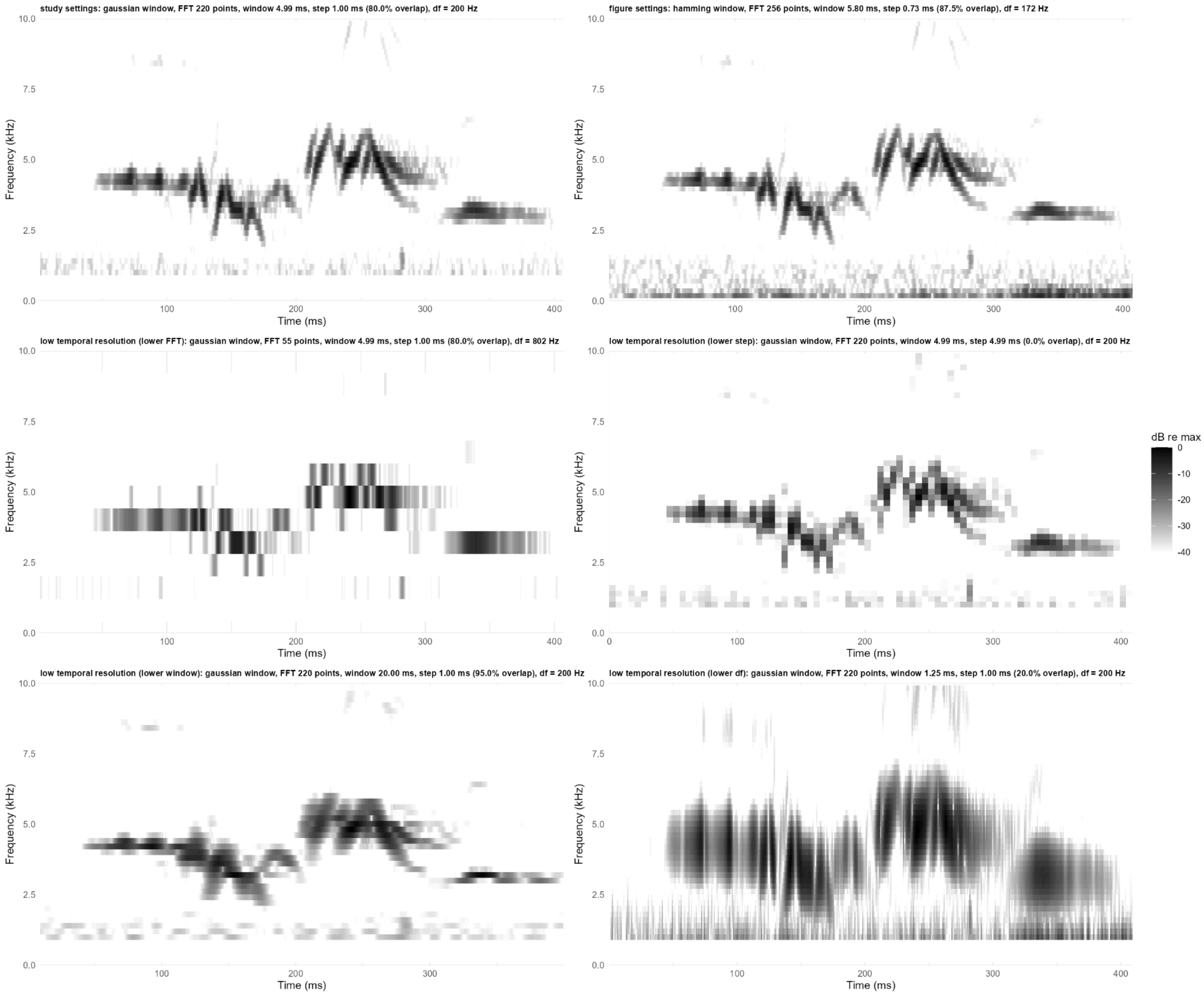

BRH_12.21 ACB/J277 20210511.WAV_3.wav

sampling rate 44100 Hz, 426 ms

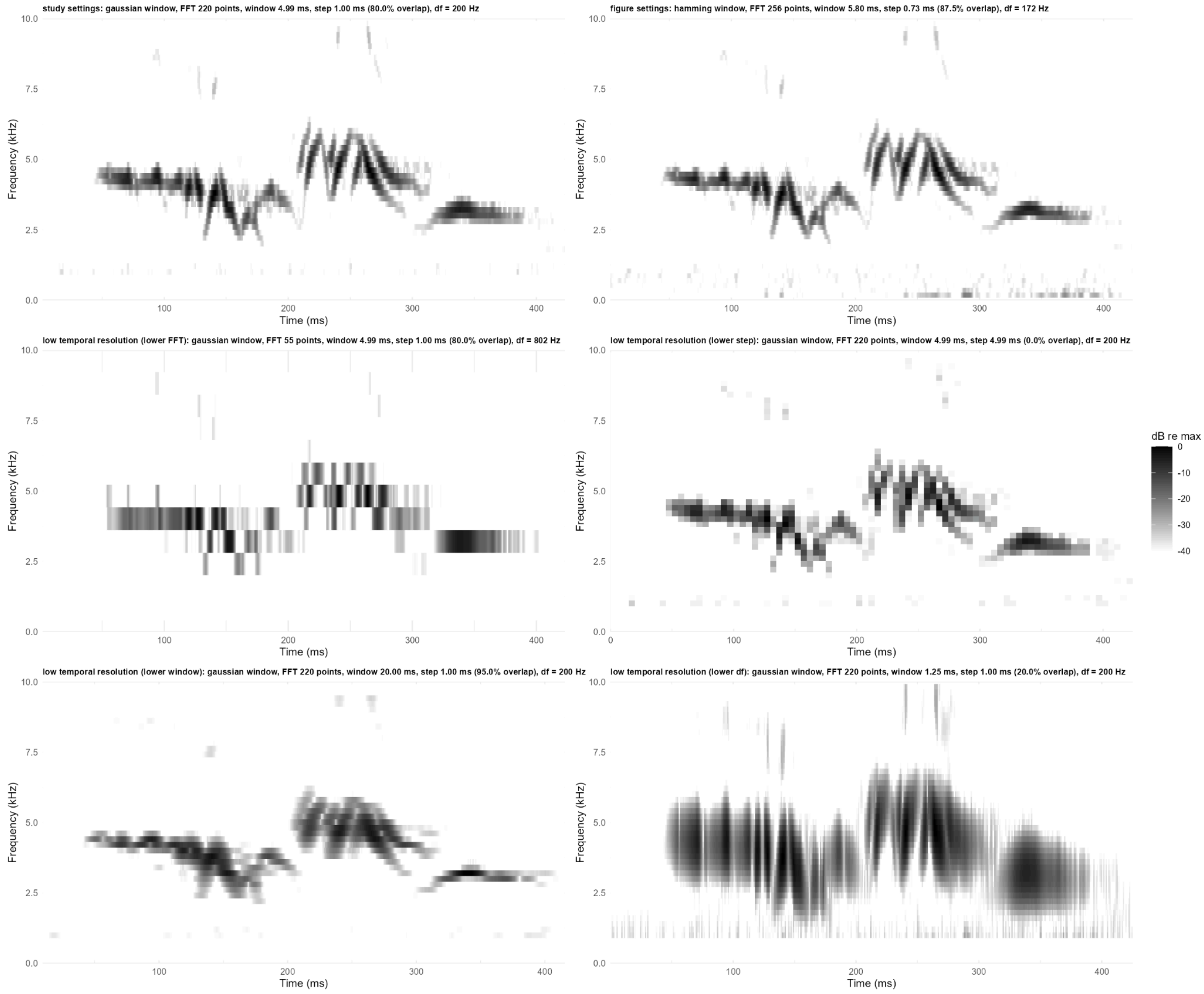

BRH_22.21 ACB_ringed/J362 20210513.WAV_1.wav

sampling rate 44100 Hz, 359 ms

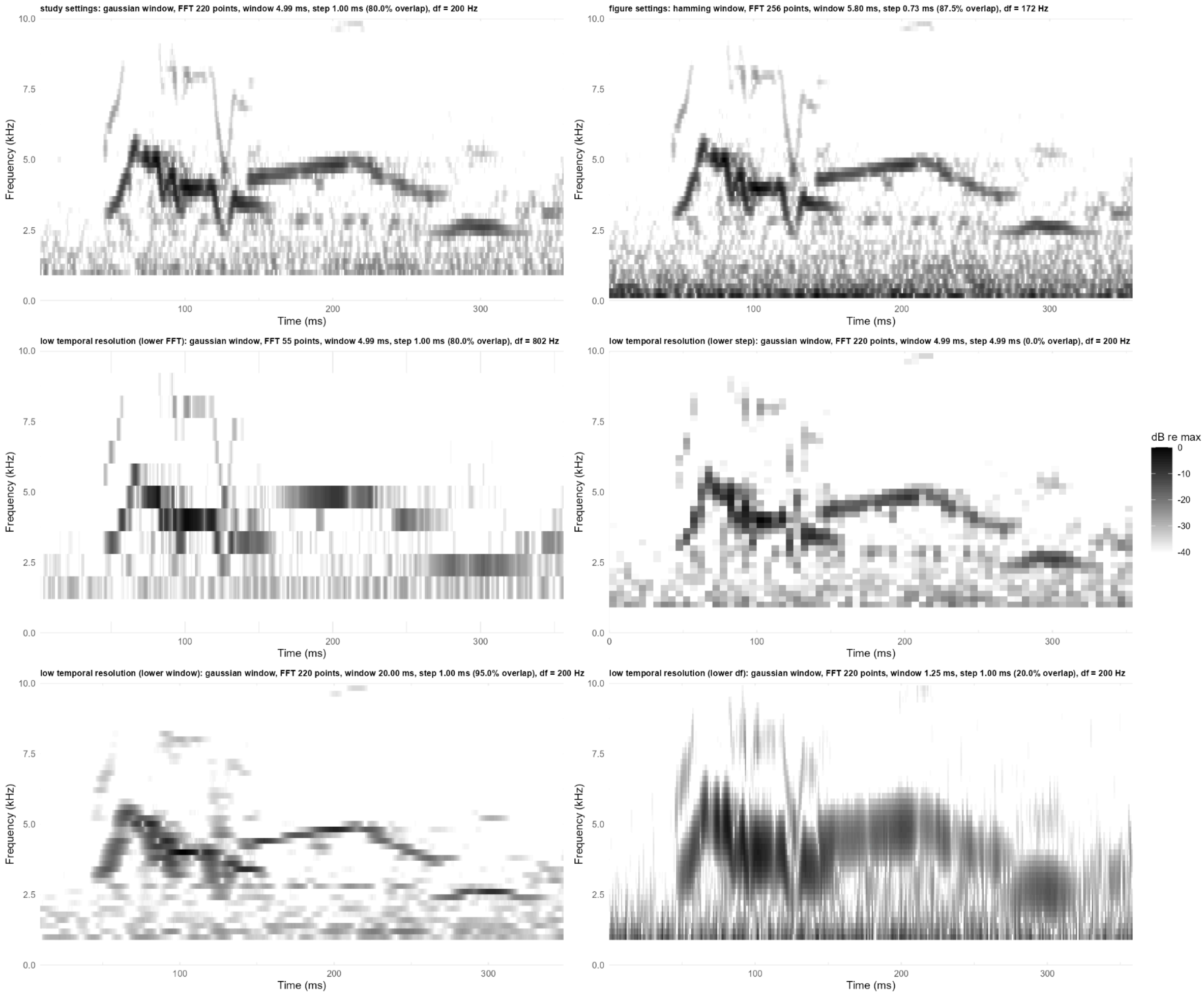

BRH_22.21 ACB_ringed/J362 20210513.WAV_2.wav

sampling rate 44100 Hz, 413 ms

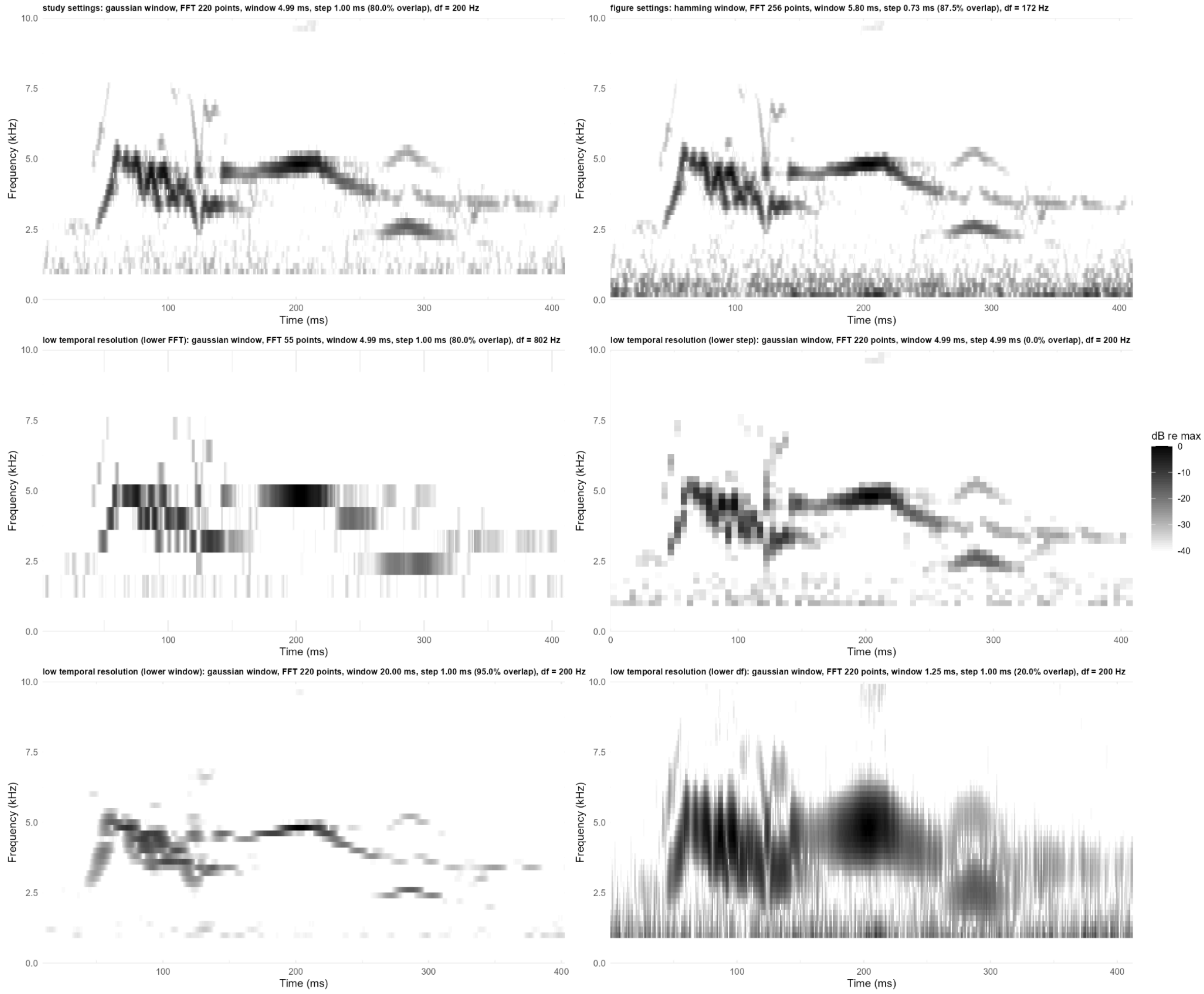

BRH_22.21 ACB_ringed/J362 20210513.WAV_3.wav

sampling rate 44100 Hz, 369 ms

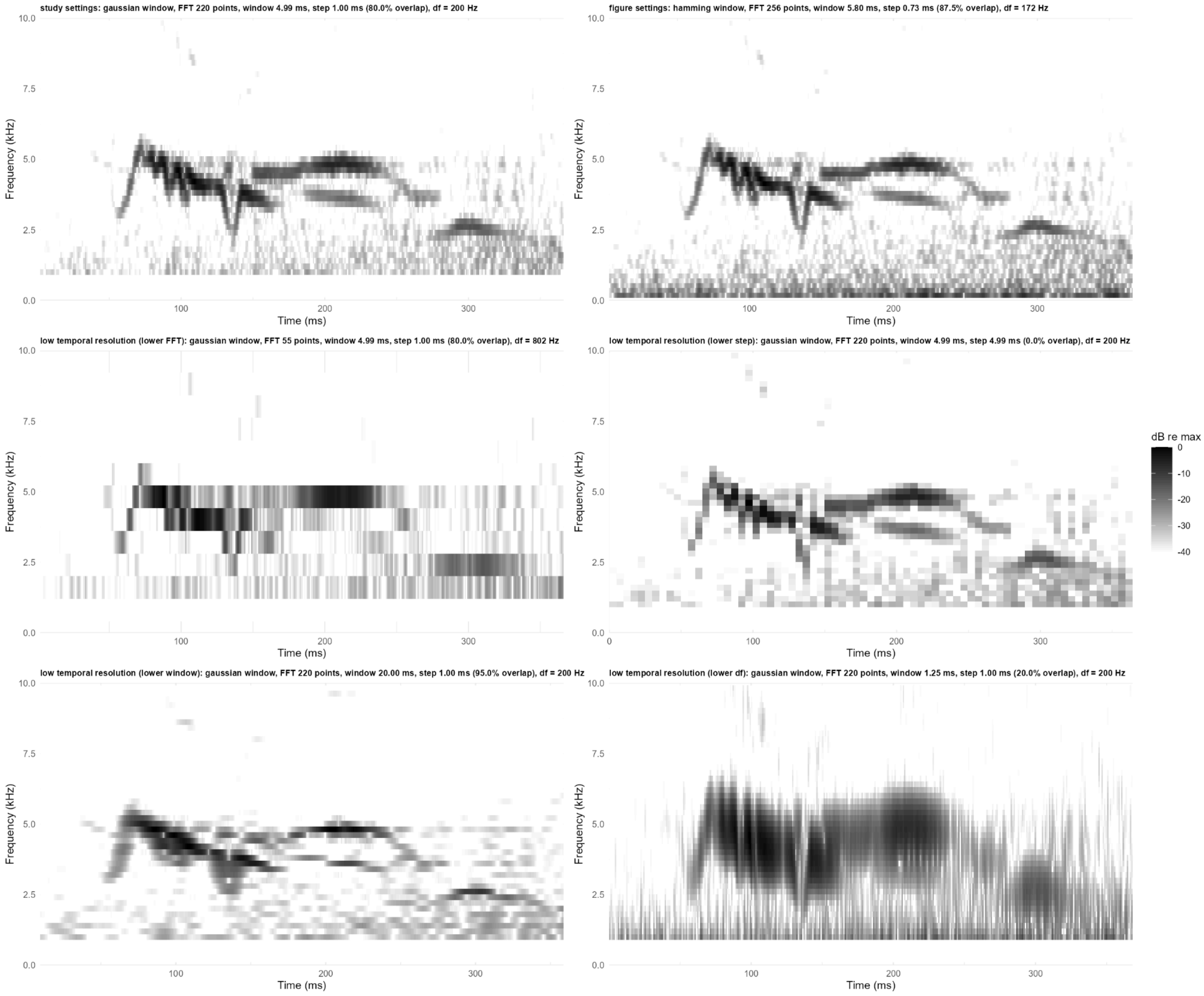

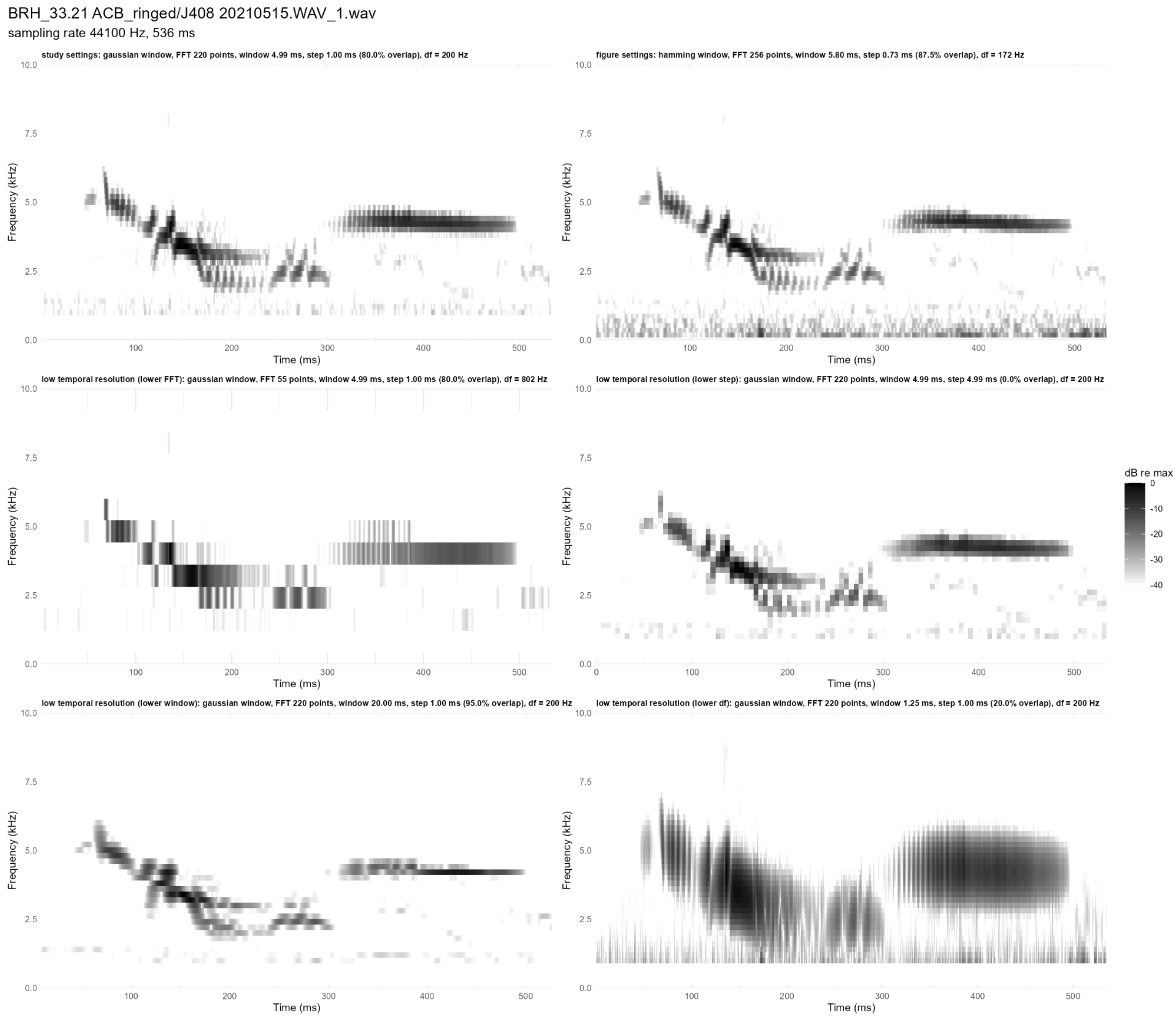
BRH_33.21 ACB_ringed/J408 20210515.WAV_1.wav
sampling rate 44100 Hz, 536 ms
study settings: gaussian window, FFT 220 points, window 4.99 ms, step 1.00 ms (80.0% overlap), df = 200 Hz
figure settings: hamming window, FFT 256 points, window 5.80 ms, step 0.73 ms (87.5% overlap), df = 172 Hz
low temporal resolution (lower FFT): gaussian window, FFT 55 points, window 4.99 ms, step 1.00 ms (80.0% overlap), df = 802 Hz
low temporal resolution (lower step): gaussian window, FFT 220 points, window 4.99 ms, step 4.99 ms (0.0% overlap), df = 200 Hz
low temporal resolution (lower window): gaussian window, FFT 220 points, window 20.00 ms, step 1.00 ms (95.0% overlap), df = 200 Hz
low temporal resolution (lower df): gaussian window, FFT 220 points, window 1.25 ms, step 1.00 ms (20.0% overlap), df = 200 Hz
Frequency (kHz)
Time (ms)
dB re max
0
-10
-20
-30
-40

BRH_33.21 ACB_ringed/J408 20210515.WAV_2.wav
sampling rate 44100 Hz, 500 ms

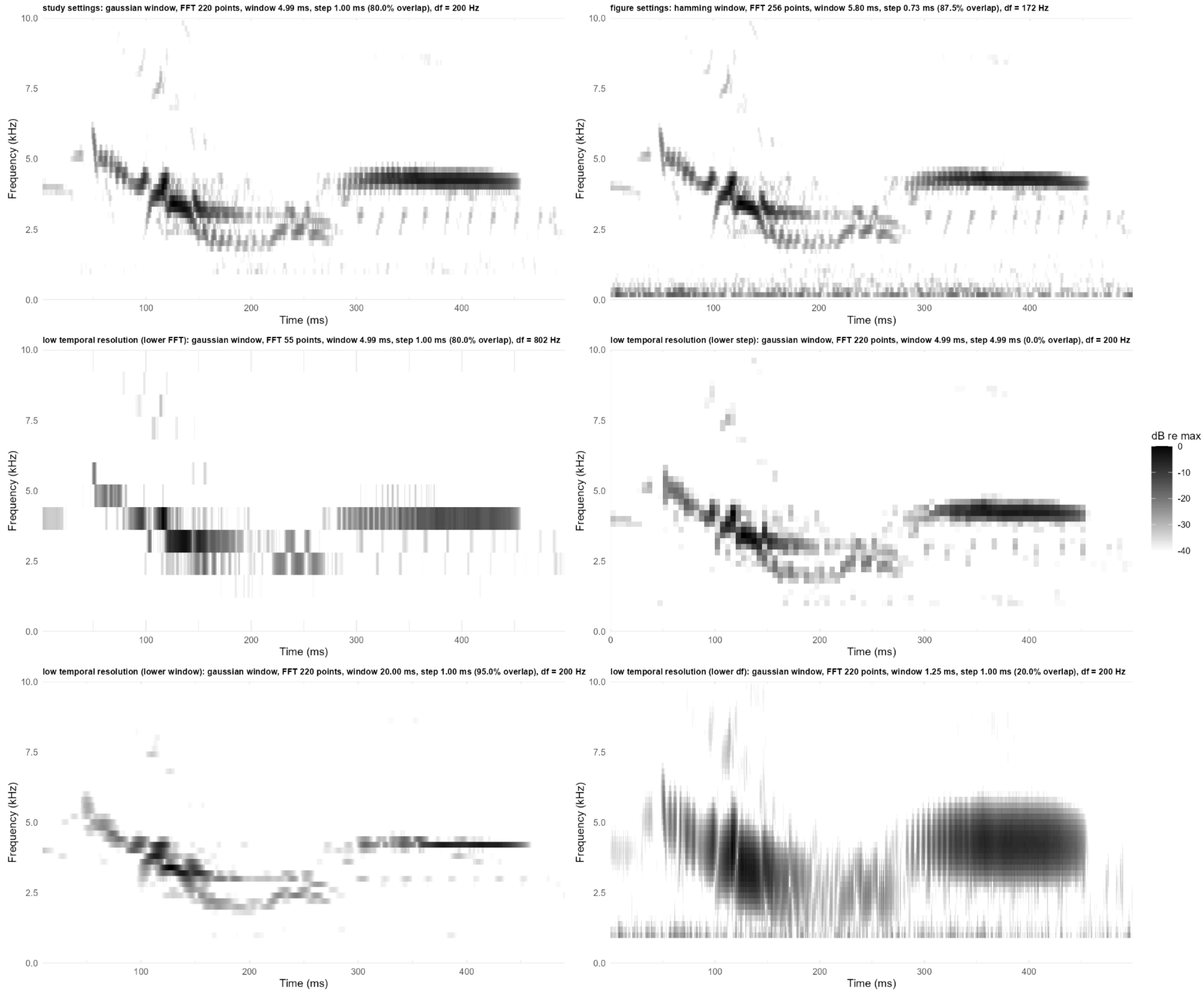

BRH_33.21 ACB_ringed/J408 20210515.WAV_3.wav

sampling rate 44100 Hz, 543 ms

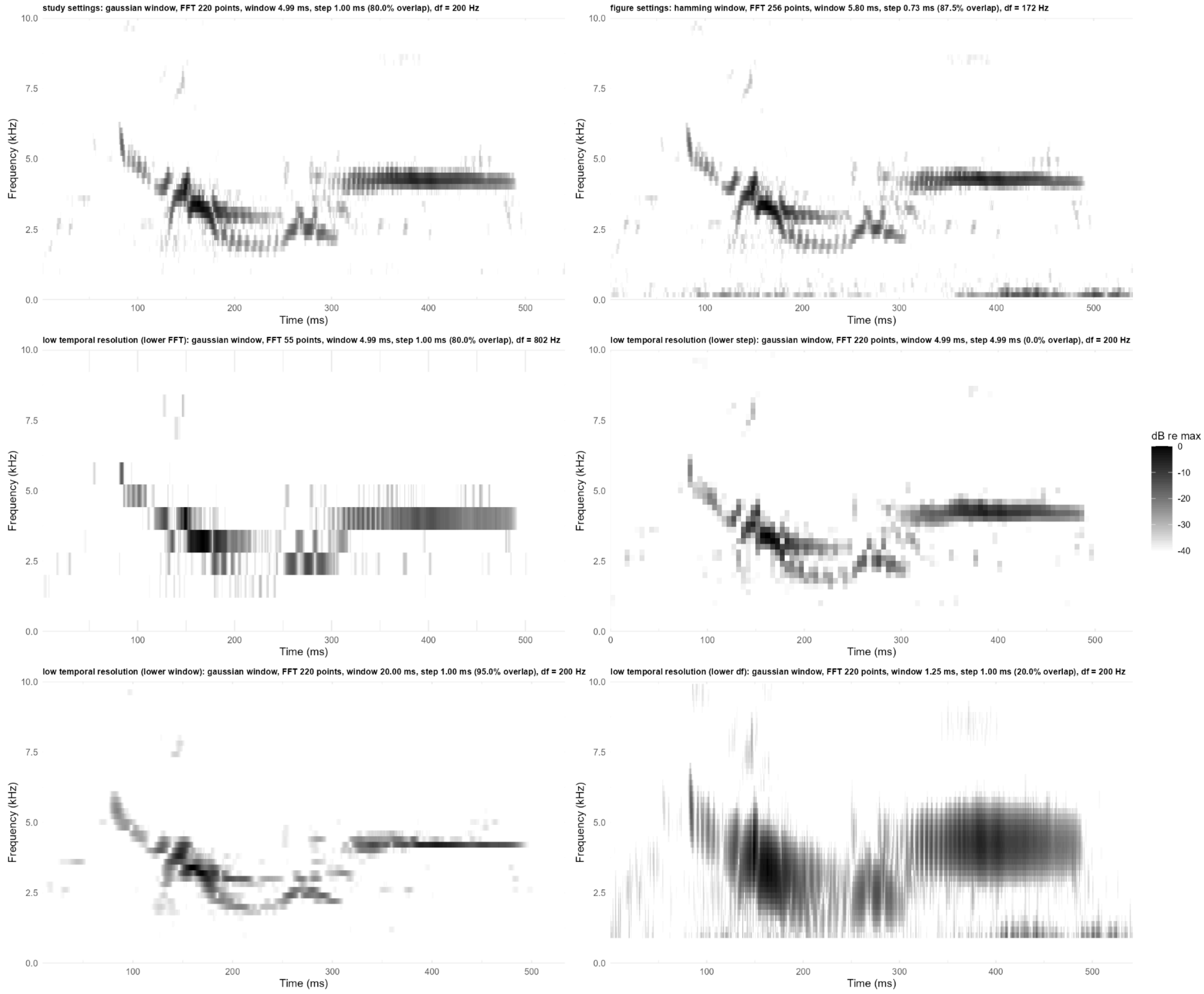

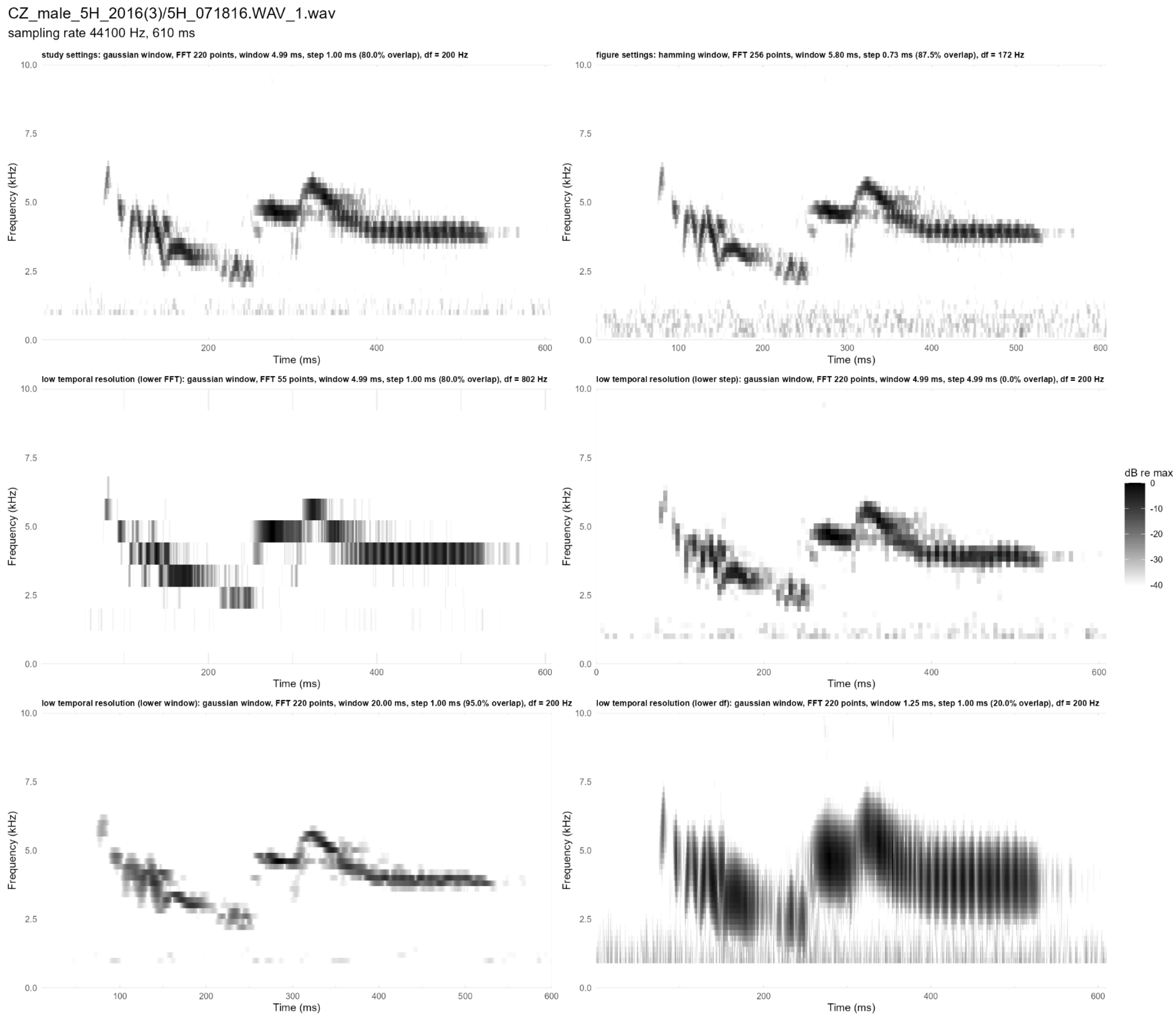

CZ_male_5H_2016(3)/5H_071816.WAV_1.wav
sampling rate 44100 Hz, 610 ms
study settings: gaussian window, FFT 220 points, window 4.99 ms, step 1.00 ms (80.0% overlap), df = 200 Hz
figure settings: hamming window, FFT 256 points, window 5.80 ms, step 0.73 ms (87.5% overlap), df = 172 Hz
low temporal resolution (lower FFT): gaussian window, FFT 55 points, window 4.99 ms, step 1.00 ms (80.0% overlap), df = 802 Hz
low temporal resolution (lower step): gaussian window, FFT 220 points, window 4.99 ms, step 4.99 ms (0.0% overlap), df = 200 Hz
low temporal resolution (lower window): gaussian window, FFT 220 points, window 20.00 ms, step 1.00 ms (95.0% overlap), df = 200 Hz
low temporal resolution (lower df): gaussian window, FFT 220 points, window 1.25 ms, step 1.00 ms (20.0% overlap), df = 200 Hz
Frequency (kHz)
Time (ms)
dB re max

CZ_male_5H_2016(3)/5H_071816.WAV_2.wav

sampling rate 44100 Hz, 587 ms

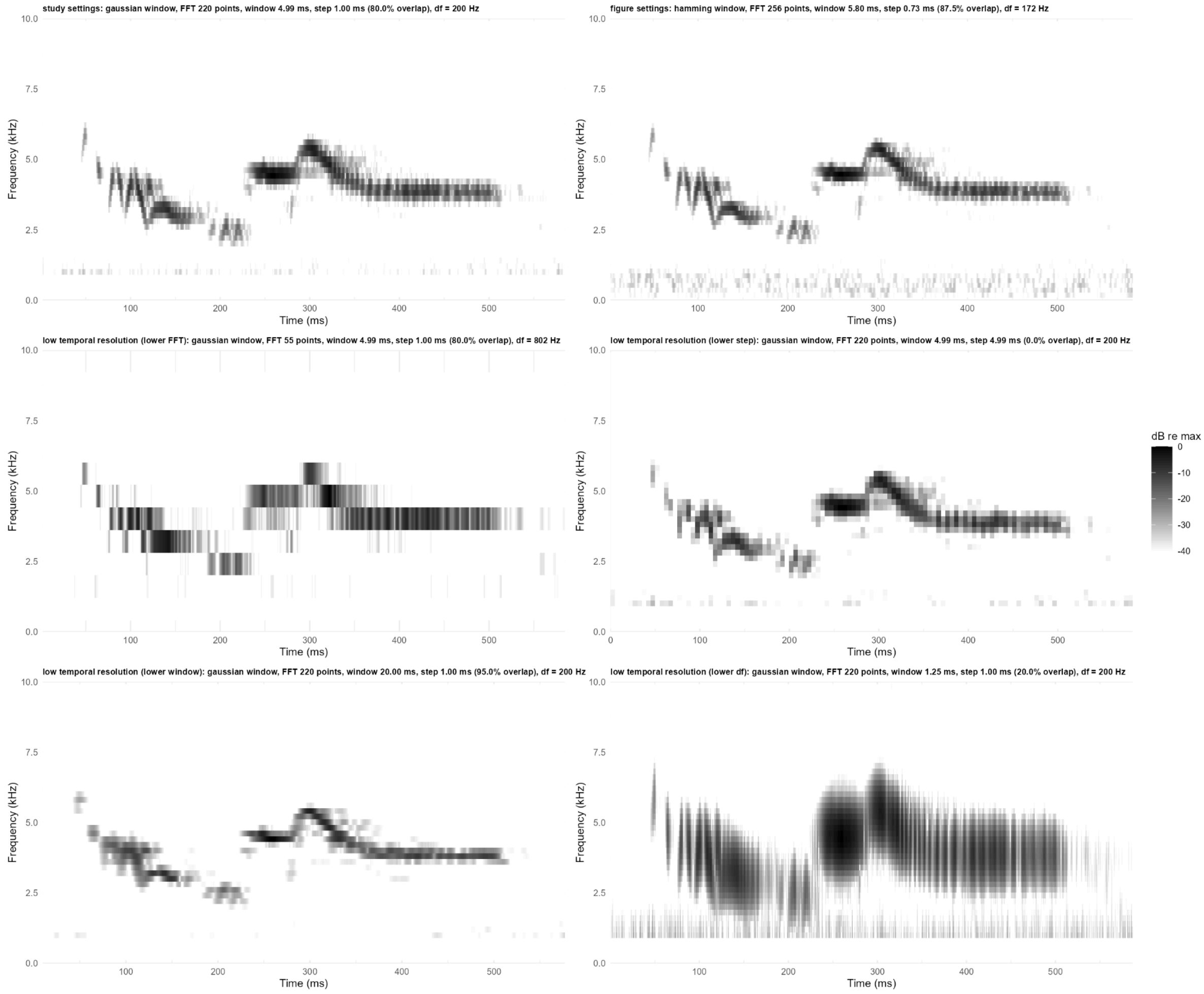

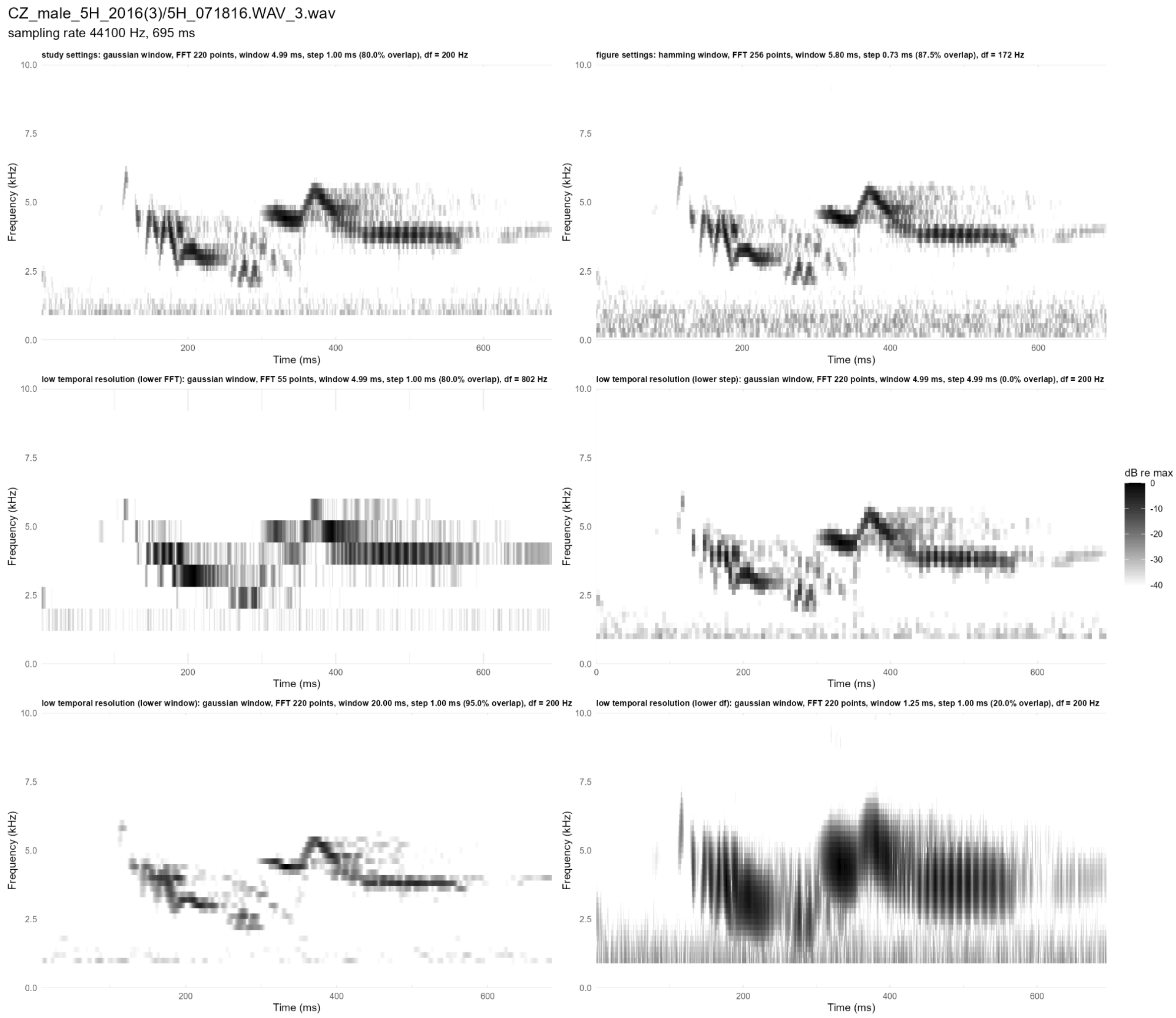

CZ_male_5H_2016(3)/5H_071816.WAV_3.wav
sampling rate 44100 Hz, 695 ms
study settings: gaussian window, FFT 220 points, window 4.99 ms, step 1.00 ms (80.0% overlap), df = 200 Hz
figure settings: hamming window, FFT 256 points, window 5.80 ms, step 0.73 ms (87.5% overlap), df = 172 Hz
low temporal resolution (lower FFT): gaussian window, FFT 55 points, window 4.99 ms, step 1.00 ms (80.0% overlap), df = 802 Hz
low temporal resolution (lower step): gaussian window, FFT 220 points, window 4.99 ms, step 4.99 ms (0.0% overlap), df = 200 Hz
low temporal resolution (lower window): gaussian window, FFT 220 points, window 20.00 ms, step 1.00 ms (95.0% overlap), df = 200 Hz
low temporal resolution (lower df): gaussian window, FFT 220 points, window 1.25 ms, step 1.00 ms (20.0% overlap), df = 200 Hz
Frequency (kHz)
Time (ms)
dB re max

CZ_male_5T.16 ACCZ/5T_061815_1.WAV_1.wav

sampling rate 44100 Hz, 535 ms

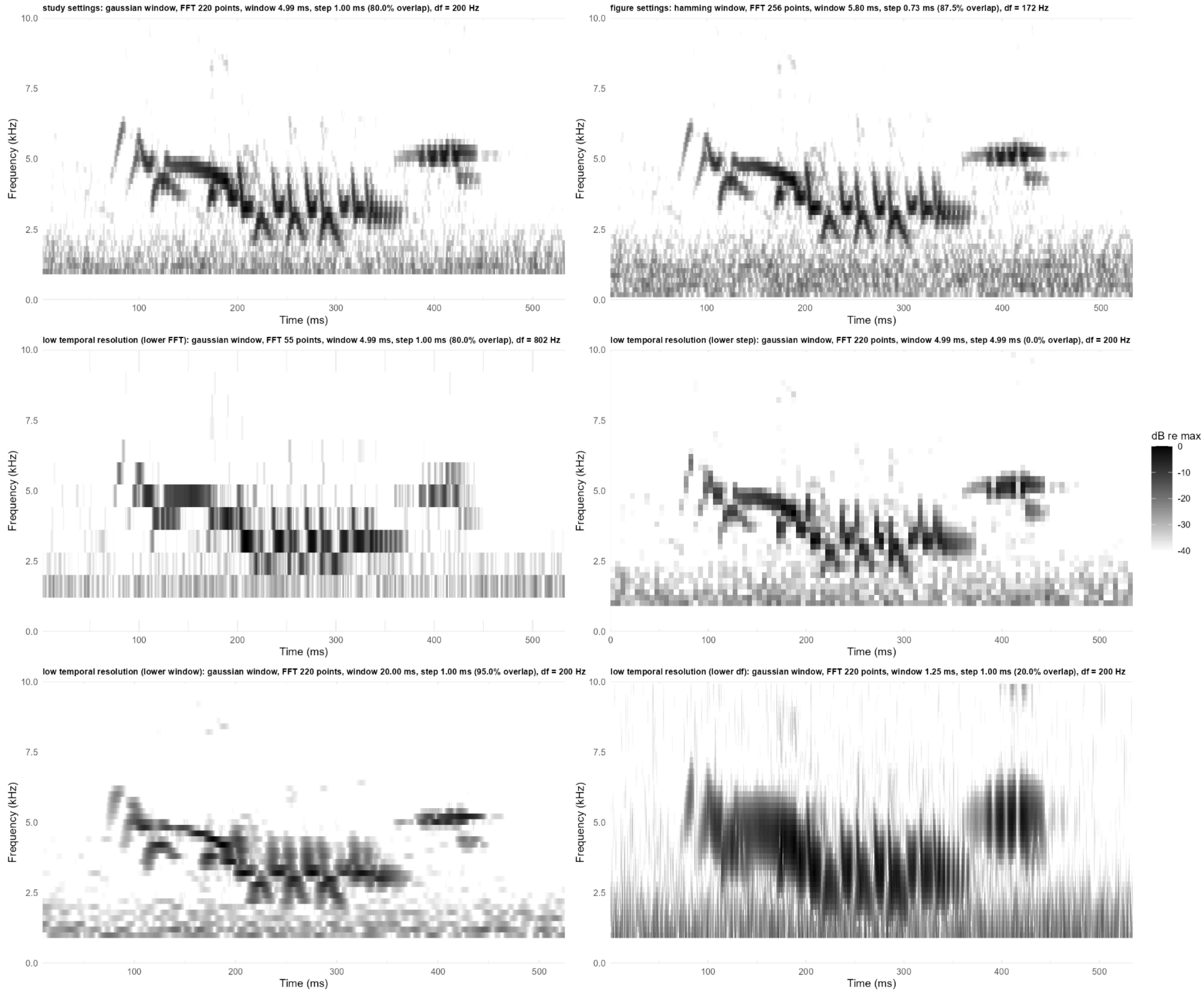

CZ_male_5T.16 ACCZ/5T_061815_1.WAV_2.wav

sampling rate 44100 Hz, 534 ms

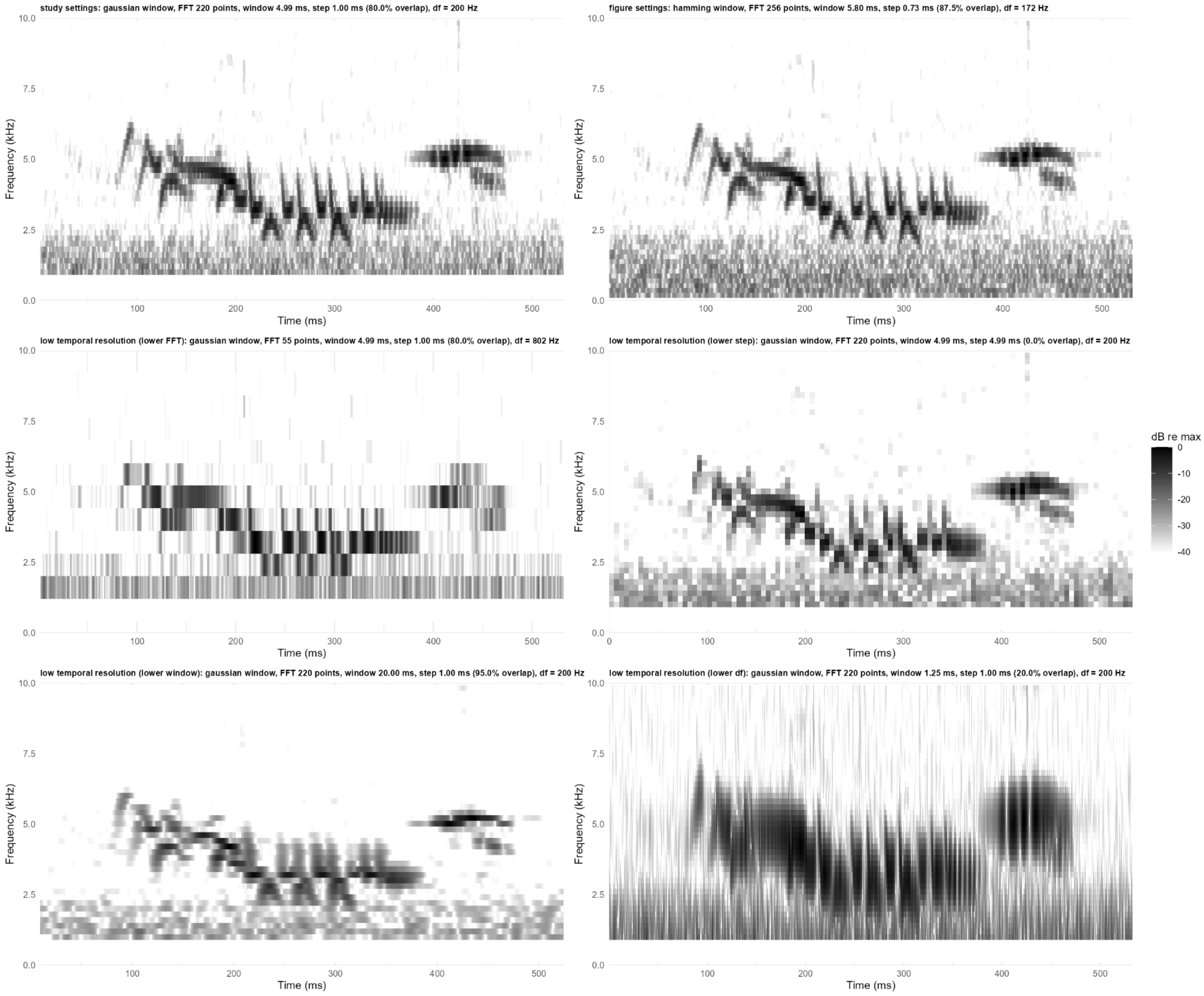

CZ_male_5T.16 ACCZ/5T_061815_1.WAV_3.wav

sampling rate 44100 Hz, 506 ms

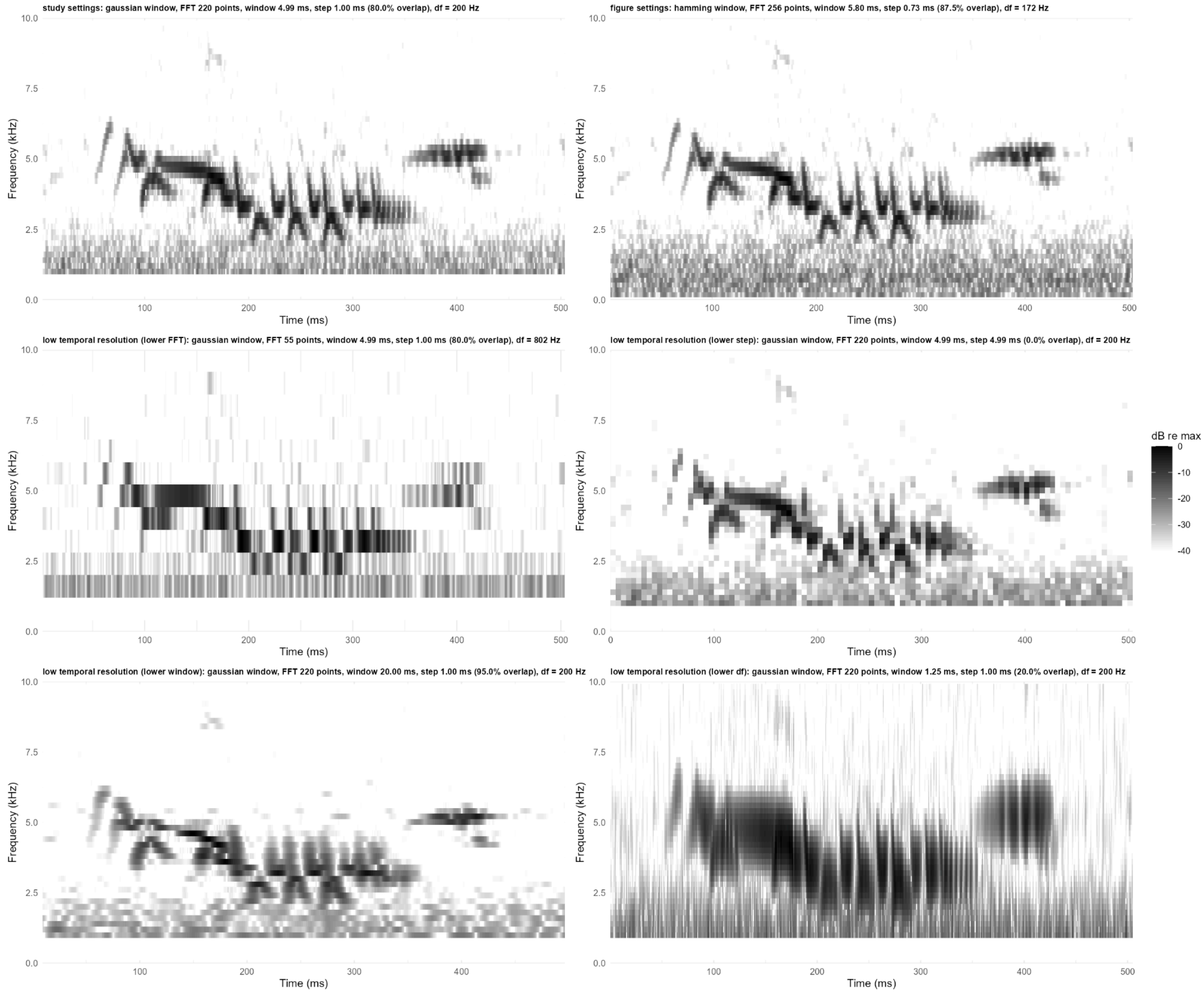

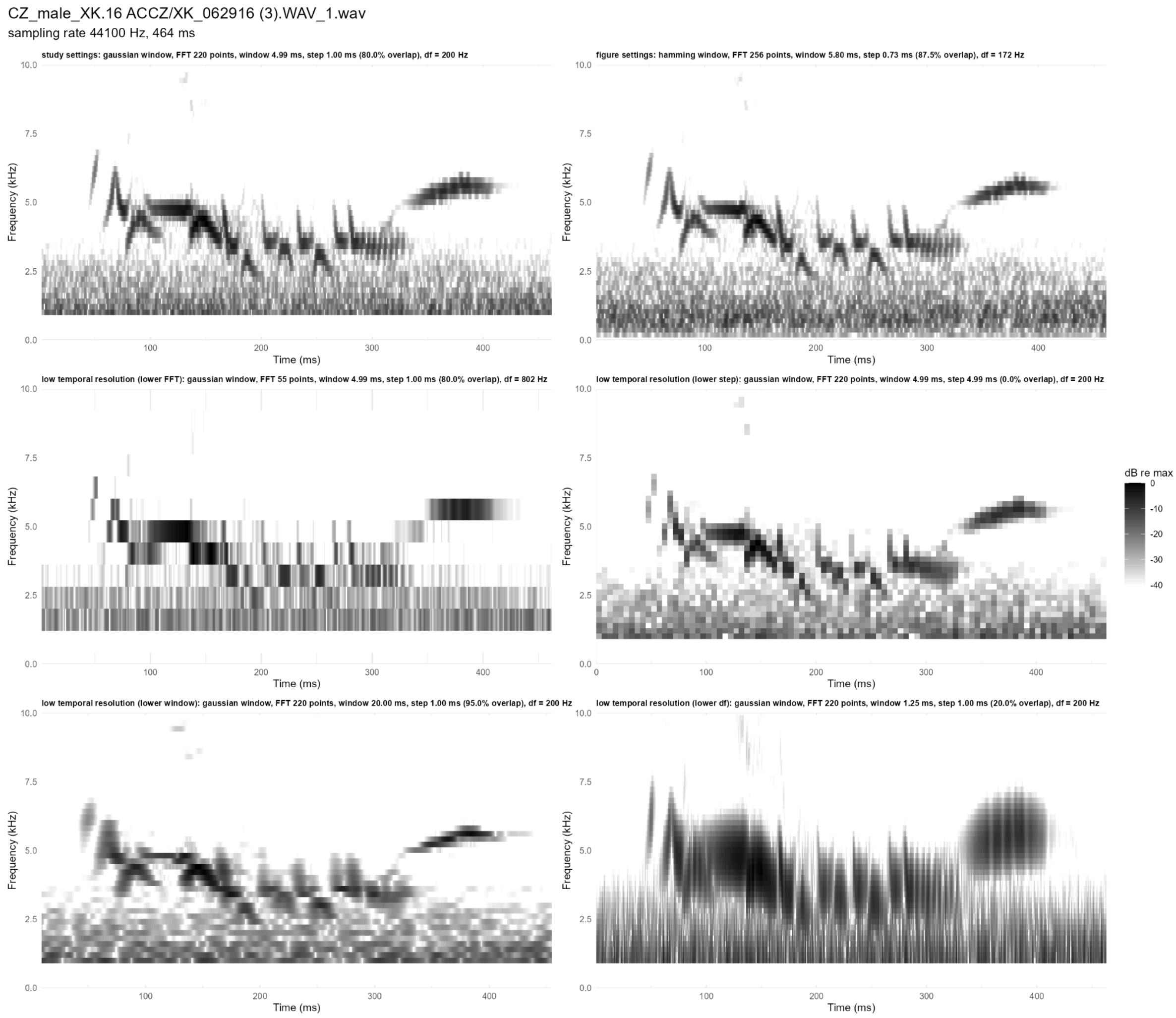
CZ_male_XK.16 ACCZ/XK_062916 (3).WAV_1.wav
sampling rate 44100 Hz, 464 ms
study settings: gaussian window, FFT 220 points, window 4.99 ms, step 1.00 ms (80.0% overlap), df = 200 Hz
figure settings: hamming window, FFT 256 points, window 5.80 ms, step 0.73 ms (87.5% overlap), df = 172 Hz
low temporal resolution (lower FFT): gaussian window, FFT 55 points, window 4.99 ms, step 1.00 ms (80.0% overlap), df = 802 Hz
low temporal resolution (lower step): gaussian window, FFT 220 points, window 4.99 ms, step 4.99 ms (0.0% overlap), df = 200 Hz
low temporal resolution (lower window): gaussian window, FFT 220 points, window 20.00 ms, step 1.00 ms (95.0% overlap), df = 200 Hz
low temporal resolution (lower df): gaussian window, FFT 220 points, window 1.25 ms, step 1.00 ms (20.0% overlap), df = 200 Hz
Frequency (kHz)
Time (ms)
dB re max
0
-10
-20
-30
-40

CZ_male_XK.16 ACCZ/XK_062916 (3).WAV_2.wav
sampling rate 44100 Hz, 439 ms

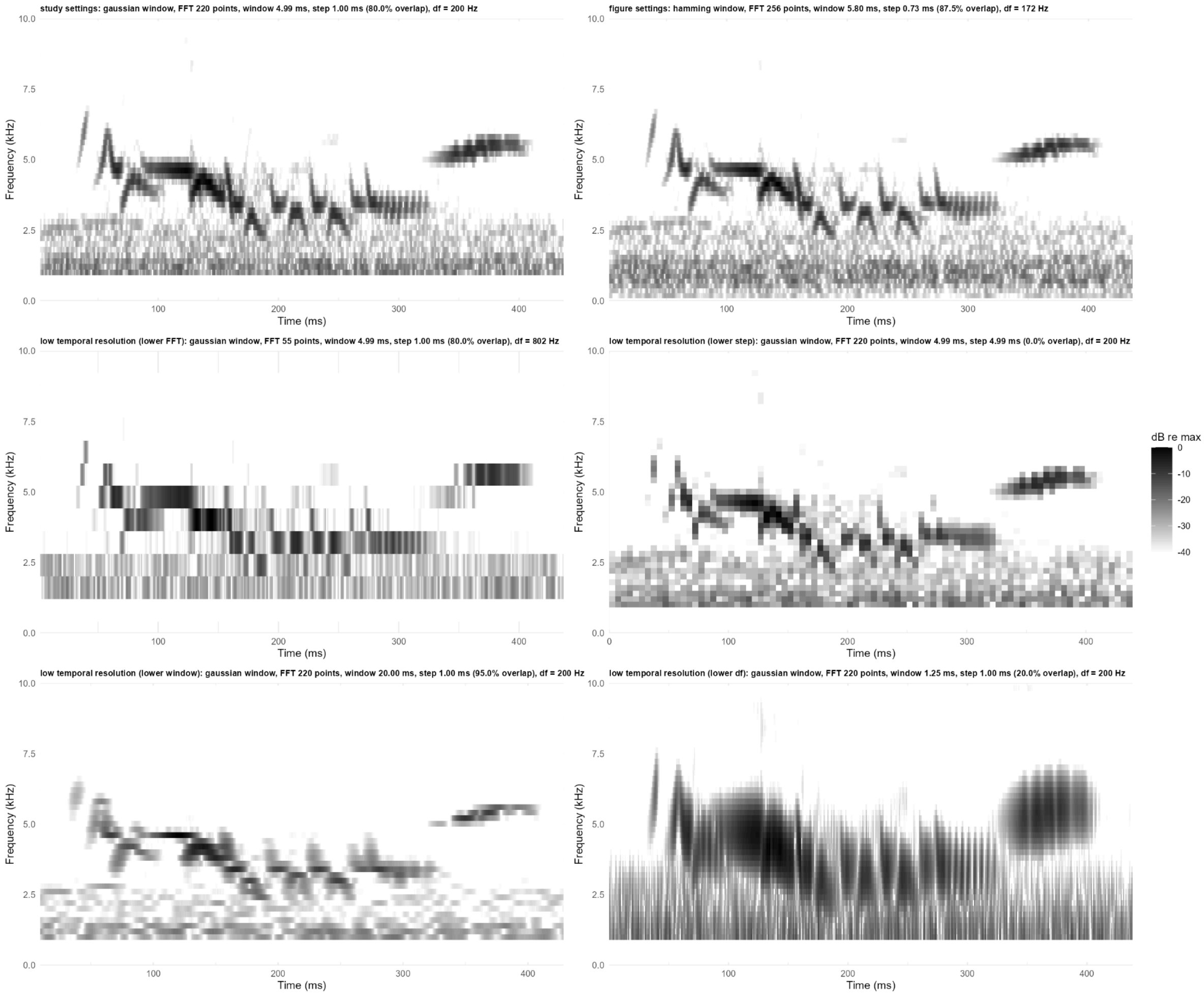

CZ_male_XK.16 ACCZ/XK_062916 (3).WAV_3.wav

sampling rate 44100 Hz, 443 ms

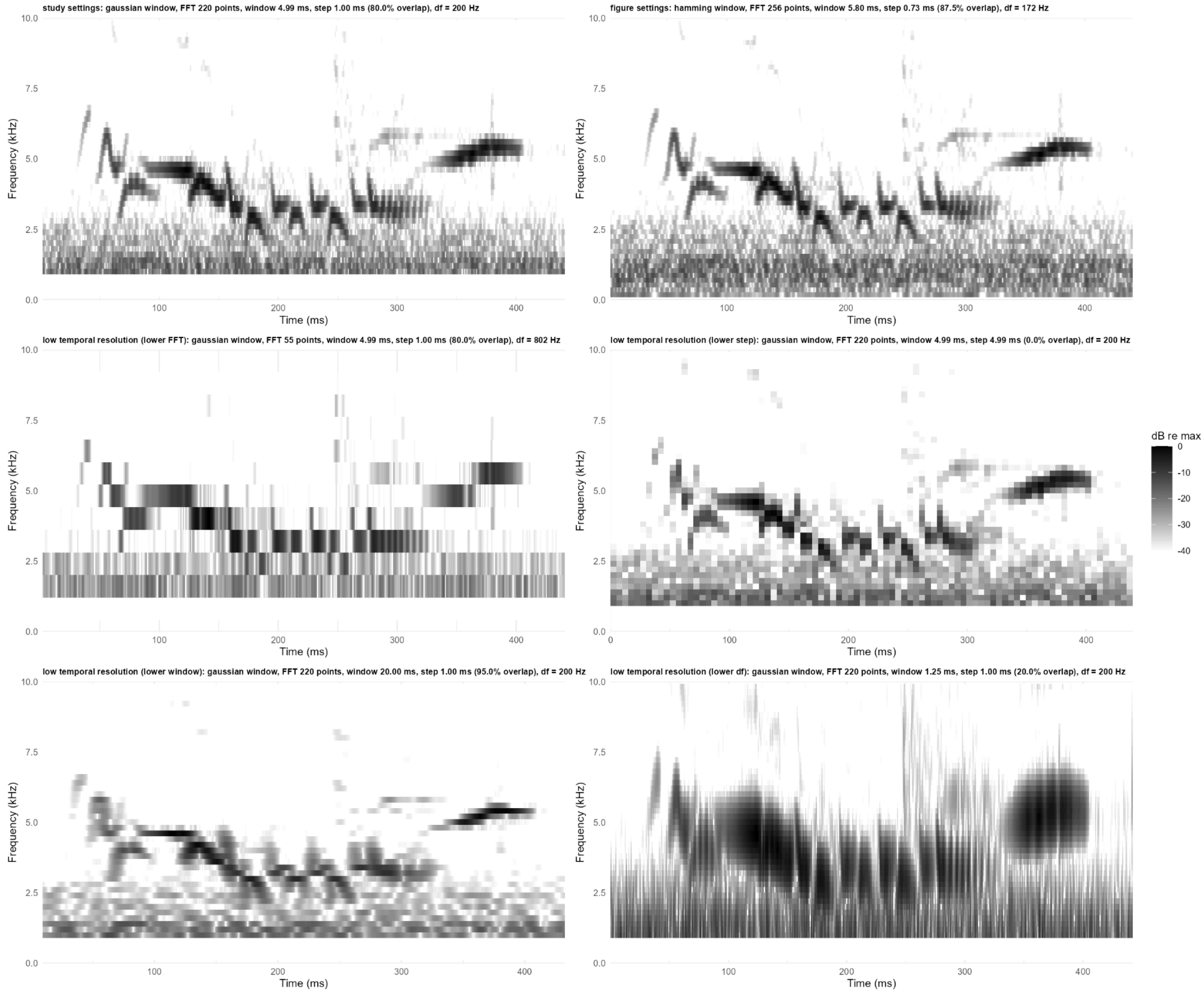

GS_05.20/05.20 ACGS_ 1463_ed_1.wav

sampling rate 44100 Hz, 528 ms

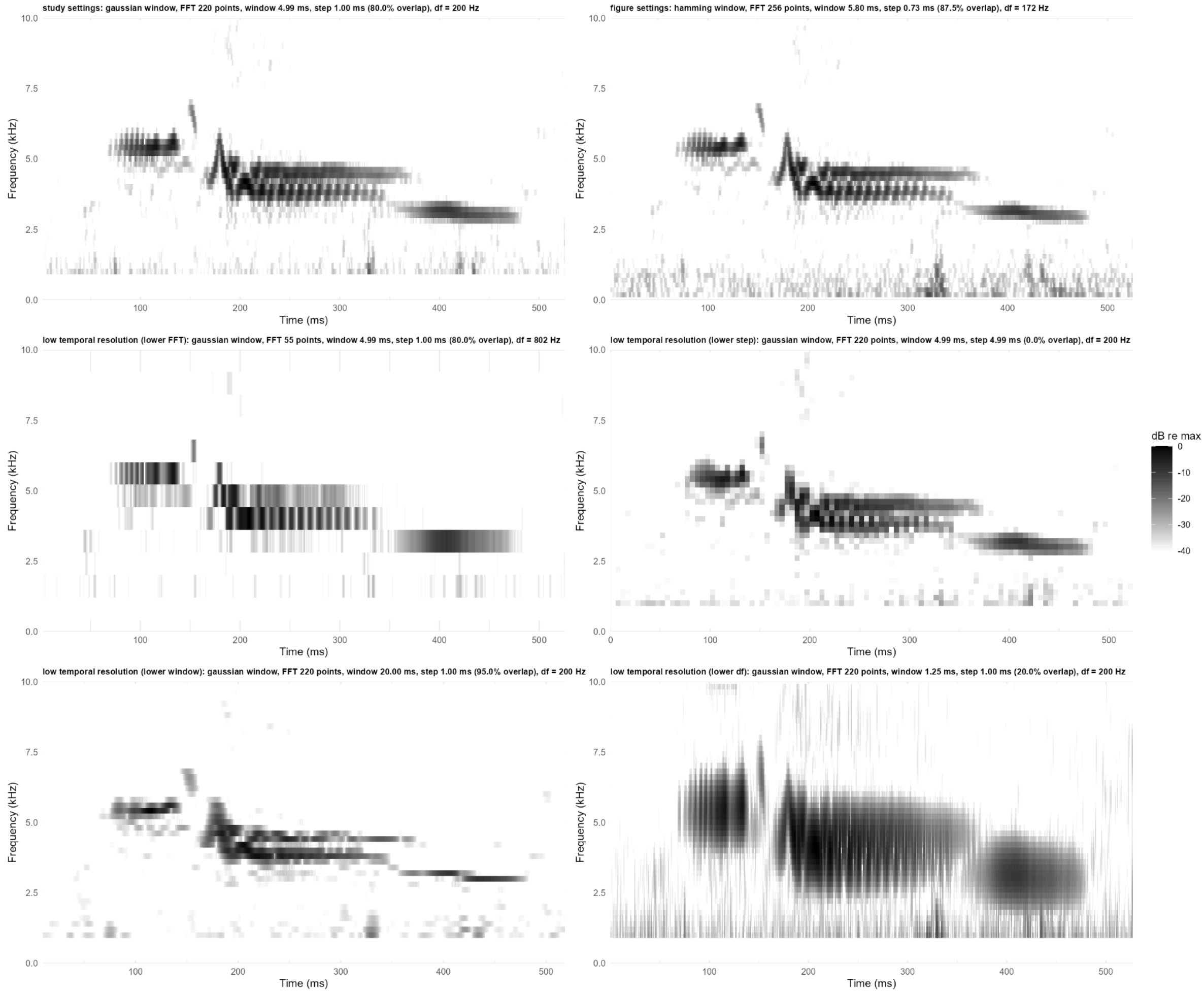

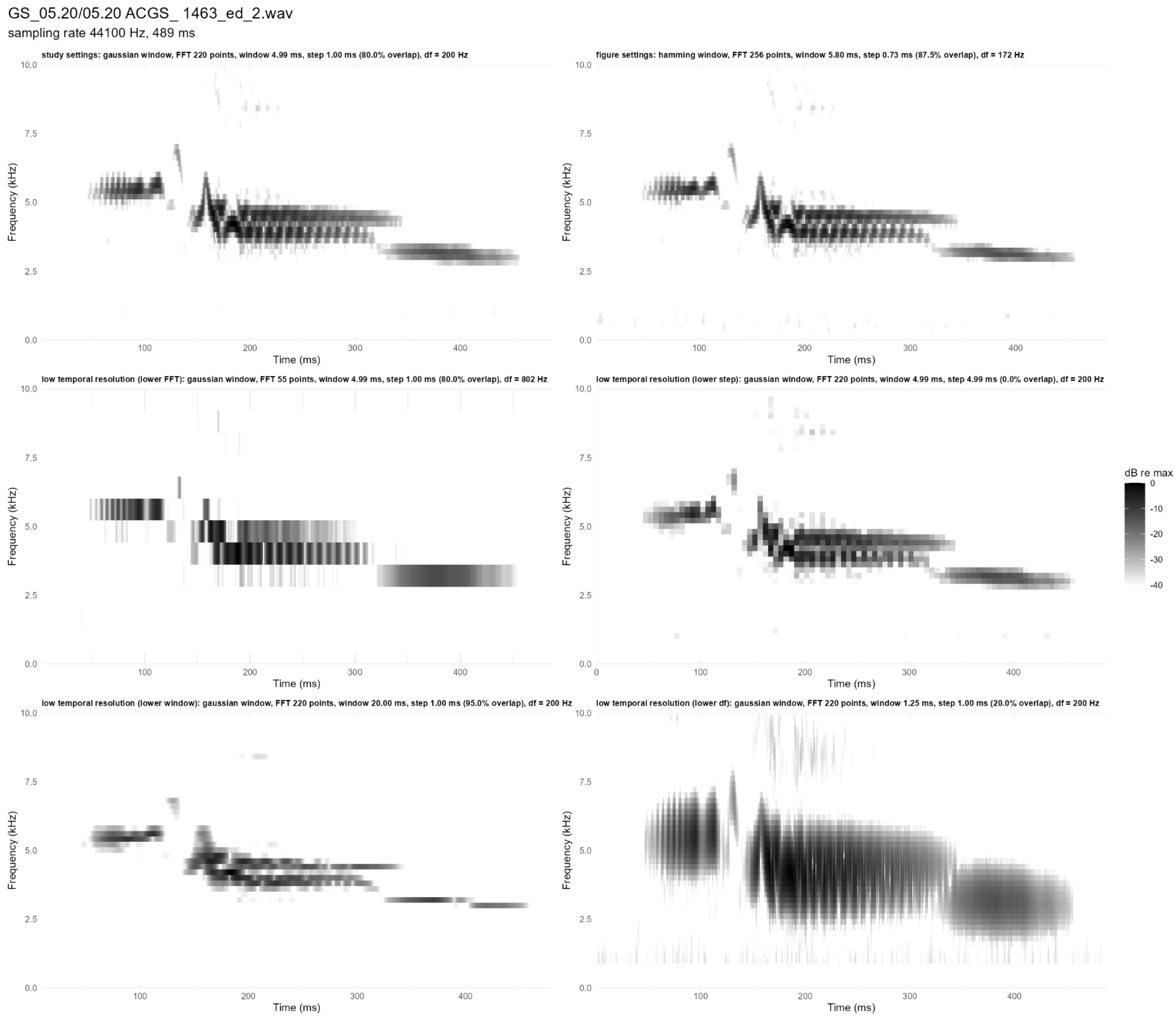

GS_05.20/05.20 ACGS_ 1463_ed_2.wav
sampling rate 44100 Hz, 489 ms
study settings: gaussian window, FFT 220 points, window 4.99 ms, step 1.00 ms (80.0% overlap), df = 200 Hz
figure settings: hamming window, FFT 256 points, window 5.80 ms, step 0.73 ms (87.5% overlap), df = 172 Hz
low temporal resolution (lower FFT): gaussian window, FFT 55 points, window 4.99 ms, step 1.00 ms (80.0% overlap), df = 802 Hz
low temporal resolution (lower step): gaussian window, FFT 220 points, window 4.99 ms, step 4.99 ms (0.0% overlap), df = 200 Hz
low temporal resolution (lower window): gaussian window, FFT 220 points, window 20.00 ms, step 1.00 ms (95.0% overlap), df = 200 Hz
low temporal resolution (lower df): gaussian window, FFT 220 points, window 1.25 ms, step 1.00 ms (20.0% overlap), df = 200 Hz
Frequency (kHz)
Time (ms)
dB re max

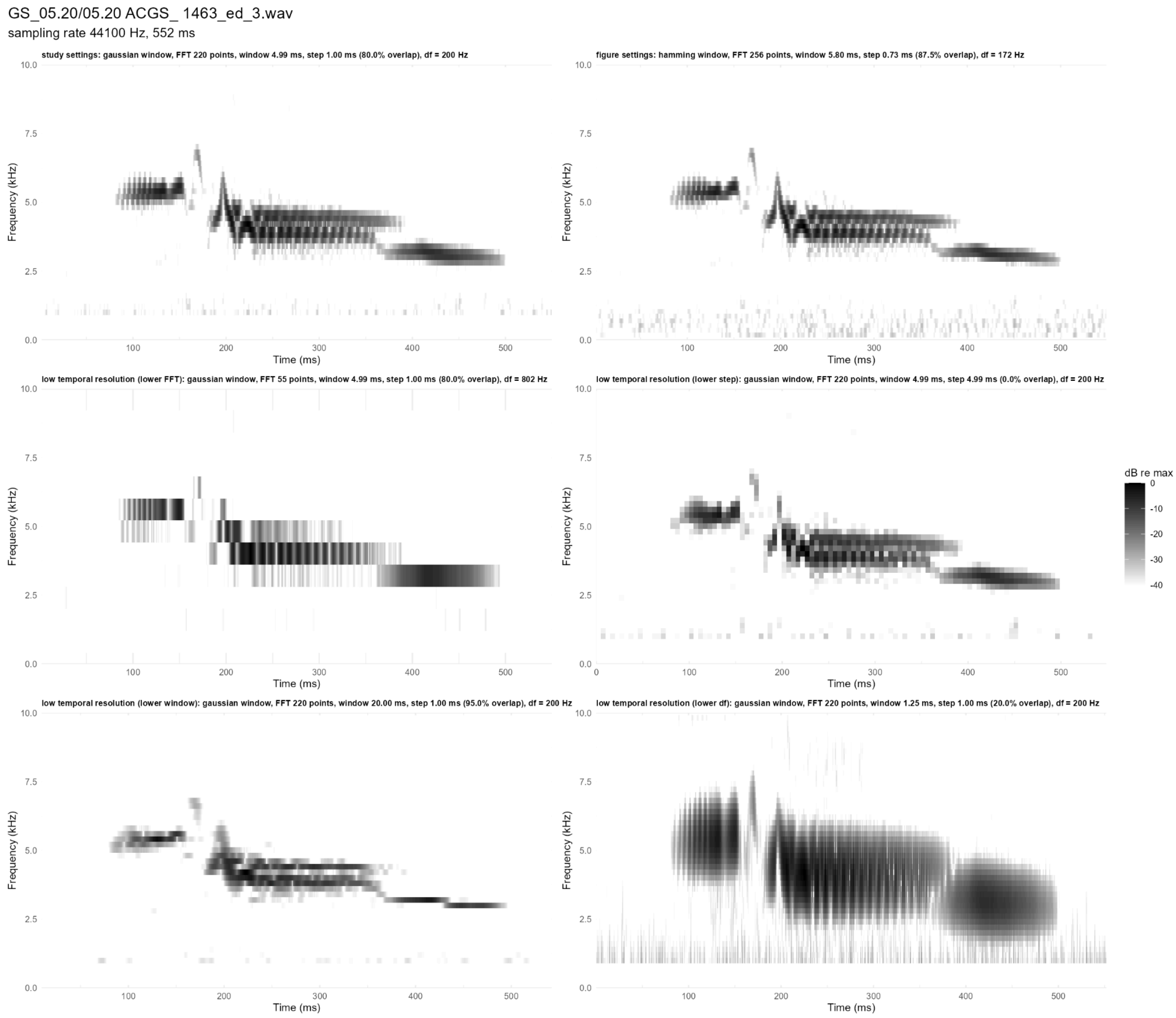
GS_05.20/05.20 ACGS_ 1463_ed_3.wav
sampling rate 44100 Hz, 552 ms
study settings: gaussian window, FFT 220 points, window 4.99 ms, step 1.00 ms (80.0% overlap), df = 200 Hz
figure settings: hamming window, FFT 256 points, window 5.80 ms, step 0.73 ms (87.5% overlap), df = 172 Hz
low temporal resolution (lower FFT): gaussian window, FFT 55 points, window 4.99 ms, step 1.00 ms (80.0% overlap), df = 802 Hz
low temporal resolution (lower step): gaussian window, FFT 220 points, window 4.99 ms, step 4.99 ms (0.0% overlap), df = 200 Hz
low temporal resolution (lower window): gaussian window, FFT 220 points, window 20.00 ms, step 1.00 ms (95.0% overlap), df = 200 Hz
low temporal resolution (lower df): gaussian window, FFT 220 points, window 1.25 ms, step 1.00 ms (20.0% overlap), df = 200 Hz
Frequency (kHz)
Time (ms)
dB re max

GS_06.20/06.20 ACGS_ 1469_ed_1.wav

sampling rate 44100 Hz, 428 ms

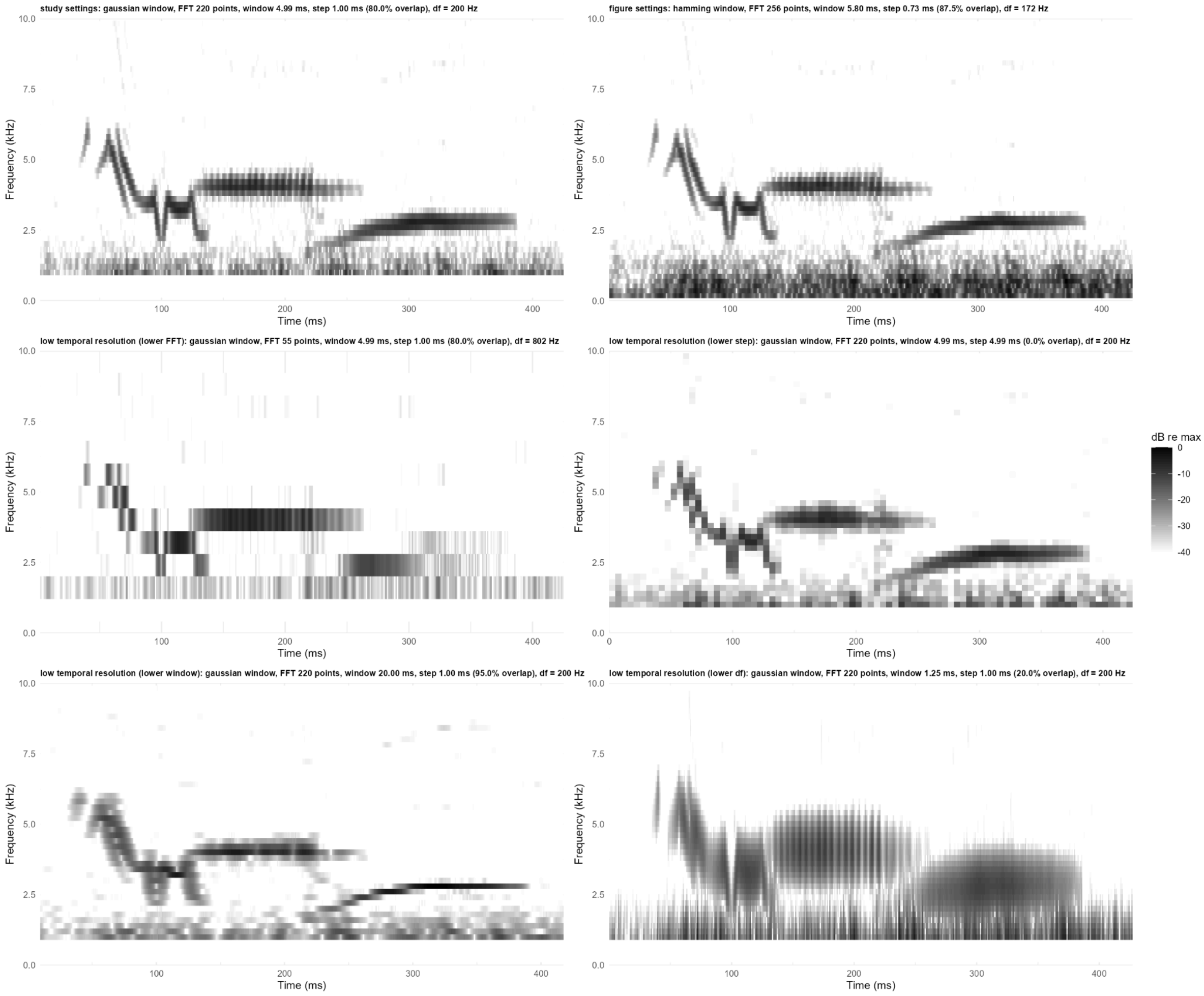

GS_06.20/06.20 ACGS_ 1469_ed_2.wav

sampling rate 44100 Hz, 433 ms

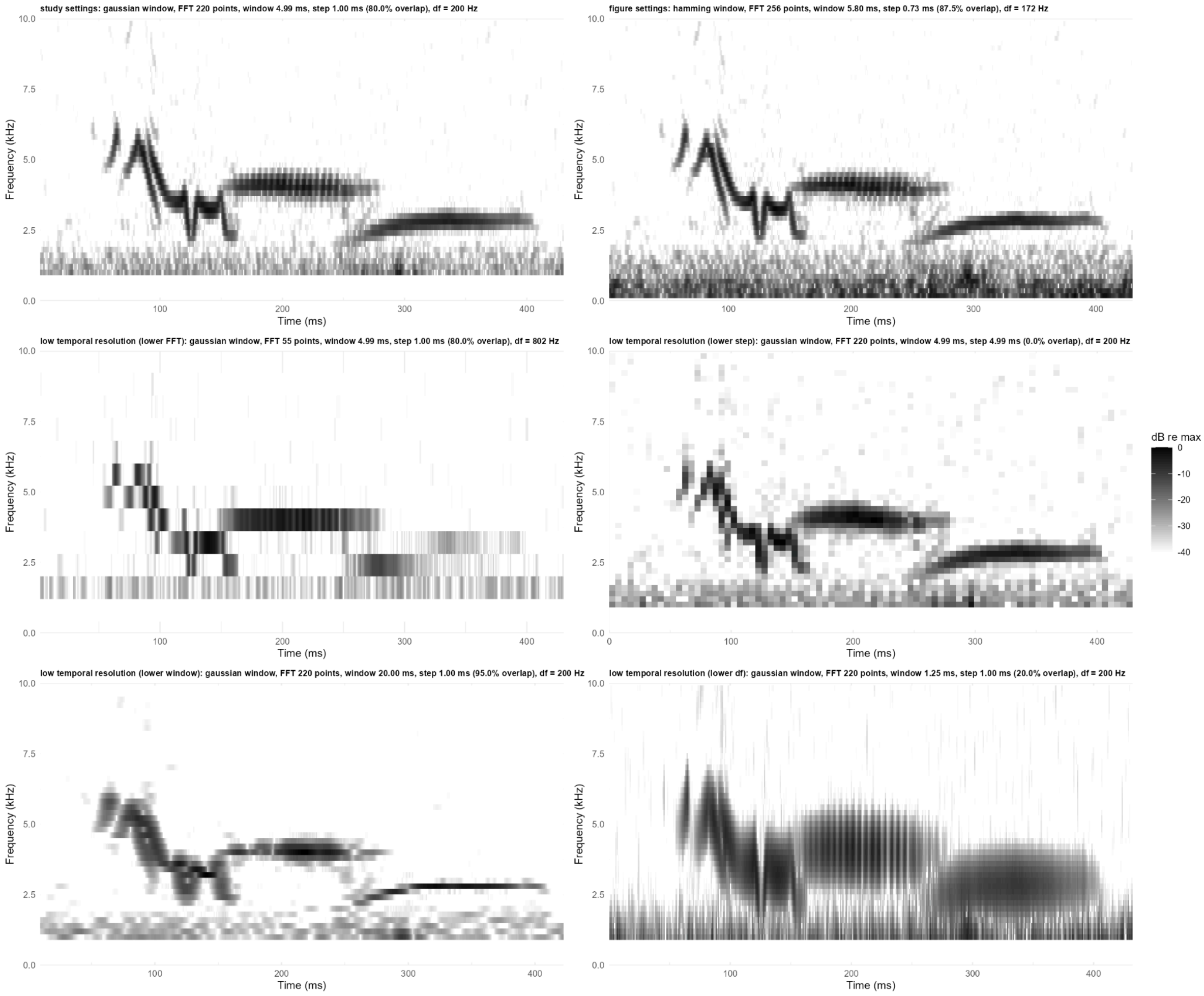

GS_06.20/06.20 ACGS_ 1469_ed_6.wav

sampling rate 44100 Hz, 439 ms

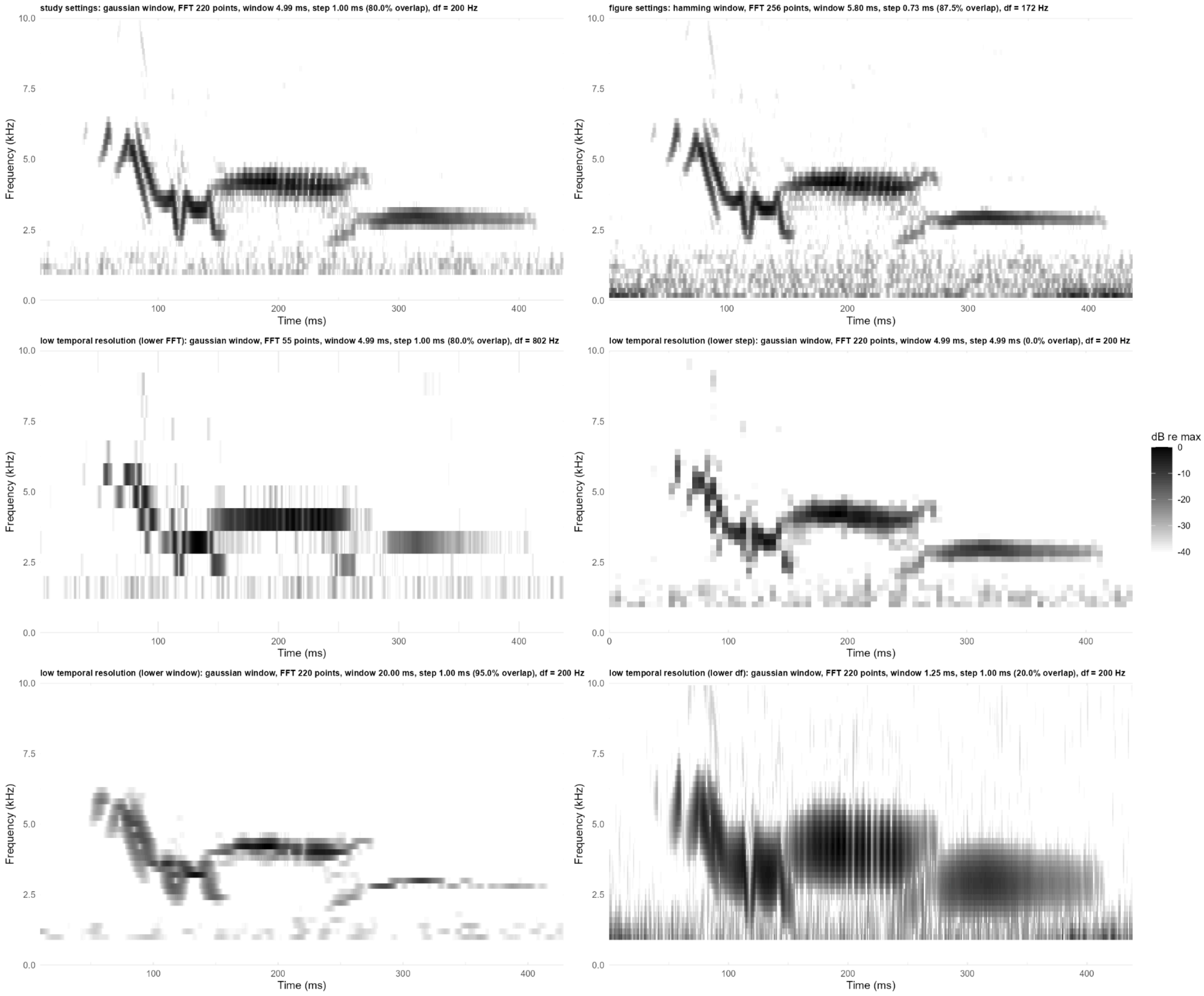

GS_76.20/76.20 ACGS_ 1632_ed_1.wav

sampling rate 44100 Hz, 481 ms

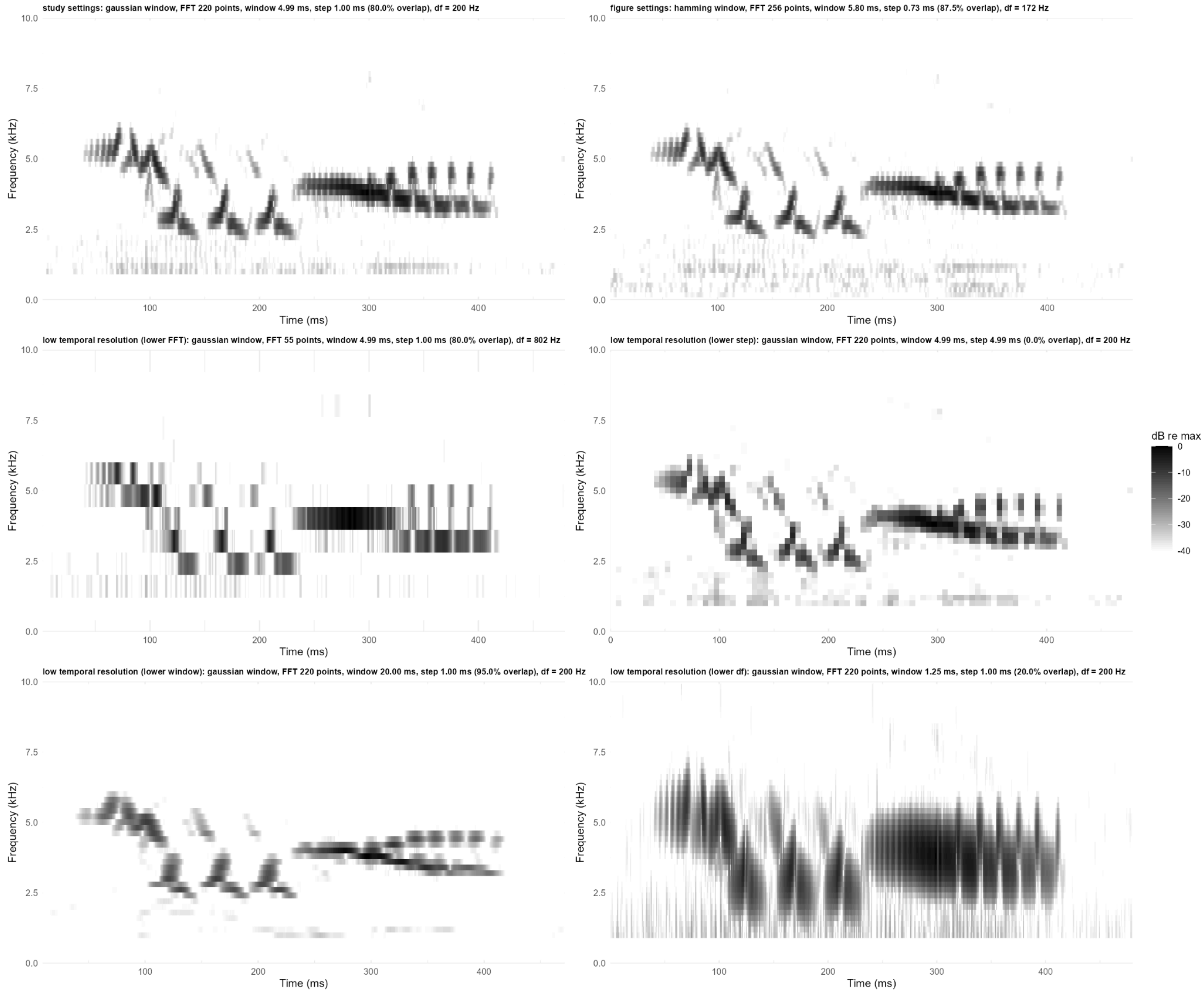

Supplement: Supplementary file 1 [file NYAS-1565-0-s001.pdf]
